# Supplementary material for: Human Pancreatic β Cell lncRNAs Control Cell-Specific Regulatory Networks
Source: Cell Metab. 2017 Feb 7;25(2):400–11. doi: 10.1016/j.cmet.2016.11.016 (PMC5300904; doi:10.1016/j.cmet.2016.11.016)
Supplement: Document S1. Supplemental Experimental Procedures and Figures S1–S7 [file mmc1.pdf]

Cell Metabolism, Volume *25*

Supplemental Information

Human Pancreatic β Cell lncRNAs Control Cell-Specific Regulatory Networks

Ildem Akerman, Zhidong Tu, Anthony Beucher, Delphine M.Y. Rolando, Claire Sauty-Colace, Marion Benazra, Nikolina Nakic, Jialiang Yang, Huan Wang, Lorenzo Pasquali, Ignasi Moran, Javier Garcia-Hurtado, Natalia Castro, Roser Gonzalez-Franco, Andrew F. Stewart, Caroline Bonner, Lorenzo Piemonti, Thierry Berney, Leif Groop, Julie Kerr-Conte, Francois Pattou, Carmen Argmann, Eric Schadt, Philippe Ravassard, and Jorge Ferrer

Figure S1. Loss of function of beta cell lncRNAs, related to Figure 1 (part 1 of 6)

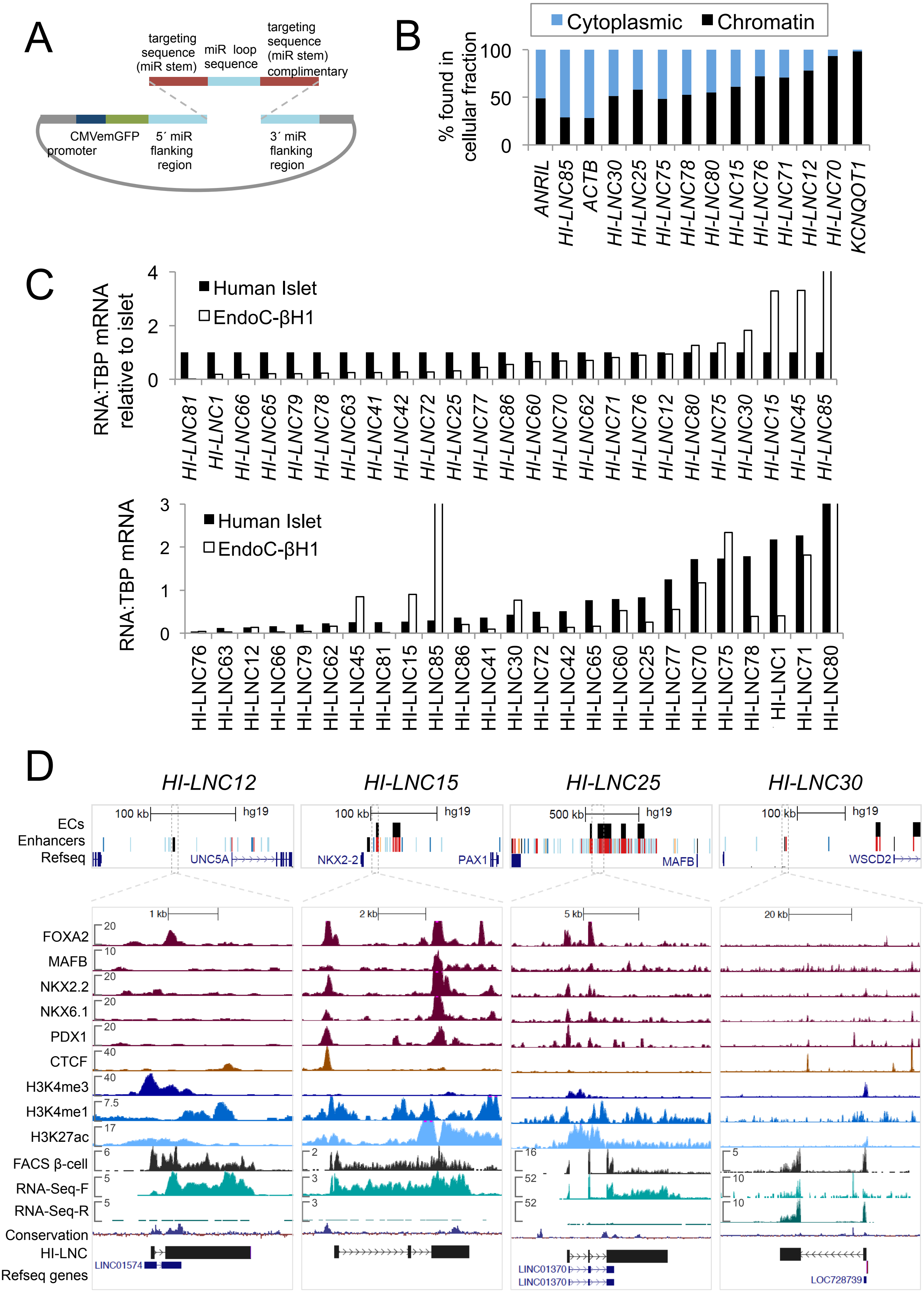

Figure S1. Loss of function of beta cell lncRNAs, related to Figure 1 (part 2 of 6)

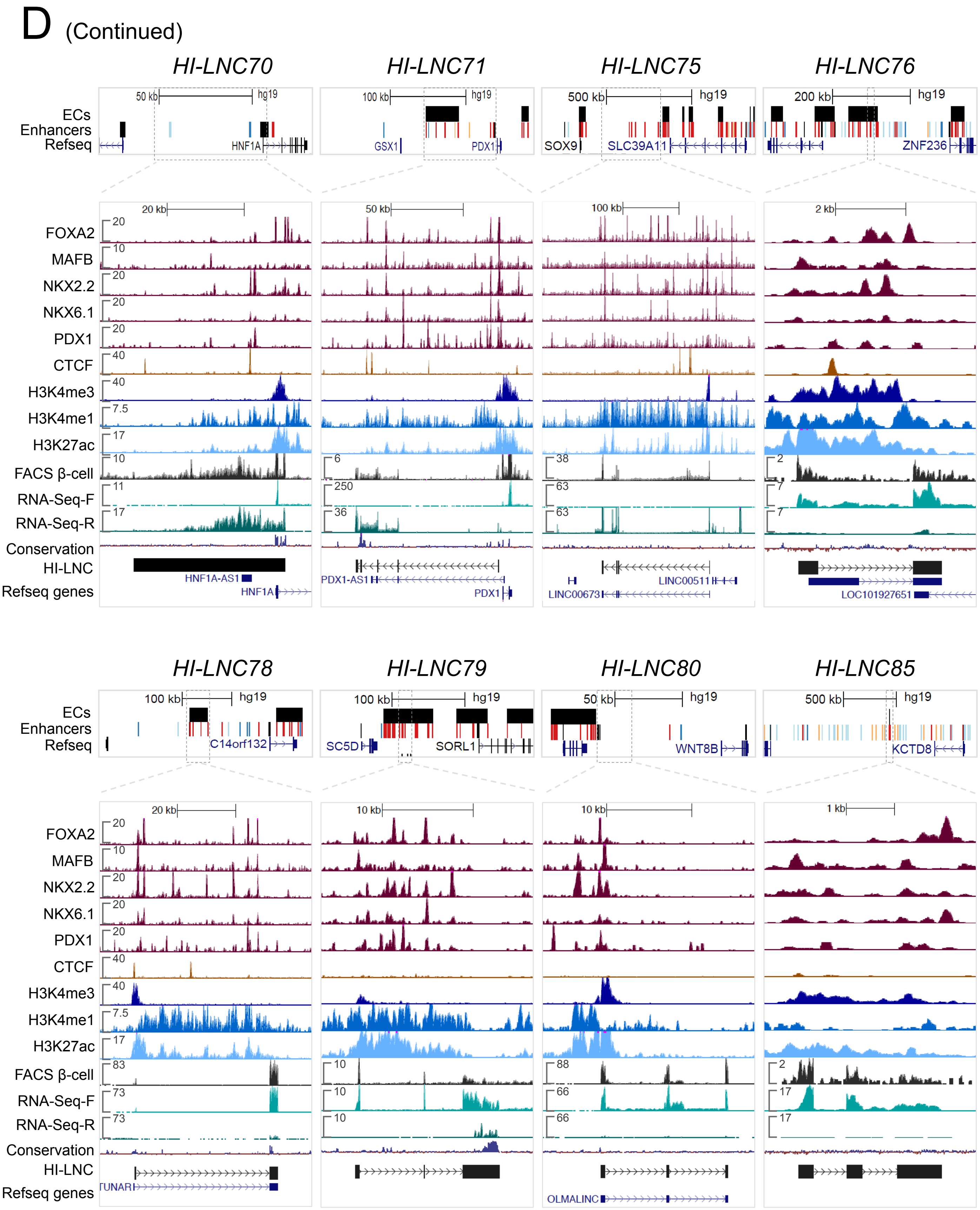

Figure S1. Loss of function of beta cell lncRNAs, related to Figure 1 (part 3 of 6)

E

		Replicate1		Replicate2	
		amiRNA-1	ami-RNA-2	amiRNA-1	ami-RNA-2
TFs	GLIS3	0.31	0.33	0.34	0.32
	HNF1a	0.50	0.47	0.50	0.52
	MAFB	0.53	0.41	0.51	0.44
	NKX2.2	0.50	0.43	0.12	0.33
	PDX1	0.34	0.44	0.35	0.50
HI-LNCs with 2 amiRNAs per target	HI-LNC12	0.44	0.48	0.51	0.54
	HI-LNC15	0.13	0.39	0.12	0.40
	HI-LNC30	0.27	0.24	0.30	0.47
	HI-LNC75	0.45	0.45	0.48	0.44
	HI-LNC76	0.46	0.35	0.43	0.55
	HI-LNC78	0.38	0.30	0.46	0.31
	HI-LNC80	0.34	0.40	0.35	0.46
		Replicate1	Replicate2	Replicate3	
HI-LNCs with one amiRNA per target	HI-LNC25	0.42	0.33	0.39	
	HI-LNC70	0.48	0.48	na	
	HI-LNC71	0.14	0.25	0.14	
	HI-LNC79	0.18	0.17	0.16	
	HI-LNC85	0.21	0.17	0.25	

F

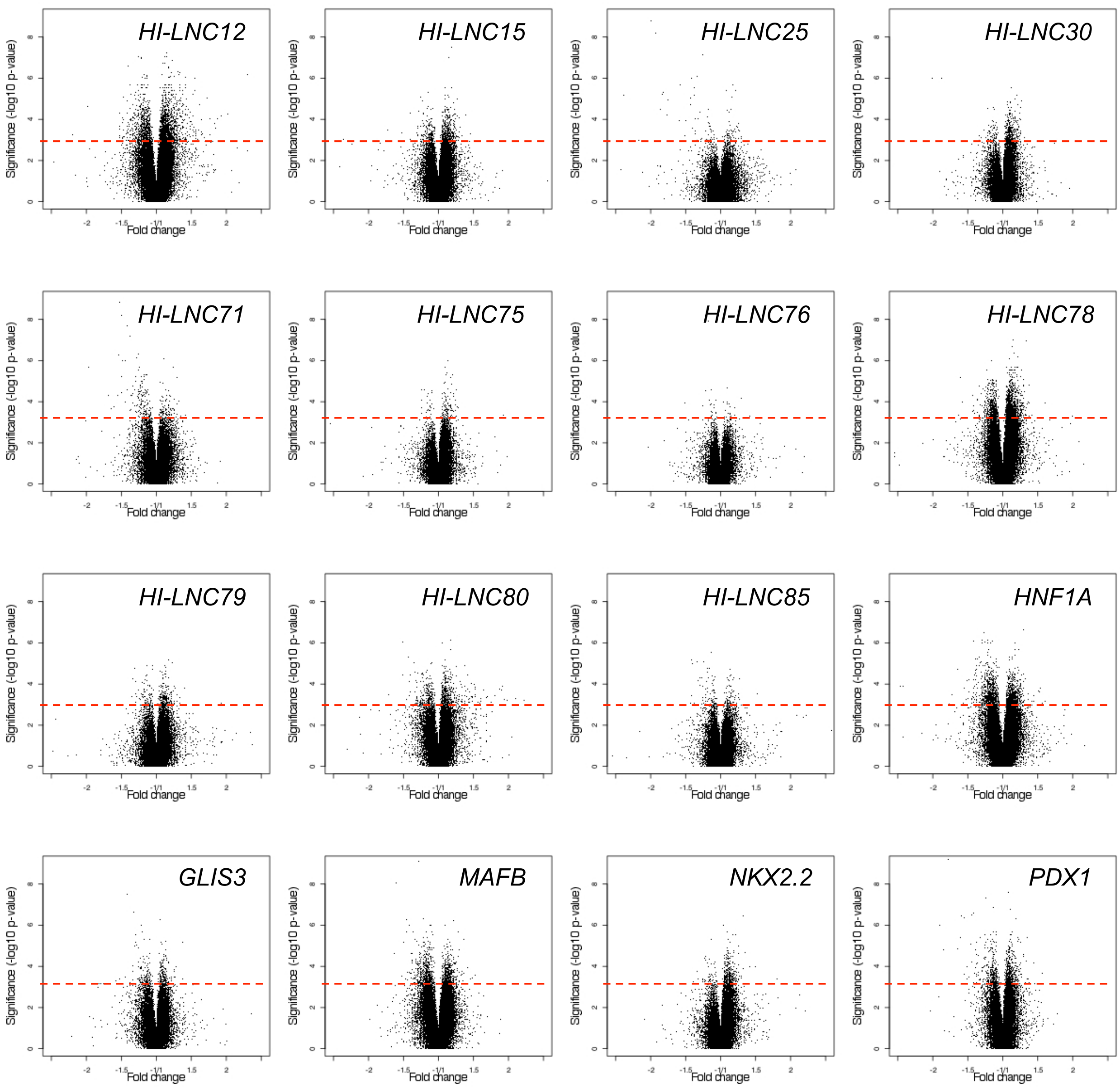

Figure S1. Loss of function of beta cell lncRNAs, related to Figure 1 (part 4 of 6)

G

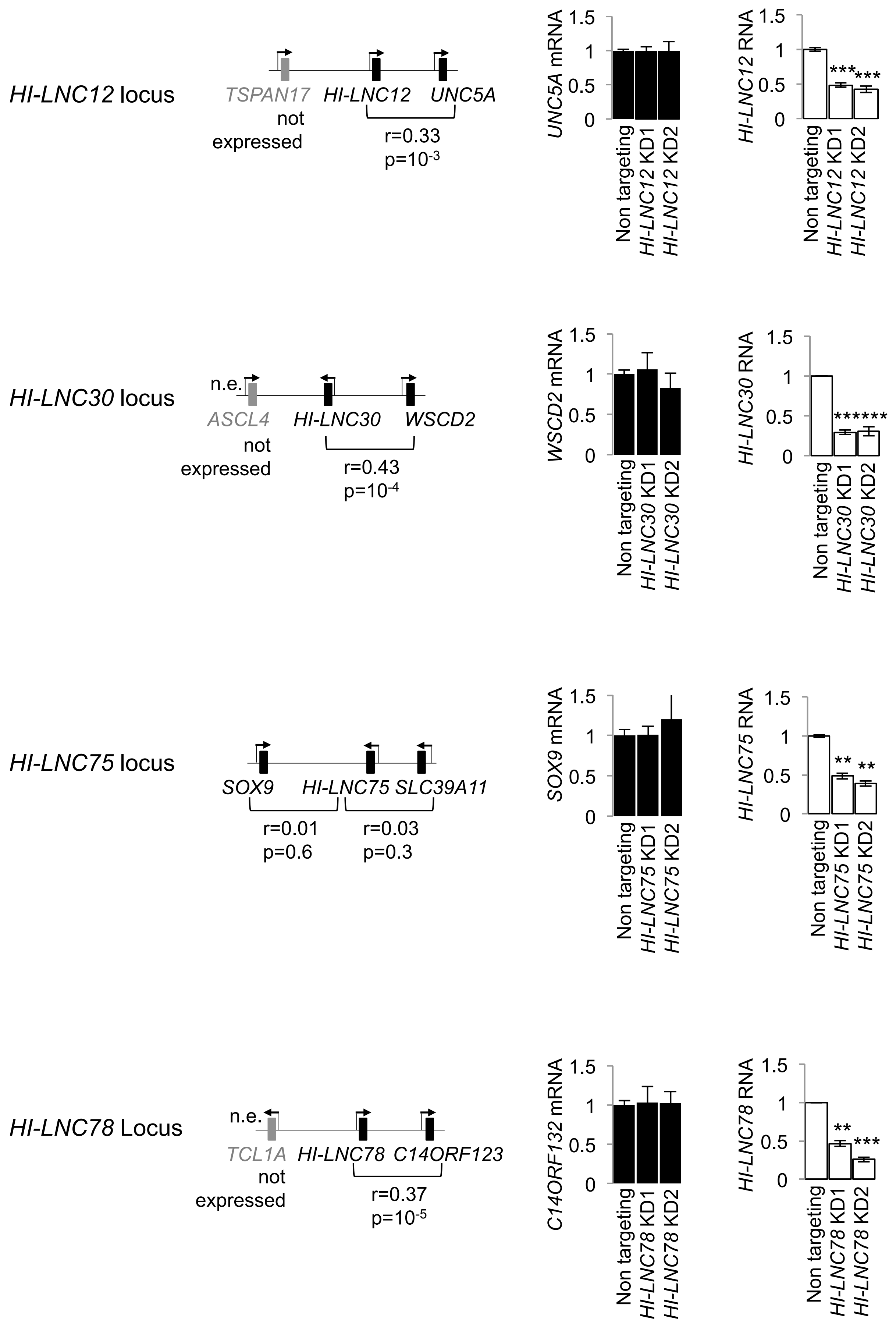

Figure S1. Loss of function of beta cell lncRNAs, related to Figure 1 (part 5 of 5)

G(Continued)

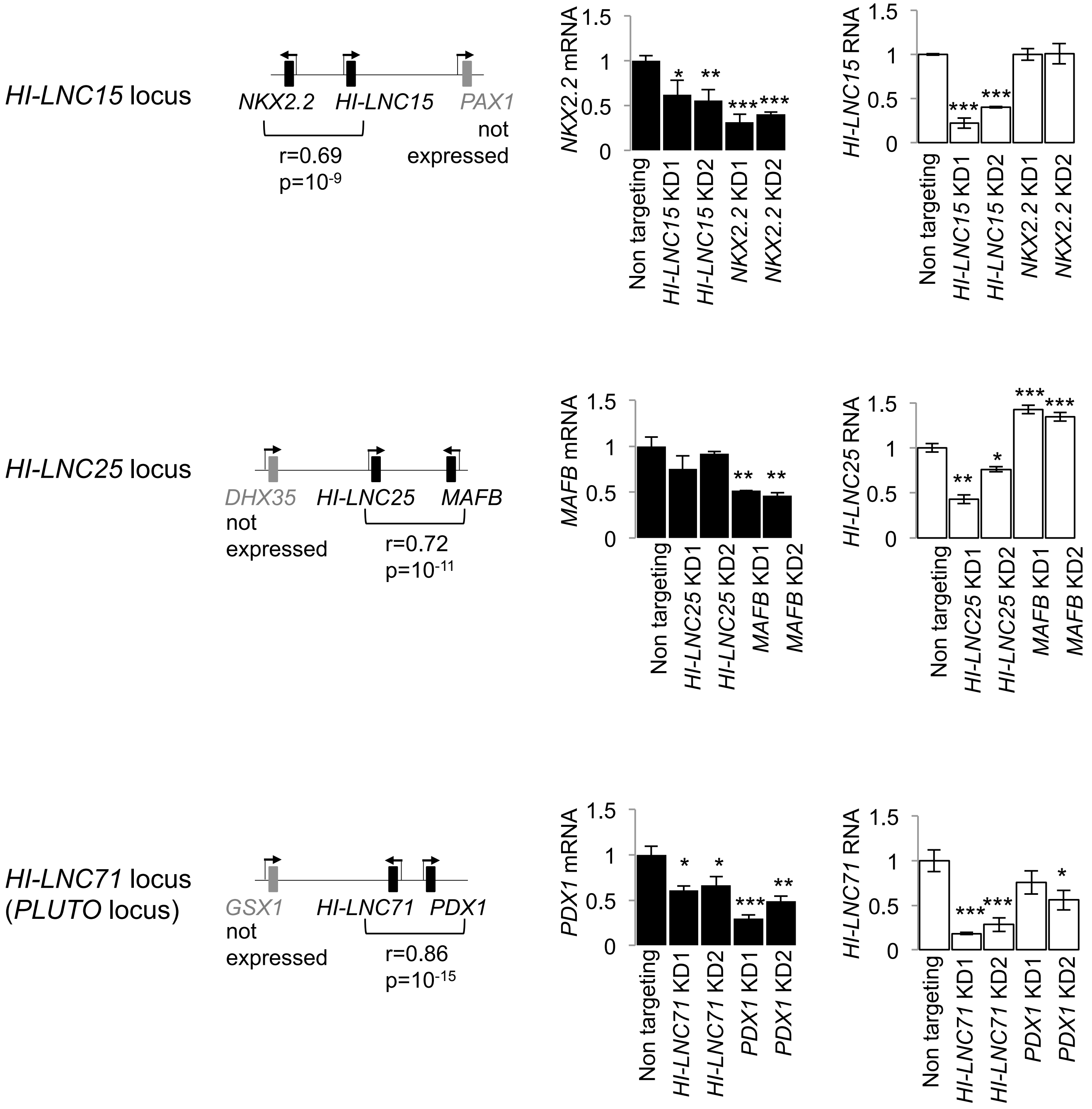

Figure S1. Loss of function of beta cell lncRNAs, related to Figure 1 (part 6 of 6)

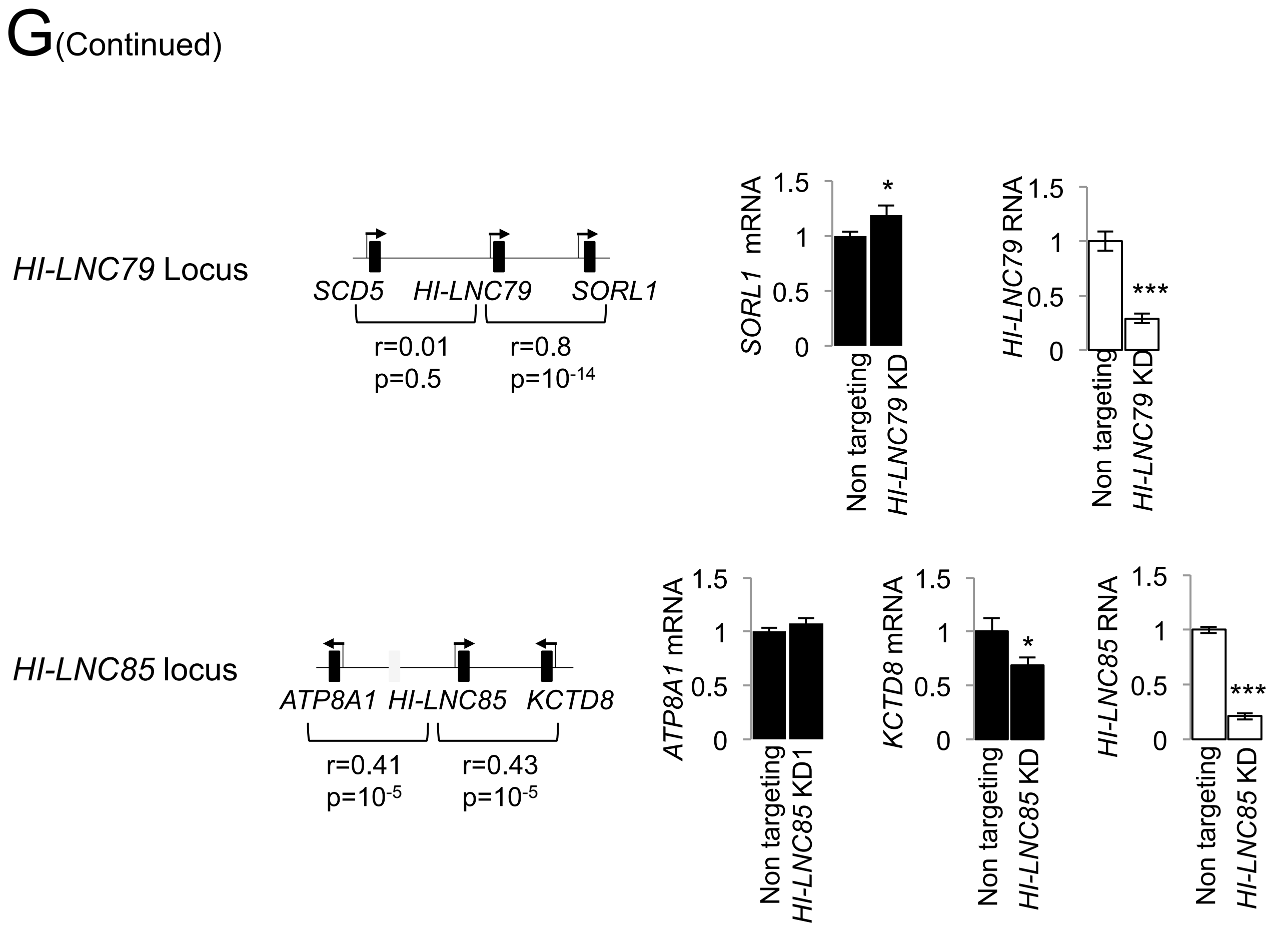

Figure S1. Loss of function of beta cell lncRNAs (A) A schematic of the vector used for generating amiRNAs. **(B)** Subcellular fractionation of islet lncRNAs. in EndoC-βH3 cells. **(C)** Relative expression of 25 shortlisted lncRNAs in human islets and in EndoC-βH1 cells. RNA levels were analyzed by qPCR, normalized to *TBP* mRNA (lower panel) and then to the levels in human islets (upper panel). **(D)** Genome browser images of the 12 human beta cell lncRNA genes selected for functional analysis. RNA-seq F:forward strand, R:reverse strand, axis represent RPM. HI-LNC track shows prevalent gene structures in human islet RNA. The top inset shows islet enhancer clusters (ECs), islet enhancers in red lines, and RefSeq annotations of nearby genes. **(E)** Knockdown efficiency for each amiRNA. The fraction of RNA remaining is shown. **(F)** Volcano plots of differential gene expression. Red lines indicate $p=10^{-3}$. **(G)** Regulatory interactions between islet lncRNAs and neighboring genes. Knowckdown experiments show that *HI-LNC15*, *HI-LNC79*, *HI-LNC85,* and *HI-LNC71(PLUTO)* regulate a neighboring gene (right panels), and furthermore display a high expression correlation with the neighboring gene in a panel of human islets (left panels). Other tested lncRNAs (*HI-LNC12, HI-LNC25, HI-LNC30, HI-LNC75, HI-LNC78*) do not affect adjacent genes, and display comparatively lower correlation of gene expression with the neighboring genes in islet samples. The left panel depicts Pearson correlation and associated p values for RNA levels in 64 human islet samples. n.e. indicates that the gene is not expressed in islets. The right panel depicts RNA levels normalized to *TBP* mRNAsequences used for knockdown of and then to control amiRNA samples. KD1 and KD2 represent different amiRNAs for indicated transcripts. Note that *HI-LNC25* and *MAFB* are highly co-expressed in human islets despite that *HI-LNC25* does not significantly regulate *MAFB* expression, although *MAFB* regulates *HI-LNC25*. Error bars denote ±SEM. ***p<0.001, **p<0.01, *p<0.05, Student´s t test, n=3 independent experiments.

Figure S2. Knockdown of lncRNAs causes impaired insulin secretion (related to Figure 2)

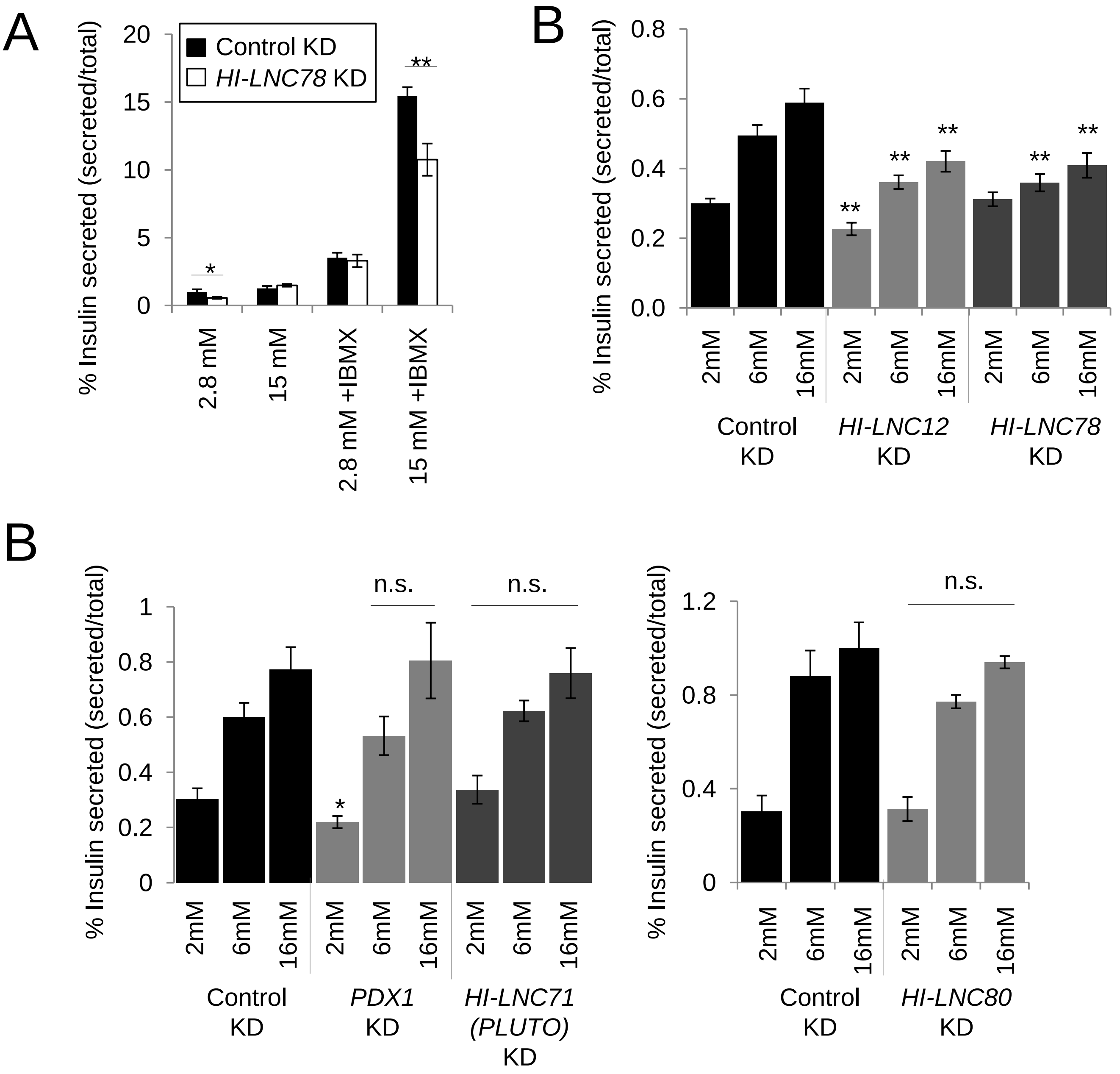

Figure S2. Knockdown of lncRNAs causes impaired insulin secretion. (A) Glucose stimulated insulin secretion was tested on T-antigen excised EndoC-βH3 cells after transduction with amiRNAs targeting *HI-LNC78* or controls. Secreted insulin content was normalized to total insulin content and expressed as fold change over control amiRNA treatment at 2.8 mM glucose. Each bar represents an average of two independent amiRNA vectors and 12 independent wells, from two independent experiments. Error bars represent ±SEM, ** $p<0.01$, *$p<0.05$ (Student's t-test). (B) Glucose-stimulated insulin secretion was measured in EndoC-βH3 cells 80 hours after nucleofection with locked nucleic acid (LNA) oligonucleotides to provide independent confirmation for amiRNA-based inhibition studies. Secreted insulin (ng/ml) was normalized to total insulin content and expressed as a percentage of total insulin content.. Each bar represents an average of two independent LNAs from four independent experiments. Error bars represent ±SEM. *$p<0.05$. ** $p<0.01$, n.s.=not significant, Student's t test, compared with control knock-downs in the same glucose concentration.

Figure S3. Knockdown of human islet TFs and lncRNAs leads causes shared transcriptional phenotypes, related to Figure 3

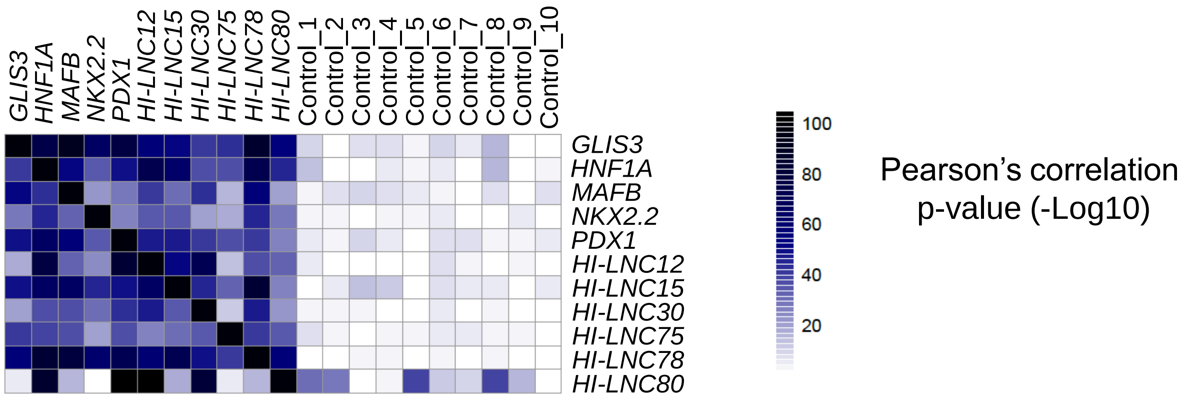

Figure S3. Knockdown of human islet TFs and lncRNAs leads causes shared transcriptional phenotypes. Heatmap of p values for Pearson´s correlation analysis of changes in gene expression for all pairwise comparisons of knockdowns that showed transcriptional phenotypes. The fold change of top 250 targets of each knockdown (vertical orientation) was correlated to the fold change of the same genes in other knockdowns and control experiments (horizontal orientation).

Figure S4. Islet specific TFs and lncRNAs regulate cell-specific programs, related to Figure 4

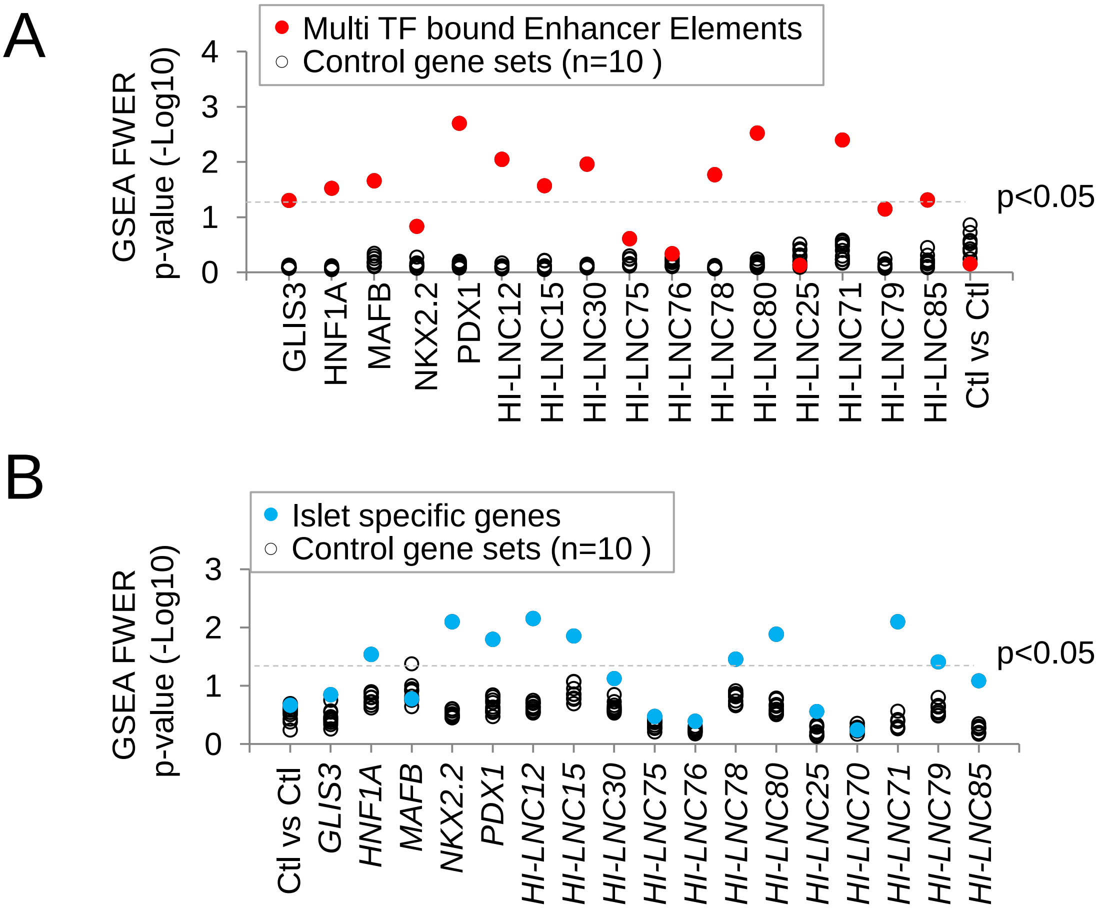

Figure S4. Islet-specific TFs and lncRNAs regulate cell-specific cis-regulatory programs. (A) TF and lncRNA-dependent genes are associated with enhancers that show high occupancy by islet-specific TFs. Gene Set Enrichment Analysis (GSEA) showed that a set of 1137 genes that are associated with enhancers bound by multiple islet TFs (red dots, **Table SX**) were enriched among genes that were downregulated upon knockdown of islet TFs and lncRNAs, whereas this was not observed among 10 size-matched control gene sets that are expressed at similar levels as the enhancer-associated genes (black dots). Islet enhancers bound by four or more islet TFs have been shown to be associated with core lineage-specific regulatory genes (Pasquali et al., 2014). (B) TF and lncRNA-dependent genes are often islet specific. GSEA analysis was performed with 700 genes that show highest expression compared to 16 other tissues (blue dots, **Table SX**) or 10 control size-matched gene sets that are expressed at similar levels (black dots). Y-axes represent GSEA FWER p-values (-Log10).

Figure S5. Co-expression analysis of human islet lncRNAs, related to Figure 5 (part 1 of 2)

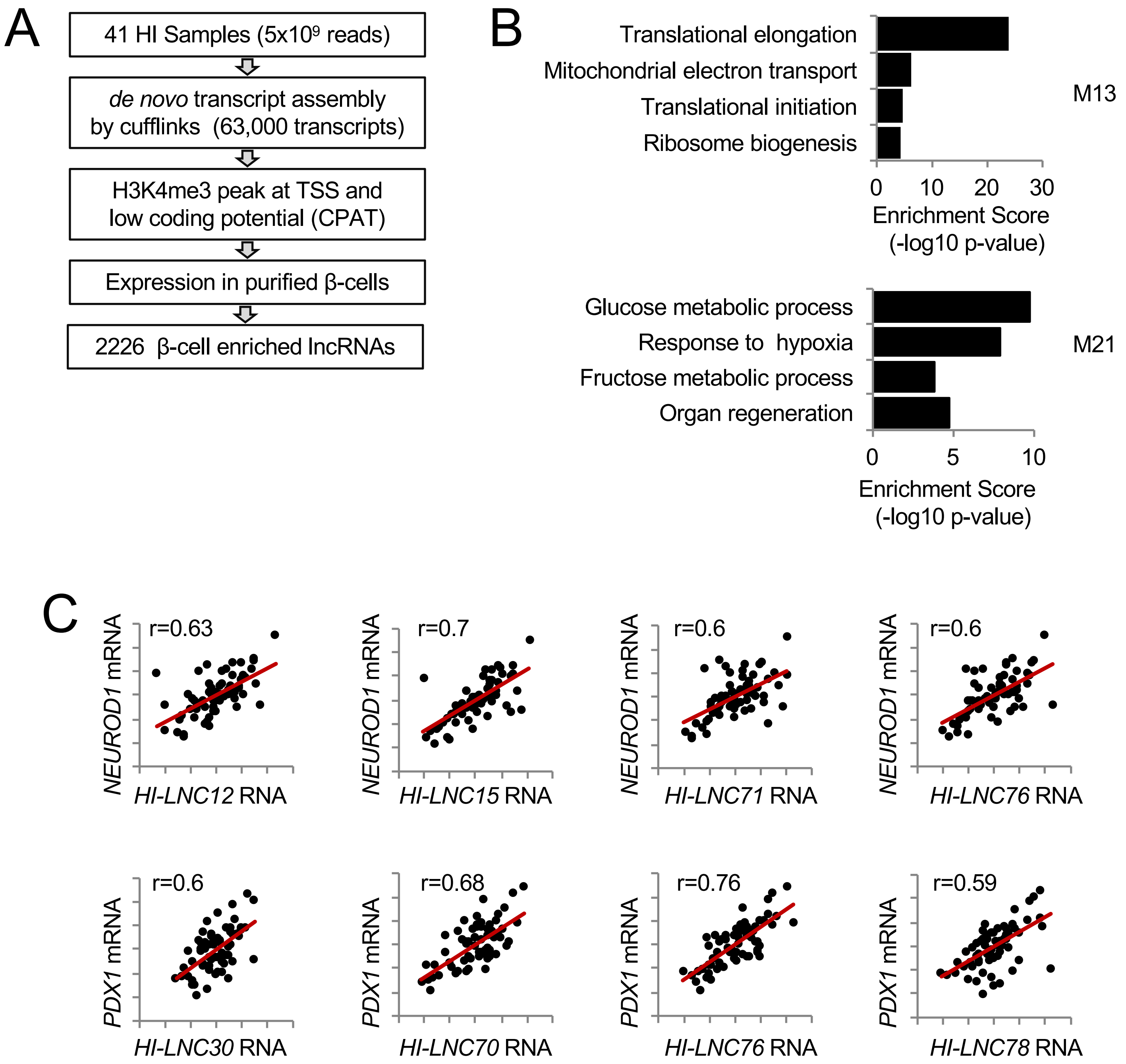

D

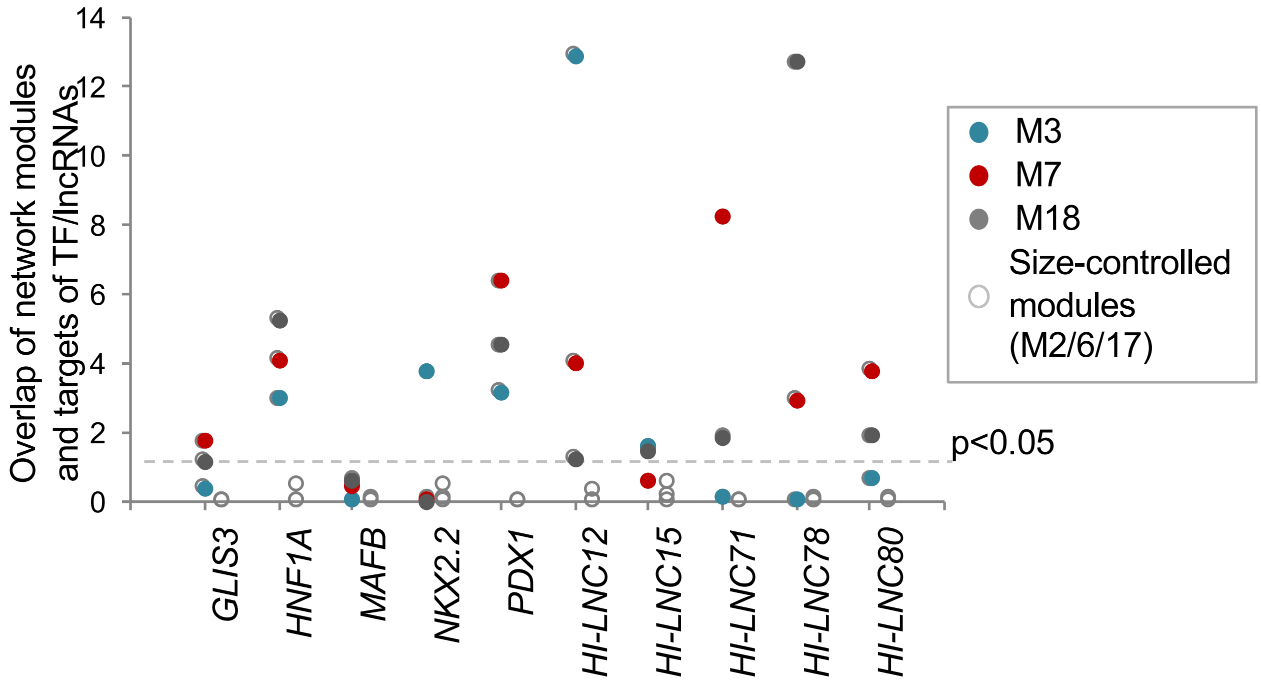

Figure S5. Co-expression analysis of human islet lncRNAs. (A). Schematic of *de novo* lncRNA annotation pipeline. To expand the previously defined list of islet lncRNAs and enhance their annotation accuracy (Moran et al, 2012), RNA from 41 human islet samples was sequenced directionally, generating nearly 5 billion reads. Cufflinks was then used to assemble *de novo* transcripts, which led to ~63,000 transcripts that were not known coding genes. These transcripts were further filtered for (i) low coding potential based on CPAT, (ii) K4me3 enrichment at a putative promoter region. (iii) expression in FACS purified β-cell samples as well as FACS purified acinar/β-cell ratio of < 3. Multiple splice variants of the same gene were collapsed into a single gene, resulting in 2226 islet lncRNA genes (**Table S3**). (**B**) Module M13 is enriched in translation and ribosomal related gene ontology annotations while M21 is enriched in terms related to glucose metabolic process (DAVID analysis, biological process). (**C**) Examples of β-cell lncRNAs that show a high correlation of expression with islet TFs *PDX1* and *NEUROD1* across 64 human islet samples. The axes are gene expression values normalized across all samples. (**D**) TFs and lncRNAs often regulate genes from lncRNA-enriched co-expression modules. Y-axis represents p value (-Log10) of hypergeometric test of overlap between the gene sets that show significant downregulation upon knockdown of indicated TF or lncRNAs (at p<10-3), and the cell-specific co-expression modules enriched in lncRNAs (M3, M7, M18). As controls, we examined the 3 modules (M2, M6, M17) that had the closest number of genes as lncRNA-enriched modules.

Figure S6. LncRNA *PLUTO* in mouse and human islets (related to Figure 6, page 1 of 3)

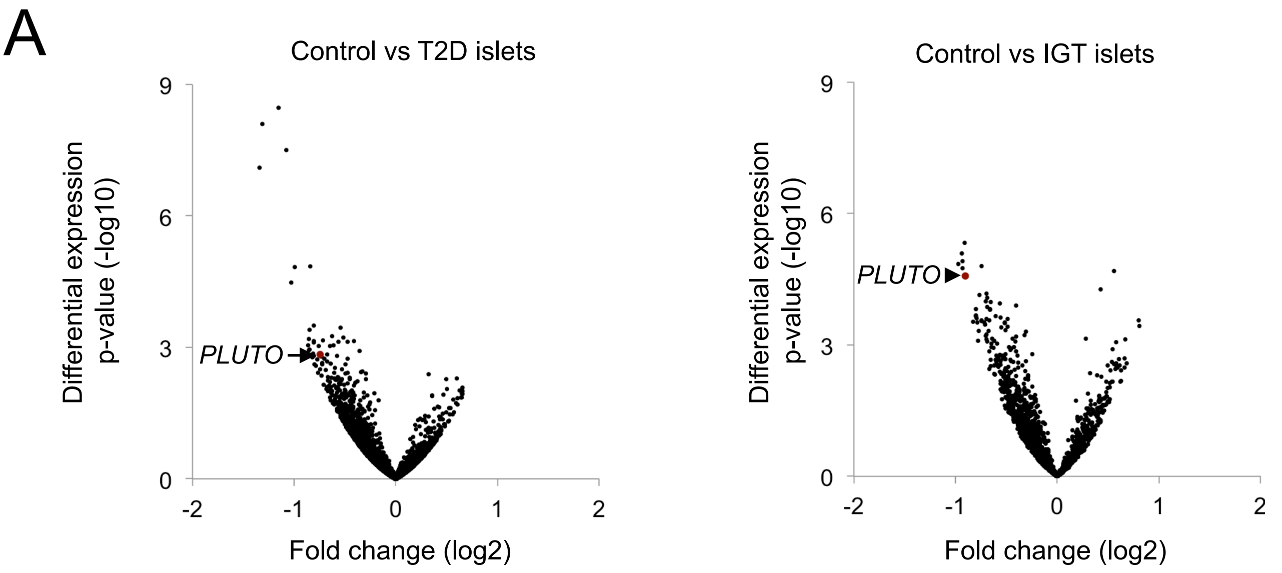

Figure S6. LncRNA *PLUTO* in mouse and human islets (related to Figure 6, page 2 of 3)

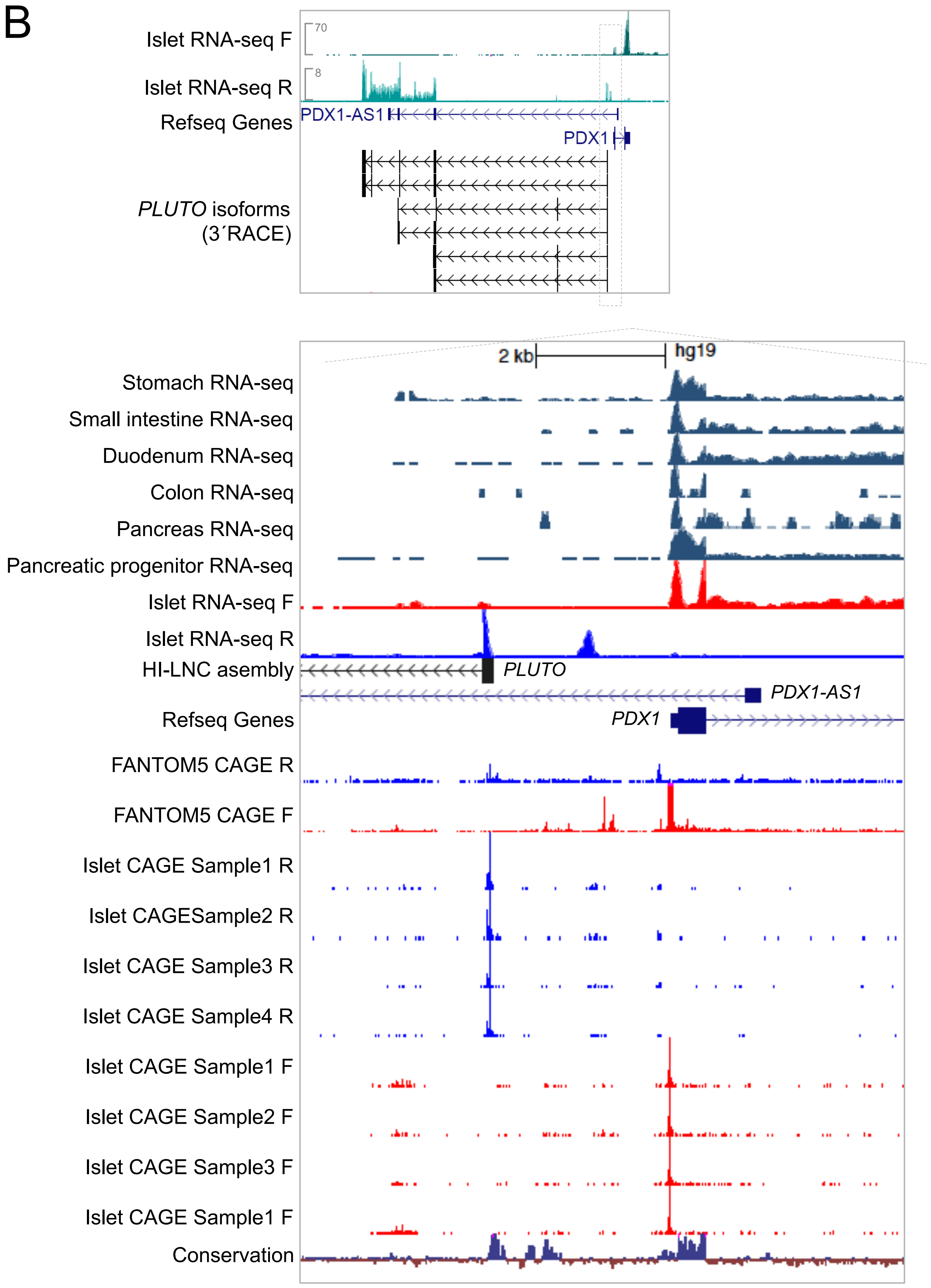

Figure S6. LncRNA *PLUTO* in mouse and human islets (related to Figure 6, page 3 of 3)

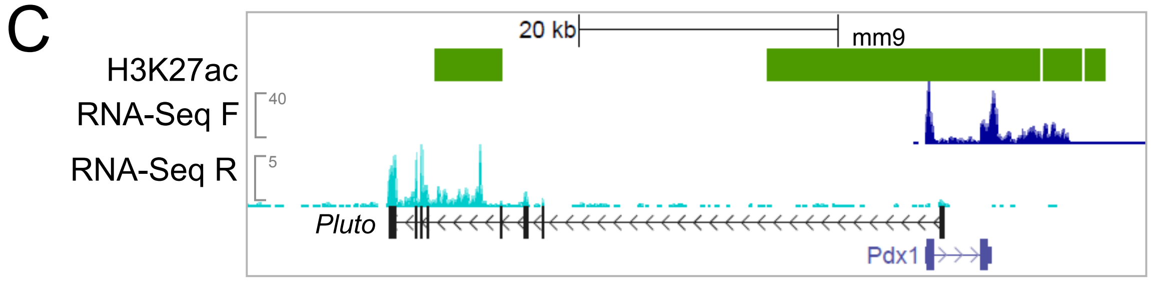

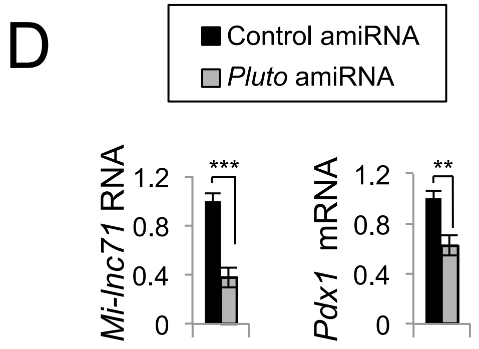

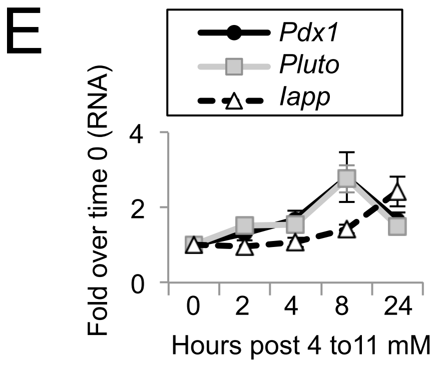

Figure S6. LncRNA *PLUTO* in mouse and human islets
(A) Differential expression analysis of human beta cell lncRNAs from control (n=50) versus T2D (n=10) or IGT (n=15) islet RNA-seq samples. Volcano plots of nominal p-value (-log10) vs Log2 fold change of expression (T2D or IGT over control) is shown. (B) Human *PLUTO* is a multi-isoform transcript. The schematic represents the isoforms cloned from 3´RACE assay. Detailed lower panel shows RNA-seq from Human Proteome Atlas (HPA) tissues, as well as pancreatic progenitor and human islet. Islet RNA-seq track represents merged reads from 56 different islet samples totaling 7.1 billion aligned, stranded reads (scales represent RPM). Shown in blue (reverse) and red (forward) are total CAGE tags from FANTOM5 consortium. CAGE tags from four independent islet samples are also shown. Conservation track shows vertebrate conservation (PhyloP). (C) Schematic of the mouse *Pdx1/Pluto* locus, showing H3K27Ac-enriched regions in mouse islets, and RNA-seq data for the forward and reverse strands. Shown in black is a cloned isoform of *Pluto*. (D) In a mouse beta cell line (MIN6), downregulation of *Pluto* using amiRNA results in reduced *Pdx1* mRNA. RNA values are normalized to *TBP* and then to control amiRNA. Error bars represent ±SEM, $**p<10^{-3}$, $*p<0.05$, Student´s t-test. (E) In mouse islets, *Pdx1* and *Pluto* RNAs are co-regulated in response to a shift in glucose levels. Mouse islets were harvested and incubated in media with 4 mM glucose for 2 days. Islets were then shifted to a higher glucose concentration (11 mM) for indicated periods of time during which both *Pdx1* and *Pluto* RNA is upregulated with similar kinetics. Shown as control, *Iapp* mRNA levels are induced with different dynamics (n=3).

Figure S7. Effects of *PLUTO* on chromatin modification landscape of *PDX1* enhancers (related to Figure 7)

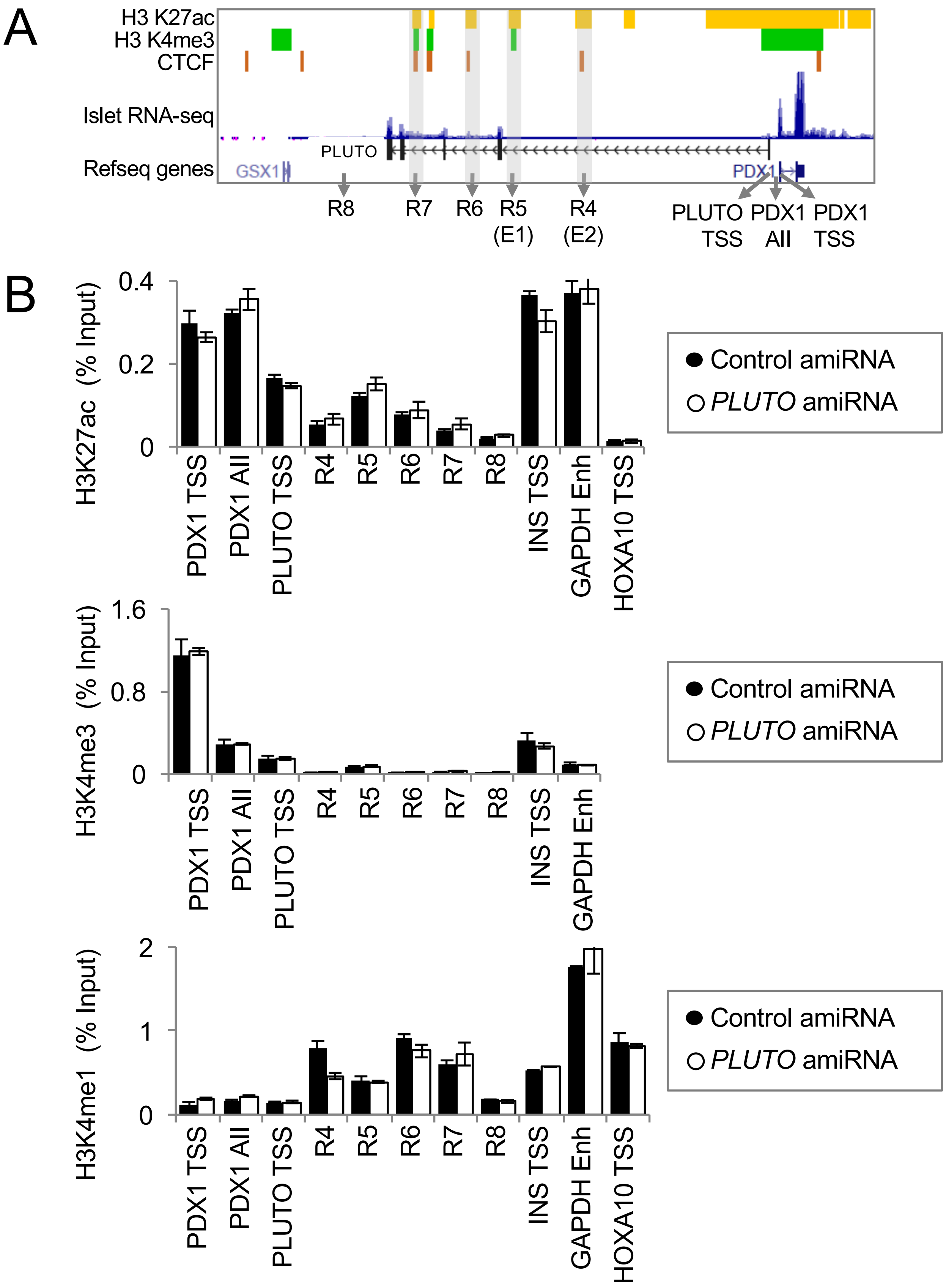

Figure S7. Effects of *PLUTO* on chromatin modification landscape of *PDX1* enhancers
(A) Schematic of *PDX1*/*PLUTO* locus displaying the position of the primer sets used for this study relative to the indicated human islet chromatin and transcript enrichments. (B) Graphs represent chromatin immunoprecipitation experiments with antibodies against H3K27 acetylation (n=5), H4K4 tri-methylation (n=3) and H3K4 mono-methylation (n=1) in EndoC-βH1 cells 80 hours post-transduction with lentiviral vectors carrying control or *PLUTO* amiRNA sequences. Values are expressed as percentage of input DNA, error bars denote ±SEM.

Supplemental Tables

Table S1. Features of human lncRNAs selected for functional analysis, related to Figures 1, S1, 6, S6.
Table S1a. Features of human and mouse beta cell lncRNAs analyzed with amiRNA knockdowns.
Table S1b. Isoforms of PLUTO (HILNC71) sequenced from 3'RACE.

Table S2. Co-expression network analysis, related to Figures 5, S5.
Table S2.1 Network modules resulting from weighted gene co-expression analysis (WCGNA) of 64 human islet RNA-seq samples
Table S2.2 Assignment of islet expressed genes to network modules resulting from weighted gene co-expression analysis (WCGNA) of 64 human islet RNA-seq samples.
Table S2.3 Assignment of lncRNAs to network modules resulting from weighted gene co-expression analysis

Table S3. List of human beta-cell lncRNAs, related to Figures S5.
Table S3a. List of de-novo annotated human beta-cell lncRNAs (bed12 format, hg19)
Table S3b. Complementary list of previously annotated lncRNAs expressed in beta cells (bed12 format, hg19)

Table S4. Sequences of amiRNAs and oligonucleotides, related to experimental procedures.
Table S4a. Targeting sequences for miR-RNAi based knockdown.
Table S4b. Primer sequences for semi-quantitative real time reverse transcription PCR used in this study.
Table S4c. Taqman probe sequences for quantitative real time reverse transcription PCR reactions used in Figures 6 and 7.
Table S4d. Primer Sequences for 3C experiments with PDX1 TSS as viewpoint.
Table S4e. Primer Sequences for chromatin immunoprecipitation experiments used in Figure S7.
Table S4f. gRNA sequences for CRISPRi used in Figure 6.
Table S4g. LNA GapmeR sequences from Exiqon used in Figure S2.

Table S5. Gene sets used for integrative analysis, related to experimental procedures.

Table S6. Human Islet LncRNAs that are highly co-expressed with a neighbouring coding gene, related to Figure S1.

Table S7. Differential expression analysis of human islet lncRNAs in islets from donors with T2D and IGT, related to Figures 6 and S6.
Table S7a Differential expression analysis of human islet lncRNAs in control vs T2D islets (DE-seq).
Table S7b Differential expression analysis of human islet LncRNAs in control vs impaired glucose tolerance (IGT) islets (DE-seq).

Supplemental experimental procedures

Human islets
Human islet experiments were performed following ethically approved protocols from Hospital Clinic de Barcelona, Geneva University Hospitals, University of Lille and Milano San Raffaele Hospital. Islets were isolated from multiorgan donors after informed consent from family members. Detailed information on a panel of human islets used for RNA-seq are provided elsewhere (Moran et al., 2012)(Moran et al, in preparation). Briefly, pancreatic islets were isolated and purified using established isolation procedures (Bucher et al., 2005; Kerr-Conte et al., 2010), and cultured prior to analysis as described (Moran et al., 2012). Islet purity was assessed by (a) dithizone staining using an aliquot of islets immediately prior to harvest, and (b) qPCR analysis of duct, acinar, and β-cell mar ers (*SOX9*, *CPA1*, *MIST1*, *PAX6*, and *INS*). Only samples showing >80% dithizone purity and marginal exocrine contaminant mRNAs were further processed.

Mouse islets
Mouse experiments were conducted following procedures approved by the Ethical Committee of Animal Experimentation of Hospital Clinic de Barcelona. Mouse islets were isolated from 10-14 week-old C57BL/6J mice (Parrizas et al., 2001). After isolation islets were incubated in 4 mM glucose RPMI 1640 medium, supplemented with 10% fetal calf serum, 100 U/ml penicillin, and 100 U/ml streptomycin for 48 hours. At 2, 4, 8 and 24 hours prior to 72 hours, the media was supplemented with glucose to achieve a final concentration of 11 mM. All islets were then harvested for RNA extraction at 72 hours. The experiment was repeated 3 times.

RNA-Seq
RNA-seq was performed with TruSeq Stranded Total RNA libraries (Illumina) generated from human pancreatic islet samples and used to generate paired-end 100 nucleotide reads.

Reverse transcription PCR analysis
RNA was extracted using Tripure reagent (Roche), RNA quality was ascertained with a 2100 Agilent Bioanalyzer before microarray analysis and RNA-sequencing (RIN>8). Prior to reverse transcription, RNA was treated with DNAseI (Sigma) for 20 minutes at room temperature. cDNA synthesis was carried out using Superscript III (Invitrogen) and real-time PCR was performed using SYBR Green detection with the ABI 7300 Real Time PCR system (van Arensbergen et al., 2010). For Figure 7B, mRNA and intronic RNA levels were measured using TaqMan assays. *TBP* or *Tbp* mRNAs were used to normalize the expression of all samples. *HPRT, ACTB* or *Hprt* mRNAs were also used as a second normalization control for most experiments, obtaining similar results (data not shown). Primers are provided in **Table S4**. For qPCR experiments bars represent average mean expression (±SEM) and p-values denote Student´s t-test calculated with GraphPad Prism software.

Cell culture
MIN6 cells were maintained in DMEM (Lonza) supplemented with 15% fetal calf serum, 100 U/ml penicillin, 100 U/ml streptomycin and 46 nM 2-Mercaptoethanol at 37°C in a humidified chamber with 5% CO_2**.** EndoC-βH1 and EndoC-βH3 cells were maintained as previously described (Ravassard et al., 2011; Scharfmann et al., 2014). EndoC-βH3 were induced to excise the T-antigen over a course of three weeks with six evenly spaced 4-Hydroxy tamoxifen (4-OHT, 1μM final) treatments. This procedure stops the cell cycle after 10-12 days. Cells were harvested between day 21 and 24. For mRNA stability experiments EndoC-βH1 cells were treated with Actinomycin D at 5 μg/ml.

Nuclear fractionation
Nuclear fractionation was done as previously described in the presence of RNAse inhibitors (Dart et al., 2004). RNA was then extracted from the fractionated and whole cell lysates using TriPure reagent.

Gene knockdown experiments using amiRNAs
Five non-targeting and four amiRNAs targeting each of 5 TF and 25 lncRNAs were designed using BLOCK-IT software (Invitrogen) and cloned into pTRIP-CMV gateway vectors as described (Moran et al., 2012) (see **Table S4** for amiRNA hairpin sequences). These were then used to produce lentiviruses, which were transduced into the EndoC-βH1/3 cells as described (Castaing et al., 2005; Scharfmann et al., 2014). The cells were harvested at 80 hours, and RNA levels were assessed using qPCR. Only amiRNAs that produced consistent inhibition of target RNAs to at least 50% in at least two consecutive experiments were considered further. Cells transduced with five different non-targeting control

amiRNAs and non-transduced cells were analyzed in parallel. Each amiRNA was transduced in duplicate in independent plates. For knockdown experiments in human islets, islets were dissociated with trypsin (0.05%), washed with PBS and transduced with lentivirus (as described for EndoC-βH1 cells), and plated onto poly-lysine coated wells with DMEM supplemented with 10% fetal calf serum, 100 U/ml penicillin and 100 U/ml streptomycin. The efficiency of transduction was judged to be >95% based on GFP expression. We found that expression of *PDX1*, *PAX6, IAPP, TXNIP* and *DDIT3* (three beta cell and two cellular stress markers) were similar in intact and dispersed islets after 80 hrs of culture.

Locked nucleic acid (LNA) and CRISPR interference (CRISPRi) experiments
Two independent LNA Gapmers targeting *HI-LNC12*, *HI-LNC80*, *HI-LNC78, HI-LNC71 (PLUTO) and PDX1* were obtained from Exiqon (sequences in **Table S4**). For CRISPRi experiments, the pSpCas9n(BB)-2A-GFP vector (a gift from Feng Zhang, Addgene plasmid # 48140) was modified into pSp-dCas9-2A-GFP by introducing the H840A mutation in Cas9 to obtain the catalytically inactive Cas9 (dCas9). Two separate CRISPRi guide RNAs were designed to target *PLUTO* exon 1, and cloned into pSp-dCas9-2A-GFP (sequences in **Table S4**) (Qi et al 2013, Gilbert et al 2014). Control CRISPRi guide RNAs were designed targeting intergenic regions. LNA gapmers (50 pmol) or CRISPRi vectors (6 μg) were nucleofected (program D-023) into 1-2 million EndoC-βH3 cells using Amaxa Cell Line Nucleofector Kit V (Lonza) and plated 80 hours prior to harvest.

Glucose stimulated insulin secretion (GSIS)
GSIS for **Figure 3** was carried out as described (Benazra et al., 2015). Secreted and total insulin was first normalized to cell number and then expressed as a fraction of levels at 2 mM control cells. For GSIS experiments in **Figure S3** (using LNA gapmers), tamoxifen treated EndoC-βH3 cells were subjected to nucleofection and plated 80 hours prior to GSIS. KREB´s buffer (115 mM NaCl, 24 mM $NaHCO_3$, 5 mM KCl, 1 mM $MgCl_2$, 1 mM $CaCl_2$,10 mM HEPES pH 7.5, 0.2 % BSA) was prepared freshly and equilibrated for 90 minutes in a humidified chamber with 5% CO_2. Cells were washed five times in warm PBS buffer and incubated with KREB´s buffer with 0.5 mM glucose for two hours. Cells were then incubated for 30 minutes with KREB´s buffer containing 0.5 mM, 6 mM or 16 mM glucose and 500 μM IBMX (Sigma). Supernatant containing secreted insulin was harvested, centrifuged (700g for 10 minutes at 4^0C) and quantified by ELISA (Mercodia, 10-1113-01). Attached cells were harvested with lysis buffer (50 mM Tris-HCl, pH8, 100 mM EDTA, 100 mM NaCl, 1% SDS, Roche protease inhibitor cocktail) and insulin was quantified by ELISA. Secreted insulin was normalized to total insulin content and expressed as fold enrichment over 0.5 mM control values. Bars represent an average of four independent experiments, and two independent control or targeting LNA mediated knockdown samples. P-values denote Student´s t-test calculated with GraphPad Prism software.

Immunoblotting
Equal amounts of protein from cell lysates were run on 10% bis-polyacrylimide gels and transferred to a PVDF membrane (0.45 μM). After blocking with 5% milk proteins in TBS - 0.02% Tween20 for one hour, the membrane was incubated overnight at 4^0 C with anti-PDX1 (Abcam, ab47383, 1:10,000) in the same buffer. Primary antibodies were detected by HRP-conjugated secondary antibodies (DAKO). Bands were detected and quantified using LAS4000 and ImageQuant software. Membrane was then stripped for reprobing with TBP (Santa Cruz, sc-273, 1:1000) followed by anti-Histone3 (Abcam, ab1791, 1:1000). PDX1 levels were then normalized by an average of TBP and H3.

Chromatin conformation capture
3C experiments were performed essentially as described (Pasquali et al., 2014; Tena et al., 2011). In brief, ~3×10^6 cells formaldehyde fixed EndoC-βH1 cells were transduced with control or targeting amiRNAs and lysed with lysis buffer (10 mM Tris-HCl pH 8, 10 mM NaCl, 0.3% IGEPAL-CA-630 (Sigma), 0.3% TritonX-100 (Sigma), 1X protease inhibitor (Complete, Roche)). Nuclei were digested with DpnII (NEB) and ligated with T4 DNA ligase (NEB). Primers were designed to amplify indicated ligated regions (**Table S4**), and were quantified by real-time PCR. Values were normalized to an intronic region of *PDX1* gene that does not contain a DpnII cut site, although similar results were obtained with or without normalization. The results were compiled from six independent experiments. Results were analyzed with Student's t-test for significance. 4C-Seq on ENDOC-βH1 cells (**Figure 7C**) was performed and analysed as previously described (Pasquali et al., 2014).

Chromatin immunoprecipitation
ChIP was performed with 1 million EndoC-βH1 cells/sample as described using the same antibodies and protocol as previously described (Pasquali et al., 2014).

***De novo* transcript assembly for islet lncRNAs**
We aligned ~5 billion stranded paired-end RNA-seq reads from 41 human islet samples to the human genome (hg19) as described (Moran et al., 2012). The reads falling into the top 143 most expressed genes were excluded. Remaining fragments were assembled into transcripts using cufflinks (Parameters: --min-isoform-fraction 0.01 --pre-mrna-fraction 0.10 --max-bundle-frags 5000000). Unannotated transcripts were extracted and retained if the following criteria were satisfied (i) presence of more than one exon in the transcript, (ii) presence of H3K4me3 enrichment (defined as described in Moran et al., 2012) in a region that is consistent with the transcriptional promoter (+1kb to -0.5 kb from the transcript 5' end), (iii) RNA Coding Potential Assessment Tool (CPAT) score < 0.364 (following recommendation for human genome sequences by (Wang et al., 2013)). For lncRNA genes with multiple isoforms, the isoform with the longest exon number was kept, or the isoform with the longest exon length in the case of multiple isoforms with the same number of exons. For 18 cufflinks-generated transcripts that did not fulfill the three above-mentioned criteria for lncRNAs, but there was a previously annotated non-coding Ensembl transcript of the same gene that fulfilled those same criteria, the latter lncRNA gene annotation was retained. We also included lncRNA genes defined by (Moran et al., 2012) that were not selected with the abovementioned annotation. Any lncRNA overlapping a coding exon on the same strand was removed from the list. Finally, the lncRNAs were filtered for expression of >0.05 FPKM in FACS purified beta cells and an acinar to beta cell expression ratio < 3, using FACS purified beta and acinar RNA-Seq data processed as described in (Moran et al., 2012). This resulted in a total number of 2226 de novo annotated lncRNA genes (**Table S3**).

We further generated a second list of 2112 previously annotated lncRNA genes, which contained Ensembl transcripts annotated by Havana as "lincRNA" or "antisense" and contained H3K4me3 enrichment in the 5' end, were expressed >0.05 FPKM in FACS purified beta cells, showed an acinar to beta cell expression ratio < 3, and had <10% overlap with any transcript from the list of 2226 de novo annotated lncRNAs (**Table S3**). This list included previously annotated lncRNAs that were not contained in the list of 2226 de novo annotated lncRNAs because they had higher CPAT scores than our thresholds, were monoexonic, overlapped a protein-coding gene, or were represented by transcripts with only minimal overlap. LncRNAs annotated in this study are available to download as a track hub on a UCSC genome browser (GRCh37/hg19) session by selecting "track hubs", and selecting "Human Islet lncRNAs". Alternatively the track hub can be directly visualized in the UCSC Genome Browser.

Differential expression analysis of IGT and T2D islets
Data was downloaded from (Fadista et al., 2014). The samples were aligned using STAR aligner version 2.3.0 with hg19 genome in which common SNPs (Global Minor Allele Frequency>1%) from the dbSNP database 142 were masked (Dobin et al., 2013; Sherry et al., 2001). A maximum mismatch of 10 nucleotides was used, and non-uniquely aligned reads were removed. Quantification of the raw read count was done using HTseq-Count version 0.6.1 with python 2.6.6 and Pysam version 0.8.3 (Anders et al., 2015). Differential expression analysis was done using DEseq2 version 1.10 on lncRNA genes (Table S3), using an adjusted p-value threshold of 0.05, and after removing one T2D outlier sample based on PCA (Love et al., 2014).

Co-expression network analysis
Sequence reads were aligned to the human reference genome hg38 with TopHat v.2.0.12, using Bowtie2(v.2.2.3) GENCODE(v21) and a customized lncRNA annotation were used as reference for mapping. Cufflinks (v2.2.1) was used to obtain FPKM values for gene and lncRNA expression(Harrow et al., 2012; Trapnell et al., 2012). Co-expression networks were built on mRNA expressions from 64 human islet samples using WGCNA(v2) package in R (Zhang and Horvath, 2005). Specifically, we first restricted the mRNAs into protein coding genes from HUGO Gene Nomenclature Committee (HGNC, downloaded on 5-19-2015), we then removed mRNAs that were not expressed in more than 50% samples. We inverse quantile normalized mRNA expressions, i.e., for each mRNA, we mapped its expression levels across samples into a vector generated from standard normal distribution $N(0,1)$ based on the rank of expressions across 64 samples). Finally, we corrected the batch effect using a linear regression model. The processed data were applied into WGCNA with power 6 to construct co-expression modules. The power of 6 was chosen using the scale-free topology criterion(Zhang and Horvath, 2005).

lncRNAs (**Table S3**) were filtered, normalized and batch effect corrected the same way as mRNAs. Only 2455 lncRNAs expressed in at least 50% of islet samples were used in this analysis. We assigned lncRNAs to the mRNA-derived co-expression network modules by calculating the correlation between lncRNA expression and module-gene (i.e., the first principal component of each module expression), and assigned the lncRNA to the module with the highest correlation with FDR less than 0.05.

Enrichment of lncRNAs in modules was calculated as observed over expected number of lncRNAs per module. Enrichment p-value was calculated for modules larger than 110 genes using the hypergeometric test in R.

Microarray analysis
RNA was hybridized onto HTA2.0 Affymetrix arrays. RMA normalisation was carried out using Expression Console software (Affymetrix). Gene based differential expression analysis was done using Transcriptome Analysis Console (TAC v1.0.24, Affymetrix), using RefSeq genes and a threshold ANOVA $p < 10^{-3}$ for calling differential gene expression. Raw data (.cel files) as well as analyzed microarray files (.chp) can be found at Gene Expression Omnibus (GEO, accession: GSE83619).

Cluster analysis
Genes that were dysregulated with ANOVA $p < 10^{-3}$ in at least one knockdown were included in the analysis. Genes were clustered based on Log2 of fold change of expression in control versus knockdown samples using Cluster3(v1.52) software with Euclidean Distance and Average Linkage analysis (de Hoon et al., 2004). Similar results were obtained using Centroid linkage (data not shown). Clustering was visualized using Treeview (v1.1.6) software.

Correlation analysis
For the heatmaps shown in **Figure 3A**, we selected all genes that are significantly deregulated ($p<10^{-3}$) in each pairwise comparison, and calculated Pearson´s r values based on correlation of fold change of expression for each gene upon knockdown of both lncRNA or TF. To assess correlation of transcriptional changes in pairwise comparisons of knockdowns (**Figure 3C**), we analyzed the top 100 targets from each knockdown and calculated Pearson's correlation r values for fold change (Log2) of expression. **Figure S3** displays p-values for Pearson´s correlation for the top 250 dysregulated genes in each TF or lncRNA knockdown in the vertical column of the heatmap. Top 250 dysregulated genes are defined as differentially expressed genes with the lowest 250 ANOVA p-values. All calculations were implemented using R software.

Gene set enrichment analysis (GSEA)
We used a previously defined subset of 694 enhancer cluster genes, which are active in human pancreatic islets and are linked to islet enhancer clusters that show high occupancy by islet TFs (PDX1, FOXA2, NKX2-2, NKX6.1, MAFB) (Pasquali et al., 2014). We further defined a set of 1139 genes that contained islet enhancers bound by at least four islet TFs within 25 Kb of the transcriptional start site, regardless of whether they show enhancer clustering, or defined genes that contained PDX1-bound enhancers in the same space, essentially as described (Pasquali et al., 2014)(**Table S5**). Genes associated with human islet enhancer clusters have been previously shown to include a major fraction of genes important for pancreatic islet differentiation or function (Pasquali et al., 2014). Using a previously islet specificity score (Moran et al., 2012) we have also generated a list of 700 genes that are expressed at least two standard deviations higher in human islets than the average expression value in a panel of 16 human tissues (**Table S5**).

Gene Set Enrichment Analysis (GSEA v2.1.0)(Subramanian et al., 2005) was used to calculate enrichments of gene sets amongst deregulated genes. For the analysis of each gene set, ten additional gene sets of the same size were generated based on bin-normalized expression levels in EndoC-βH1 cells. One target and 10 control gene sets were then run against RMA normalized expression data from knockdown and control samples on GSEA (SignalvsNoise, weighted). For the significance of gene set enrichment we display FWER p-values for normalized enrichment score, which provides a conservative estimation of the probability of a false discovery.

Enrichment analysis of functional annotations
For gene ontology analysis, DAVID (Huang da et al., 2009) was used with all Refseq genes as background. Functional clusters were derived from "Biological Process" category and were displayed with their respective Enrichment score, which provides a p-value for each functional cluster (negative Log10).

Supplemental references

Anders, S., Pyl, P.T., and Huber, W. (2015). HTSeq--a Python framework to work with high-throughput sequencing data. Bioinformatics *31*, 166-169.

Benazra, M., Lecomte, M.J., Colace, C., Muller, A., Machado, C., Pechberty, S., Bricout-Neveu, E., Grenier-Godard, M., Solimena, M., Scharfmann, R., et al. (2015). A human beta cell line with drug inducible excision of immortalizing transgenes. Molecular metabolism *4*, 916-925.

Bucher, P., Mathe, Z., Morel, P., Bosco, D., Andres, A., Kurfuest, M., Friedrich, O., Raemsch-Guenther, N., Buhler, L.H., and Berney, T. (2005). Assessment of a novel two-component enzyme preparation for human islet isolation and transplantation. Transplantation *79*, 91-97.

Castaing, M., Guerci, A., Mallet, J., Czernichow, P., Ravassard, P., and Scharfmann, R. (2005). Efficient restricted gene expression in beta cells by lentivirus-mediated gene transfer into pancreatic stem/progenitor cells. Diabetologia *48*, 709-719.

Dart, D.A., Adams, K.E., Akerman, I., and Lakin, N.D. (2004). Recruitment of the cell cycle checkpoint kinase ATR to chromatin during S-phase. The Journal of biological chemistry *279*, 16433-16440.

de Hoon, M.J., Imoto, S., Nolan, J., and Miyano, S. (2004). Open source clustering software. Bioinformatics *20*, 1453-1454.

Dobin, A., Davis, C.A., Schlesinger, F., Drenkow, J., Zaleski, C., Jha, S., Batut, P., Chaisson, M., and Gingeras, T.R. (2013). STAR: ultrafast universal RNA-seq aligner. Bioinformatics *29*, 15-21.

Fadista, J., Vikman, P., Laakso, E.O., Mollet, I.G., Esguerra, J.L., Taneera, J., Storm, P., Osmark, P., Ladenvall, C., Prasad, R.B., et al. (2014). Global genomic and transcriptomic analysis of human pancreatic islets reveals novel genes influencing glucose metabolism. Proceedings of the National Academy of Sciences of the United States of America *111*, 13924-13929.

Harrow, J., Frankish, A., Gonzalez, J.M., Tapanari, E., Diekhans, M., Kokocinski, F., Aken, B.L., Barrell, D., Zadissa, A., Searle, S., et al. (2012). GENCODE: the reference human genome annotation for The ENCODE Project. Genome research *22*, 1760-1774.

Huang da, W., Sherman, B.T., and Lempicki, R.A. (2009). Bioinformatics enrichment tools: paths toward the comprehensive functional analysis of large gene lists. Nucleic acids research *37*, 1-13.

Kerr-Conte, J., Vandewalle, B., Moerman, E., Lukowiak, B., Gmyr, V., Arnalsteen, L., Caiazzo, R., Sterkers, A., Hubert, T., Vantyghem, M.C., et al. (2010). Upgrading pretransplant human islet culture technology requires human serum combined with media renewal. Transplantation *89*, 1154-1160.

Love, M.I., Huber, W., and Anders, S. (2014). Moderated estimation of fold change and dispersion for RNA-seq data with DESeq2. Genome biology *15*, 550.

Moran, I., Akerman, I., van de Bunt, M., Xie, R., Benazra, M., Nammo, T., Arnes, L., Nakic, N., Garcia-Hurtado, J., Rodriguez-Segui, S., et al. (2012). Human beta cell transcriptome analysis uncovers lncRNAs that are tissue-specific, dynamically regulated, and abnormally expressed in type 2 diabetes. Cell metabolism *16*, 435-448.

Parrizas, M., Maestro, M.A., Boj, S.F., Paniagua, A., Casamitjana, R., Gomis, R., Rivera, F., and Ferrer, J. (2001). Hepatic nuclear factor 1-alpha directs nucleosomal hyperacetylation to its tissue-specific transcriptional targets. Molecular and cellular biology *21*, 3234-3243.

Pasquali, L., Gaulton, K.J., Rodriguez-Segui, S.A., Mularoni, L., Miguel-Escalada, I., Akerman, I., Tena, J.J., Moran, I., Gomez-Marin, C., van de Bunt, M., et al. (2014). Pancreatic islet enhancer clusters enriched in type 2 diabetes risk-associated variants. Nature genetics *46*, 136-143.

Ravassard, P., Hazhouz, Y., Pechberty, S., Bricout-Neveu, E., Armanet, M., Czernichow, P., and Scharfmann, R. (2011). A genetically engineered human pancreatic beta cell line exhibiting glucose-inducible insulin secretion. The Journal of clinical investigation *121*, 3589-3597.

Scharfmann, R., Pechberty, S., Hazhouz, Y., von Bulow, M., Bricout-Neveu, E., Grenier-Godard, M., Guez, F., Rachdi, L., Lohmann, M., Czernichow, P., et al. (2014). Development of a conditionally immortalized human pancreatic beta cell line. The Journal of clinical investigation *124*, 2087-2098.

Sherry, S.T., Ward, M.H., Kholodov, M., Baker, J., Phan, L., Smigielski, E.M., and Sirotkin, K. (2001). dbSNP: the NCBI database of genetic variation. Nucleic acids research *29*, 308-311.

Subramanian, A., Tamayo, P., Mootha, V.K., Mukherjee, S., Ebert, B.L., Gillette, M.A., Paulovich, A., Pomeroy, S.L., Golub, T.R., Lander, E.S., et al. (2005). Gene set enrichment analysis: a knowledge-based approach for interpreting genome-wide expression profiles. Proceedings of the National Academy of Sciences of the United States of America *102*, 15545-15550.

Tena, J.J., Alonso, M.E., de la Calle-Mustienes, E., Splinter, E., de Laat, W., Manzanares, M., and Gomez-Skarmeta, J.L. (2011). An evolutionarily conserved three-dimensional structure in the vertebrate Irx clusters facilitates enhancer sharing and coregulation. Nature communications *2*, 310.

Trapnell, C., Roberts, A., Goff, L., Pertea, G., Kim, D., Kelley, D.R., Pimentel, H., Salzberg, S.L., Rinn, J.L., and Pachter, L. (2012). Differential gene and transcript expression analysis of RNA-seq experiments with TopHat and Cufflinks. Nature protocols *7*, 562-578.

van Arensbergen, J., Garcia-Hurtado, J., Moran, I., Maestro, M.A., Xu, X., Van de Casteele, M., Skoudy, A.L., Palassini, M., Heimberg, H., and Ferrer, J. (2010). Derepression of Polycomb targets during pancreatic organogenesis allows insulin-producing beta-cells to adopt a neural gene activity program. Genome research *20*, 722-732.

Wang, L., Park, H.J., Dasari, S., Wang, S., Kocher, J.P., and Li, W. (2013). CPAT: Coding-Potential Assessment Tool using an alignment-free logistic regression model. Nucleic acids research *41*, e74.

Zhang, B., and Horvath, S. (2005). A general framework for weighted gene co-expression network analysis. Statistical applications in genetics and molecular biology *4*, Article17.
