# Supplementary material for: Human Pancreatic β Cell lncRNAs Control Cell-Specific Regulatory Networks
Source: Cell Metab. 2017 Feb 7;25(2):400–11. doi: 10.1016/j.cmet.2016.11.016 (PMC5300904; doi:10.1016/j.cmet.2016.11.016)
Supplement: Document S2. Article plus Supplemental Information [file mmc9.pdf]

Article

Cell Metabolism

Human Pancreatic β Cell lncRNAs Control Cell-Specific Regulatory Networks

Graphical Abstract

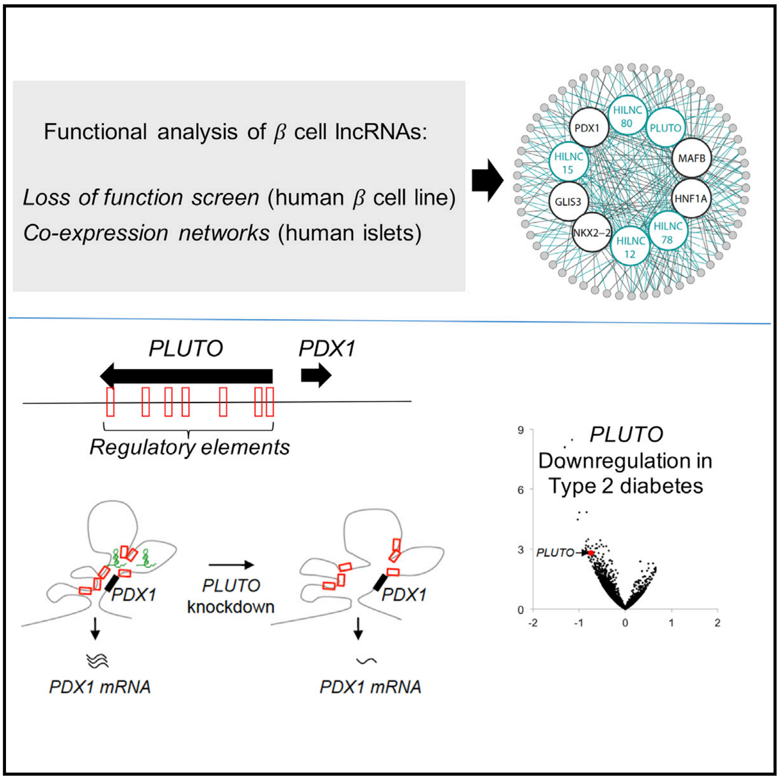

Authors

Ildem Akerman, Zhidong Tu, Anthony Beucher, ..., Eric Schadt, Philippe Ravassard, Jorge Ferrer

Correspondence

jferrerm@imperial.ac.uk

In Brief

Akerman et al. studied the function of human β cell lncRNAs with RNAi, CRISPRi, and co-expression networks. This revealed β cell lncRNAs and transcription factors that control common regulatory networks. One lncRNA, *PLUTO,* is downregulated in type 2 diabetes and controls *PDX1*, encoding a key β cell transcription factor.

Highlights

- A loss-of-function screen reveals functional β cell lncRNAs
- Cell-specific lncRNAs and transcription factors regulate common gene networks
- The lncRNA *PLUTO* influences interactions between an enhancer cluster and *PDX1*
- *PLUTO* and *PDX1* are deregulated in type 2 diabetes and impaired glucose tolerance

Accession Numbers

GSE83619

Akerman et al., 2017, Cell Metabolism *25*, 400–411
February 7, 2017 © 2016 Published by Elsevier Inc.
http://dx.doi.org/10.1016/j.cmet.2016.11.016

Cell Metabolism
Article

Human Pancreatic β Cell lncRNAs Control Cell-Specific Regulatory Networks

Ildem Akerman,[1,2,3] Zhidong Tu,[4] Anthony Beucher,[1] Delphine M.Y. Rolando,[1] Claire Sauty-Colace,[5] Marion Benazra,[5] Nikolina Nakic,[1] Jialiang Yang,[4] Huan Wang,[4] Lorenzo Pasquali,[3,6] Ignasi Moran,[1] Javier Garcia-Hurtado,[2,3] Natalia Castro,[2,3] Roser Gonzalez-Franco,[1] Andrew F. Stewart,[7] Caroline Bonner,[8] Lorenzo Piemonti,[9] Thierry Berney,[10] Leif Groop,[11] Julie Kerr-Conte,[8] Francois Pattou,[8] Carmen Argmann,[4] Eric Schadt,[4] Philippe Ravassard,[5] and Jorge Ferrer[1,2,3,12,*]
[1]Section of Epigenomics and Disease, Department of Medicine, Imperial College London, London W12 0NN, United Kingdom
[2]Genomic Programming of Beta Cells Laboratory, Institut d'Investigacions Biomediques August Pi I Sunyer (IDIBAPS), Barcelona 08036, Spain
[3]Centro de Investigación Biomédica en Red de Diabetes y Enfermedades Metabólicas Asociadas (CIBERDEM), Madrid 28029, Spain
[4]Department of Genetics and Genomic Science, Icahn School of Medicine at Mount Sinai, New York, NY 10029, USA
[5]Sorbonne Universités, UPMC Univ Paris 06, INSERM, CNRS, Institut du cerveau et de la moelle (ICM) – Hôpital Pitié-Salpêtrière, Boulevard de l'Hôpital, Paris 75013, France
[6]Germans Trias i Pujol University Hospital and Research Institute and Josep Carreras Leukaemia Research Institute, Badalona 08916, Spain
[7]Diabetes, Obesity, and Metabolism Institute, Icahn School of Medicine at Mount Sinai, New York, NY 10029, USA
[8]European Genomic Institute for Diabetes, INSERM UMR 1190, Lille 59800, France
[9]Diabetes Research Institute (HSR-DRI), San Raffaele Scientific Institute, Milano 20132, Italy
[10]Cell Isolation and Transplantation Center, University of Geneva, 1211 Geneva 4, Switzerland
[11]Department of Clinical Sciences, Lund University Diabetes Centre, Lund University, Lund 20502, Sweden
[12]Lead Contact
*Correspondence: jferrerm@imperial.ac.uk
http://dx.doi.org/10.1016/j.cmet.2016.11.016

SUMMARY

Recent studies have uncovered thousands of long non-coding RNAs (lncRNAs) in human pancreatic β cells. β cell lncRNAs are often cell type specific and exhibit dynamic regulation during differentiation or upon changing glucose concentrations. Although these features hint at a role of lncRNAs in β cell gene regulation and diabetes, the function of β cell lncRNAs remains largely unknown. In this study, we investigated the function of β cell-specific lncRNAs and transcription factors using transcript knockdowns and co-expression network analysis. This revealed lncRNAs that function in concert with transcription factors to regulate β cell-specific transcriptional networks. We further demonstrate that the lncRNA *PLUTO* affects local 3D chromatin structure and transcription of *PDX1*, encoding a key β cell transcription factor, and that both *PLUTO* and *PDX1* are downregulated in islets from donors with type 2 diabetes or impaired glucose tolerance. These results implicate lncRNAs in the regulation of β cell-specific transcription factor networks.

INTRODUCTION

Transcriptome surveys have uncovered tens of thousands of mammalian transcripts longer than 200 nucleotides that have low protein-coding potential (Carninci et al., 2005; Derrien et al., 2012; Guttman et al., 2009). A small fraction of these long non-coding RNAs (lncRNAs) have been shown to control gene expression by modulating chromosomal structure, transcription, splicing, mRNA transport, stability, or translation (Carrieri et al., 2012; Chen and Carmichael, 2009; Gong and Maquat, 2011; Lai et al., 2013; Luco and Misteli, 2011; Willingham et al., 2005; Yao et al., 2010). Specific lncRNAs have thus been implicated in various key processes, including random X chromosome inactivation, imprinting, the cell cycle, organogenesis, differentiation, pluripotency, and cancer progression (Guttman et al., 2011; Huarte et al., 2010; Hung et al., 2011; Klattenhoff et al., 2013; Kretz et al., 2013; Penny et al., 1996; Schmitt and Chang, 2013; Sleutels et al., 2002; Ulitsky et al., 2011). Despite these wide-ranging biological roles, the fraction of lncRNAs that is genuinely functional and the true impact of lncRNAs in human biology and disease remain poorly understood.

Pancreatic β cells regulate glucose homeostasis by secreting insulin and play a central role in the pathogenesis of major forms of diabetes mellitus. Recently, more than 1,100 lncRNAs were identified in human pancreatic islets and purified β cells (Morán et al., 2012) as well as in mouse pancreatic islet cells (Benner et al., 2014; Ku et al., 2012; Morán et al., 2012). A large fraction of human β cell lncRNAs are cell-specific, and several are known to be activated during β cell differentiation (Morán et al., 2012). This cellular specificity has also been noted for lncRNAs in other cell types (Cabili et al., 2011; Derrien et al., 2012) and points to the possibility that lncRNAs may regulate genetic programs important for lineage-specific differentiation or specialized cellular functions. Further, several β cell lncRNAs were shown to be regulated by extracellular glucose concentrations, suggesting a potential role of lncRNAs in the functional adaptation of β cells to increased insulin secretory demands (Morán et al.,

 © 2016 The Authors. Published by Elsevier Inc.
This is an open access article under the CC BY license (http://creativecommons.org/licenses/by/4.0/).

2012). Some islet lncRNAs map to loci that contain polygenic or Mendelian defects associated with human diabetes, whereas selected lncRNAs show deregulation in islets from organ donors with human type 2 diabetes (T2D) (Fadista et al., 2014; Morán et al., 2012). Collectively, these properties define a newly identified class of candidate regulators of β cell differentiation and function, with potential implications for human diabetes mellitus. However, the true relevance of β cell lncRNAs depends on whether they elicit a physiological function in human β cells, which remains to be addressed systematically.

In the current study, we have focused on a set of lncRNAs that show restricted expression in human pancreatic β cells and have tested the hypothesis that they regulate β cell gene expression. Our studies have uncovered a regulatory network in which lineage-specific lncRNAs and transcription factors (TFs) control common genes. Furthermore, we show that lncRNAs frequently regulate genes associated with clusters of islet enhancers, which have previously been shown to be the primary functional targets of islet-specific TFs. We performed a detailed analysis of a specific lncRNA named *PLUTO*, which controls *PDX1*, a master regulator of pancreas development and β cell differentiation and, thereby, modulates the *PDX1*-dependent transcriptional program. Finally, we show that *PLUTO* and *PDX1* are downregulated in islets from organ donors with type 2 diabetes or impaired glucose tolerance, suggesting a potential role in human diabetes.

RESULTS

Human β Cell lncRNA Knockdowns Cause Profound Transcriptional Phenotypes

To directly test the regulatory function of pancreatic β cell lncRNAs, we carried out loss-of-function experiments in a glucose-responsive human islet β cell line, EndoC-βH1 (Ravassard et al., 2011). We chose a human model because only some human lncRNAs are evolutionary conserved (Derrien et al., 2012; Morán et al., 2012; Okazaki et al., 2002; Pang et al., 2006), and we perturbed the function of lncRNAs through RNAi-based transcript knockdowns rather than genomic deletions because deletions could potentially disrupt *cis*-regulatory elements. We thus designed lentiviral vectors that contain RNA polymerase II-transcribed artificial microRNAs (hereafter referred to as amiRNA) with perfect homology to the target sequence to elicit target cleavage. The amiRNAs contain an artificial stem sequence targeting our lncRNA of choice as well as flanking and loop sequences from an endogenous miRNA to allow their processing as pre-miRNA by the RNAi pathway (Figure S1A). As a reference, we used the same strategy to knock down TFs that are well known to regulate gene expression in pancreatic islets as well as five different non-targeting amiRNA sequences as controls.

The lncRNAs selected for knockdown were derived from a short list of 25 lncRNAs that showed (1) a markedly enriched expression in human islets and fluorescence-activated cell sorting (FACS)-purified β cells relative to the exocrine pancreas and a panel of non-pancreatic tissues, (2) expression in the EndoC-βH1 β cell line, and (3) a chromatin profile in human islets that was consistent with an active promoter (Figures S1C and S1D). Of these 25 lncRNAs, 12 were shortlisted because they were near a protein-coding gene that has an important function in β cells. The lncRNAs had variable subcellular enrichment patterns (Figure S1B), and eight of the 12 lncRNAs had detectable transcripts in orthologous or syntenic mouse regions (Table S1; Morán et al., 2012). We then screened four amiRNA sequences for each of the 12 lncRNAs and identified two efficient (>50% knockdown) amiRNAs for seven lncRNAs and one efficient amiRNA sequence for the other five lncRNAs (Figure S1E). Two efficient amiRNAs were also obtained for five essential islet TFs (*HNF1A*, *GLIS3*, *MAFB*, *NKX2.2*, and *PDX1).* We thus transduced EndoC-βH1 cells with lentiviruses expressing each amiRNA. This was done in duplicate or in triplicate for lncRNAs that only had one efficient amiRNA. 80 hr post-transduction, RNA was harvested and hybridized to oligonucleotide microarrays (Figure 1A). For each target gene, we combined expression data from all knockdowns and compared them to the control transductions with five different control amiRNAs to identify genes that were differentially expressed at a significance level of $p < 10^{-3}$ (ANOVA) (Figure 1B).

As expected, knockdown of islet TFs consistently produced transcriptional phenotypes (Figure 1B). Remarkably, knockdown of 9 of the 12 islet lncRNAs also caused transcriptional changes (Figure 1B; Figure S1F). A more detailed analysis showed that some of the lncRNAs that presented knockdown phenotypes had visible effects on a neighboring gene, suggesting a possible *cis*-regulatory mechanism, although other such lncRNAs did not appear to affect neighboring genes and may thus function through *trans*-regulatory mechanisms (Figure 1E; Figure S1G). These loss-of-function experiments with selected lncRNAs therefore suggested that lncRNAs can regulate the expression of pancreatic β cell genes.

Gene silencing using the RNAi pathway can theoretically lead to nonspecific gene deregulation. In our experimental model, a significant nonspecific result would occur when two unrelated amiRNAs elicited changes in a common set of genes that were not observed in the panel of control non-targeting amiRNAs. To assess the likelihood that two unrelated amiRNA sequences elicit such an effect, we studied the five sets of control (non-targeting) amiRNAs, compared all ten possible combinations of two versus three control amiRNAs, and determined the number of differentially expressed genes (Figure 1C). Likewise, for each TF or lncRNA that had two valid amiRNAs, we compared the two target-specific amiRNAs against all possible combinations of three control amiRNAs (Figure 1C). As seen in Figure 1D, control versus control comparisons generated a median of 16 (IQR = 15–22) differentially expressed genes, whereas all five TFs and six of the seven lncRNA knockdowns led to a significantly higher number of differentially expressed genes (Mann-Whitney test, $p < 10^{-4}$ for all lncRNA/TF versus control comparisons except *HI-LNC75*, p = 0.004, and *HI-LNC76*, p > 0.5). These results show that the observed phenotypes are unlikely to be caused by unspecific effects of amiRNAs and indicate that the sequence-specific inhibition of selected islet lncRNAs can result in transcriptional changes comparable in magnitude to the inhibition of well-established islet transcriptional regulators.

The primary function of β cells is to synthesize and secrete insulin in response to changes in glucose concentrations. Among the genes that showed functional dependence on lncRNAs, we identified numerous genes that are known to

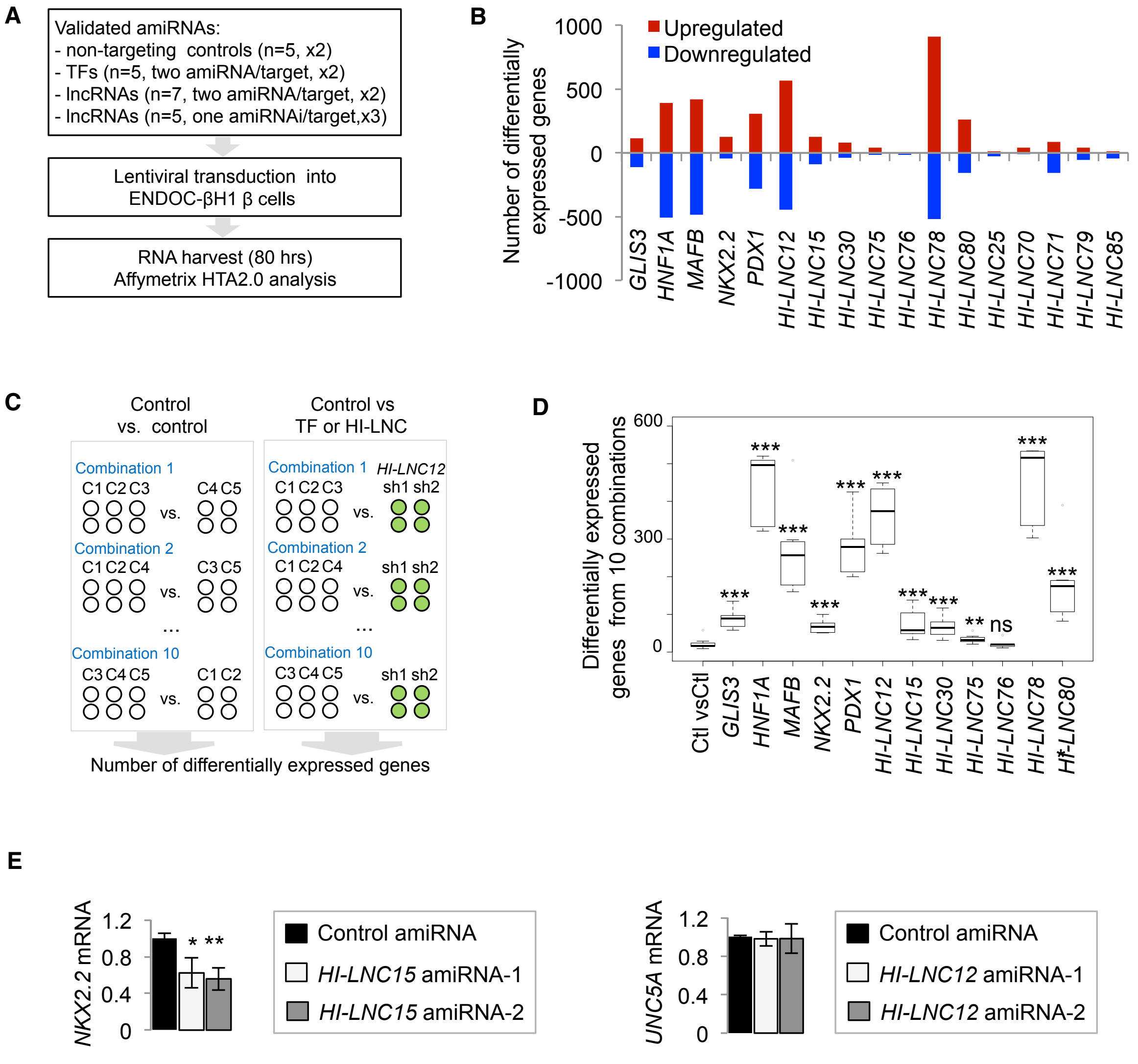

Figure 1. Knockdown of Selected β Cell lncRNAs Leads to Transcriptional Phenotypes

(A) Schematic of the experimental plan. Lentivirally encoded amiRNAs were validated and transduced in duplicate (×2) or triplicate (×3) into ENDOC-βH1 cells as indicated and then analyzed with oligonucleotide expression arrays.

(B) Differential gene expression analysis revealed genes that show significant up- or downregulation after knockdown of TFs or lncRNAs. For each TF or lncRNA, we combined all replicates transduced with the different target-specific amiRNAs and compared these with all replicates from five non-targeting controls. Differential expression was determined at $p < 10^{-3}$ (ANOVA).

(C) We compared gene expression data from all ten possible combinations of three versus two control non-targeting amiRNAs. Similarly, the two independent amiRNAs that target each TF or lncRNA were compared with all ten possible combinations of three control amiRNAs. For this analysis, we only considered the seven lncRNAs that were targeted by two independent amiRNAs.

(D) Control comparisons result in a low number of differentially regulated genes (average 15 genes), whereas most TF and lncRNA comparisons yield higher numbers of differentially regulated genes. ***$p < 10^{-4}$; **$p < 0.01$; ns, not significant compared with control comparisons; Mann-Whitney test.

(E) *HI-LNC15* regulates its neighboring gene, *NKX2.2*, whereas *HI-LNC12* knockdown (KD) does not affect its adjacent active gene, *UNC5A* (left). Further examples are shown in Figure S1G. RNAs were normalized to *TBP* mRNA and expressed relative to control amiRNAs; n = 3, error bars represent SEM; **$p < 0.01$, *$p < 0.05$ (Student's t test).

regulate transcription or secretion in β cells, including *RFX6*, *PDX1*, *CACNA1D*, *ATP2A3*, *ROBO1* and *2*, *PDE8A*, *ATP6AP1*, *KCNJ15*, *TRPM3*, *ERO1LB*, and *HADH* (Figure 2A; Anderson et al., 2011; Li et al., 2010; Louagie et al., 2008; Okamoto et al., 2012; Smith et al., 2010; Tian et al., 2012; Varadi and Rutter, 2002; Wagner et al., 2008; Yang et al., 2013; Zito et al., 2010).

We therefore measured insulin content and glucose-stimulated insulin secretion (GSIS) in T antigen-excised EndoC-βH3 cells after knocking down four lncRNAs that showed the strongest transcriptional phenotypes (*HI-LNC12*, *HI-LNC78*, *HI-LNC80*, and *HI-LNC71*). Congruent with the broad transcriptional phenotype, we observed reduced insulin content and, consequently,

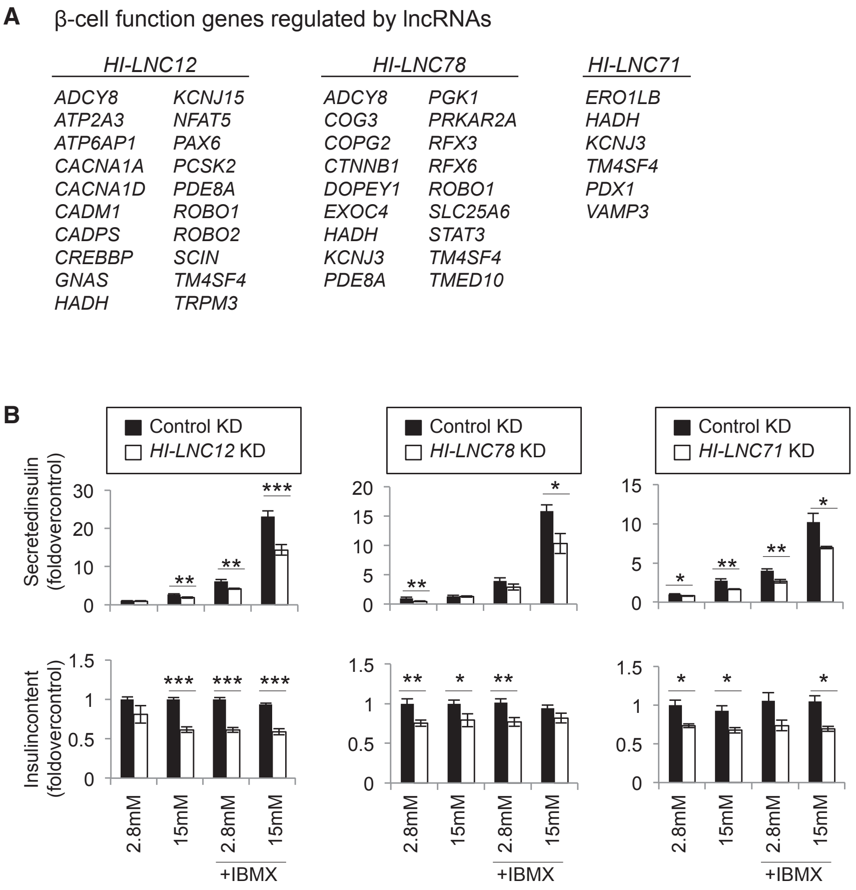

Figure 2. Knockdown of lncRNAs Impairs Insulin Secretion

(A) Examples of genes known to play a role in β cell function regulated by islet lncRNAs.

(B) Glucose-stimulated insulin secretion was tested on T antigen-excised EndoC-βH3 cells after transduction with amiRNAs targeting the indicated lncRNAs or controls. Secreted or total insulin content was normalized to the number of cells per well and expressed as fold change over control amiRNA treatment at 2.8 mM glucose. Each bar represents an average from two independent amiRNA vectors and 12 separate wells from two independent experiments. Error bars represent SEM; ***$p < 10^{-3}$, **$p < 0.01$, *$p < 0.05$ (Student's t test).

impaired glucose-stimulated insulin secretion for *HI-LNC12*, *HI-LNC78*, and *HI-LNC71* knockdowns (Figure 2B). For *HI-LNC78*, a glucose-regulated islet transcript (Morán et al., 2012) that is orthologous to mouse *Tunar* and zebrafish *megamind (linc-birc6)* lncRNAs (Ulitsky et al., 2011), there was a reduction in GSIS after correcting for the reduction in insulin content ($p = 0.002$) (Figure S2A). To further validate these effects, the same lncRNAs were downregulated using antisense locked nucleic acid (LNA GapmeRs, Exiqon) GapmeRs, which also led to impaired insulin secretion after knockdown of *HI-LNC12* and *HI-LNC78* (Figure S2B). Taken together, lncRNA knockdown studies identified lncRNAs that modulate gene expression and, consequently, insulin secretion in a human β cell line.

Human Islet lncRNAs and TFs Regulate Common Gene Expression Programs

To gain insight into the expression programs that are regulated by islet-specific lncRNAs and TFs, we compared their knockdown gene expression phenotypes. We first assessed changes in gene expression occurring after knockdown of the different islet TFs and found high Pearson correlation values for all pairwise comparisons ($r = 0.4–0.8$, $p < 10^{-27}$) (Figure 3A; Figure S3). This finding is consistent with the notion that islet-specific TFs often bind to common genomic targets and function in a combinatorial manner (Pasquali et al., 2014; Qiu et al., 2002; Wilson et al., 2003). Interestingly, the transcriptional changes that occurred after the inhibition of several lncRNAs significantly correlated with those observed following inhibition of TFs (Figure 3A; Figure S3; see also a cluster analysis of TF- and lncRNA-dependent changes in Figure 3B). Some pairwise comparisons that illustrate this finding include *HI-LNC78*-dependent gene expression changes, which correlated highly with *HNF1A*- and *MAFB*-dependent changes (Pearson's $r = 0.87$ and 0.89, respectively, $p < 10^{-71}$), and *HI-LNC15*-dependent changes, which correlated with those occurring after knockdown of *NKX2-2* ($r = 0.67$, $p = 10^{-32}$) (Figure 3C). The results from these gene knockdown experiments therefore indicate that selected islet-specific lncRNAs and TFs can regulate common gene expression programs.

Islet TFs and lncRNAs Co-regulate Genes Associated with Enhancer Clusters

Recent studies have revealed that islet TFs regulate cell-specific transcription by targeting clusters of enhancers and, in particular, clusters with enhancers that are bound by multiple islet TFs (Pasquali et al., 2014). Enhancer clusters share many features with regulatory domains that have otherwise been defined as "stretch enhancers" or "superenhancers" (Pasquali et al., 2014; Pott and Lieb, 2015). Given that knockdown of islet lncRNAs and TFs suggested that they regulate similar genes, we asked whether islet lncRNAs also regulate enhancer cluster-associated genes. As expected, gene set enrichment analysis (GSEA) showed that genes with islet-enriched expression, genes associated with enhancer clusters, or genes associated with enhancers that are bound by multiple TFs were downregulated after knockdown of all five TFs, whereas this was not observed for ten control sets of genes expressed at similar levels (Figure 4; Figures S4A and S4B). Likewise, genes associated with enhancer clusters and those showing islet-specific expression were also enriched among genes that were downregulated after knockdown of *HI-LNC12*, *15*, *30*, *78*, *80, 85*, and *71* (Figure 4; Figures S4A and S4B). These results therefore indicate that islet-specific TFs and lncRNAs often co-regulate genes that are associated with enhancer clusters.

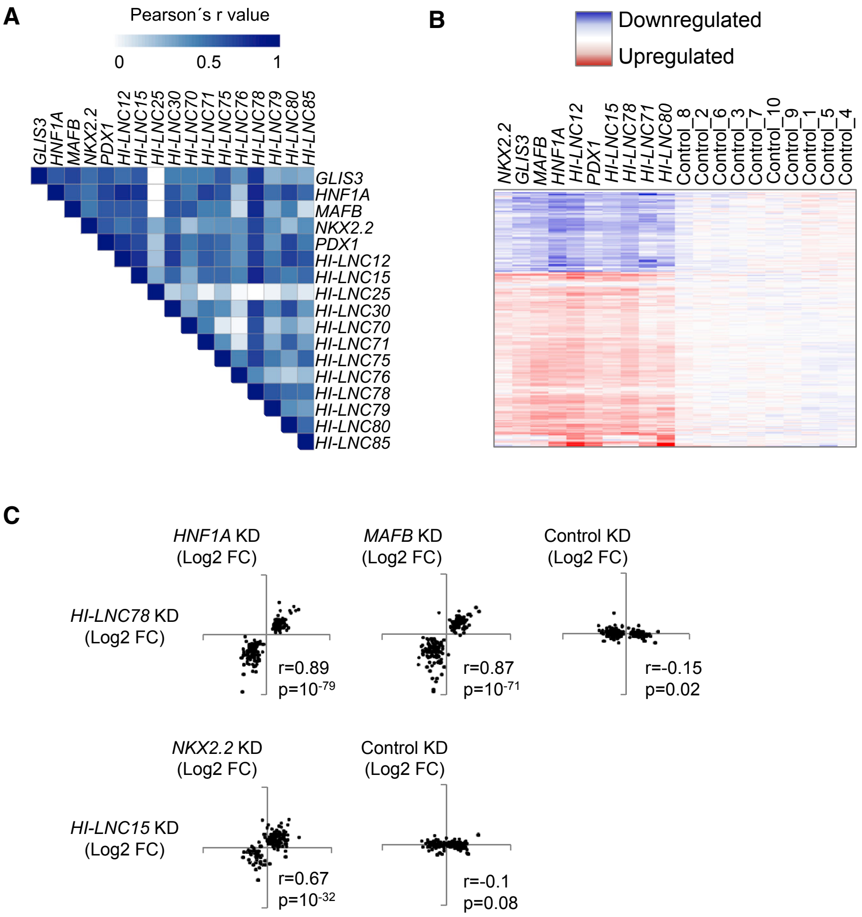

Figure 3. Human Islet TFs and lncRNAs Regulate Common Genes

(A) Heatmap displaying Pearson r values for all pairwise comparisons of fold changes in gene expression after knockdown of TFs and lncRNAs. Only genes significantly dysregulated at $p < 10^{-3}$ under at least one condition were included in the analysis.

(B) Unsupervised clustering analysis of fold change values after knockdown of five TFs and the five lncRNAs that displayed the strongest transcriptional changes. Only genes that were dysregulated at $p < 10^{-3}$ in at least one knockdown were selected. Blue represents downregulated and red represents upregulated genes. Controls represent control comparisons as described for Figure 1.

(C) Examples of highly correlated transcriptional phenotypes. The plots show fold change values (Log2) after knockdown of the indicated pairs of genes. Only the top 100 most regulated genes for any of the two knockdowns were plotted. Pearson's correlation (r) and p values are displayed.

β Cell lncRNAs and TFs Form Part of Islet-Specific Co-expression Networks

We next used an independent experimental approach to validate the observation that human β cell lncRNAs and TFs regulate common gene expression programs. This involved the analysis of gene modules that show co-expression across a panel of human islet RNA samples. Analogous approaches have been employed to reveal sets of genes that share functional relationships (Derry et al., 2010; Kim et al., 2001; Pandey et al., 2010; Segal et al., 2003; Stuart et al., 2003; Su et al., 2011). We implemented this analysis using weighted gene co-expression analysis (WGCNA) of RNA sequencing (RNA-seq) profiles from 64 human pancreatic islet samples. This identified 25 major gene modules containing more than 100 genes, named M1–M25, that showed highly significant co-expression across human islet samples (Figure 5A; Table S2). We next determined which co-expression modules contained islet lncRNAs. Rather than using our previously defined set of lncRNAs, this analysis was performed with a set of 2,373 β cell lncRNAs that was newly annotated using ~5 billion stranded RNA-seq reads pooled from 41 islet samples (Table S3; Figure S5A). β Cell lncRNAs were found to be enriched in seven pancreatic islet co-expression modules (M3, M7, M12, M13, M18, M20, and M21) (Figure 5B).

We next characterized the nature of these seven lncRNA-enriched co-expression modules. Five of these (M3, M7, M12, M18, and M20) were enriched in genes associated with pancreatic islet enhancer clusters (Figures 5A–5C, marked in blue). Two other modules (M13 and M21) were enriched for ubiquitously expressed genes involved in mRNA translation and metabolic pathways (Figure S5B). Among the modules enriched in lncRNAs and enhancer clusters, three (M3, M7, and M18) were also enriched in islet-specific TF genes (Figure 5D), and two of these modules (M3 and M7) contained nine of the 12 lncRNAs that had been knocked down in EndoC-βH1 cells. Module M3, the largest of the seven lncRNA-enriched modules, featured gene ontology (GO) terms associated with prototypical islet cell functions and contained several islet TFs and lncRNAs (Figure 5E). In keeping with these findings, we found numerous instances of islet lncRNAs and known cell-specific TFs that showed a tight correlation of gene expression levels across human islet samples (Figure 5F; Figure S5C). These findings thus indicated that β cell-specific lncRNAs, TFs, and genes associated with islet enhancer clusters form part of common expression programs.

Further analysis is consistent with the notion that lncRNAs play a functional role in driving gene expression variation in the lncRNA-enriched co-expression modules. First, the subset of lncRNAs that were shown to regulate an adjacent gene in knockdown studies also exhibited a particular high co-regulation with the adjacent gene across islet samples (Figure S1G). This observation was extended to define 292 lncRNAs that displayed a highly significant ($p < 10^{-7}$) correlation of expression with an adjacent protein-coding gene in the panel of human islet samples and are thus candidate *cis*-regulatory lncRNAs (Table S6). Second, we analyzed all genes that were significantly downregulated in EndoC-βH1 cells after knocking down *HI-LNC12*, *71*, *78*, and *80* and found that they were also enriched among genes in human islet modules M3, M7, and M18 but not in size-controlled modules (Figure S5D). In summary, co-expression analysis of native human islets corroborated the findings observed with

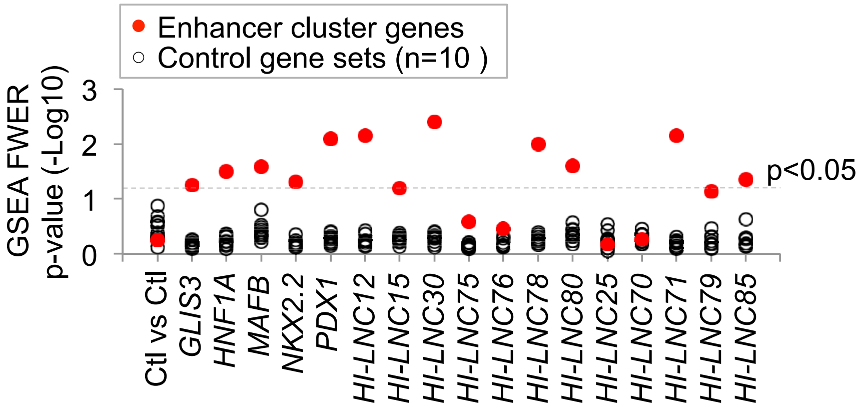

Figure 4. LncRNAs Regulate Enhancer Cluster Genes

GSEA showed that genes that were downregulated upon knockdown of either islet TFs or lncRNAs were enriched in a set of 694 genes that is associated with human islet enhancer clusters (red dots) but not in ten control gene sets (black dots) that were expressed at similar levels as enhancer cluster genes.

amiRNA-based perturbations in EndoC-βH1 cells and indicated that a group of islet lncRNAs and TFs form part of common transcriptional networks that target clusters of pancreatic islet enhancers (Figure 5G).

Deregulation of β Cell lncRNAs in Human T2D

The identification of functional lncRNAs led us to explore whether some lncRNAs are abnormally expressed in human T2D and might thus be relevant to the pathogenesis of this disease. We therefore analyzed our new set of 2,373 lncRNAs in a recently reported gene expression dataset that includes human islet samples from donors diagnosed with T2D or impaired glucose tolerance (IGT) (Fadista et al., 2014). Our results showed that, despite the fact that gene expression across human islet donors is highly variable, the expression of 15 and 100 lncRNAs was significantly altered in islets from T2D and IGT versus non-diabetic donors respectively (adjusted $p < 0.05$) (Figure S6A; see Table S7 for a complete list). This finding suggests a potential role of functional β cell lncRNAs in driving some of the β cell gene expression changes that are associated with T2D.

PLUTO Regulates *PDX1*, an Essential Transcriptional Regulator

To explore how β cell lncRNAs can regulate cell-specific transcriptional networks, we focused on *HI-LNC71*, a nuclearly enriched transcript (Figure S1B) that is transcribed from a promoter that is located ~3 kb upstream of *PDX1*, in an antisense orientation (Figure S6B). PDX1 is an essential transcriptional regulator of pancreas development and β cell function that has been implicated in genetic mechanisms underlying Mendelian and type 2 diabetes (Ahlgren et al., 1998; Jonsson et al., 1994; Offield et al., 1996; Stoffers et al., 1997). Based on this genomic location, we renamed *HI-LNC71 PLUTO*, for *PDX1* locus upstream transcript.

The potential importance of *PLUTO* was strengthened by the observation that *PLUTO* was among the most markedly downregulated lncRNAs in islets from T2D or IGT donors (adjusted p value = 0.07 and 0.005, respectively; Figure 6A; Figure S6B). Interestingly, PDX1 was also downregulated in islets from donors with T2D and IGT (Figure 6A).

PLUTO is a multi-isoform transcript that contains five major exons that span nearly 100 kb, encompassing a cluster of enhancers that make 3D contacts with the *PDX1* promoter in human islets and in EndoC-βH1 cells (Figure 6B; Figure S6A). This observation suggested that *PLUTO* could affect *cis* regulation of the *PDX1* gene.

To test whether *PLUTO* regulates *PDX1*, we first examined EndoC-βH1 cells after amiRNA-mediated knockdown of *PLUTO* RNA and found reduced *PDX1* mRNA and protein levels (Figure 6C). Similarly, knockdown of *PLUTO* RNA in dispersed primary human islet cells caused decreased *PDX1* mRNA (Figure 6D). To validate these experiments through a complementary approach, we used CRISPR interference (CRISPRi), which involves targeting guide RNAs (gRNAs) downstream of a gene's transcriptional initiation site to block its transcription. Two independent gRNAs that targeted a region downstream of the *PLUTO* initiation site efficiently reduced *PLUTO* RNA levels relative to non-targeting gRNAs, and, in both cases, this led to decreased *PDX1* mRNA expression (Figure 6E). Therefore, perturbing either *PLUTO* RNA levels or its transcription leads to the same inhibitory effect on *PDX1* mRNA.

The mouse *Pdx1* locus also has an islet lncRNA (*Pluto*) that shows only limited sequence homology with human *PLUTO*. *Pluto* is also transcribed from the opposite strand of *Pdx1* but is initiated from a promoter within the first intron of *Pdx1* and, like *PLUTO*, spans a broad regulatory domain upstream of *Pdx1* (Figure S6C). Knockdown of *Pluto* RNA in the mouse β cell line MIN6 also led to decreased *Pdx1* mRNA levels (Figure S6E). These experiments therefore indicated that *PLUTO* regulates *PDX1* mRNA in human β cell lines and primary islet cells, and an analogous effect was observed for the mouse lncRNA ortholog.

Consistent with this regulatory relationship, *PLUTO* and *PDX1* RNA levels are highly correlated across islet samples (Pearson's $r = 0.86$, $p = 10^{-15}$; Figure 6F), and knockdown of *PDX1* and *PLUTO* in EndoC-βH1 cells resulted in the deregulation of a shared set of genes (Figures 6G–6J). Furthermore, *Pluto* and *Pdx1* were found to be regulated with nearly identical dynamics in response to a shift in glucose concentration (4–11 mM) in mouse pancreatic islets (Figure S6D). *PLUTO* and *PDX1* therefore regulate a common program in pancreatic islets, and this is at least in part explained by the fact that *PLUTO* regulates *PDX1.*

PLUTO Regulates *PDX1* Transcription and Local 3D Chromatin Structure

To assess the mechanisms underlying the function of *PLUTO*, we first examined whether *PLUTO* controls the stability or transcription of *PDX1.* Transcriptional inhibition experiments using Actinomycin D showed no significant differences in the stability

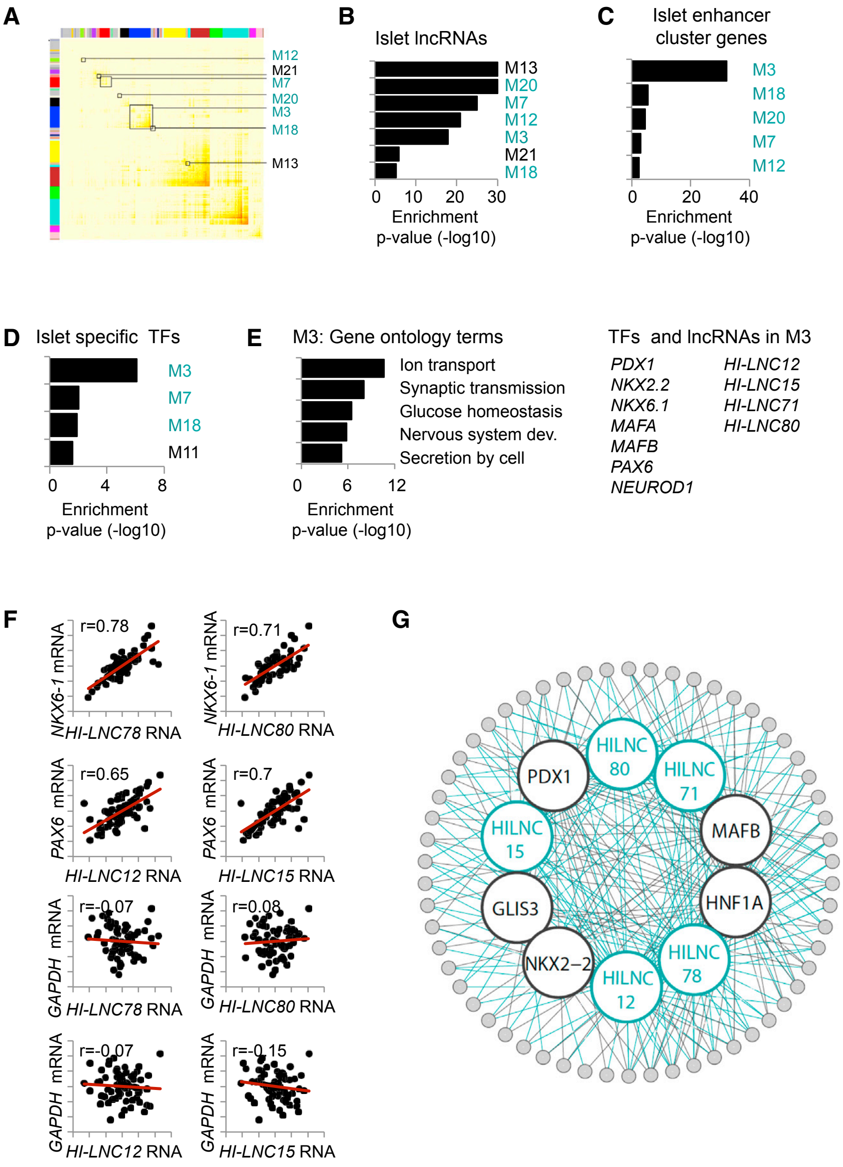

Figure 5. Islet-Specific Coding and Noncoding RNAs Form Shared Co-expression Modules

(A) Topological overlap matrix representing co-expression modules that were co-regulated across 64 human islet samples. Modules that were enriched in lncRNAs are marked with squares (hypergeometric test, $p < 10^{-2}$).

(B–D) Co-expression modules that showed enrichment in islet lncRNAs (B), islet enhancer cluster (EC)-associated genes (C), or a set of 94 islet-enriched TF genes (D). Five modules (M3, M7, M12, M18, and M20, marked in blue) out of seven modules that were enriched in lncRNAs were also enriched in ECs and TFs.

(E) Module M3 was enriched in typical islet-specific biological process annotations. Right: examples of islet TFs and lncRNAs in module M3.

(F) Correlation of the indicated lncRNAs and β cell-specific TF mRNAs across 64 islet samples. *GAPDH* is shown as a non-β cell reference. Pearson's correlation values are displayed in the top left corner. The axes show expression values normalized across 64 islet samples.

(G) Network diagram illustrating that TFs and lncRNAs often co-regulate the same genes, many of which are associated with enhancer clusters.

of *PDX1* mRNA upon *PLUTO* knockdown (Figure 7A). By contrast, intronic *PDX1* RNA was reduced upon *PLUTO* knockdown, suggesting that *PLUTO* regulates *PDX1* transcription (Figure 7B).

Because *PLUTO* spans an enhancer cluster, we hypothesized that it could regulate the chromatin state of active enhancers. We thus knocked down *PLUTO* in β cells and measured H3K27 acetylation as well as H3K4 mono- and tri-methylation levels at several enhancers within the cluster. Our results indicate no significant changes in these characteristic active chromatin marks (Figure S7).

We next determined whether *PLUTO* affects the 3D contacts between the enhancer cluster and the *PDX1* promoter. Examination of the *PDX1* locus using quantitative chromatin conformation capture (3C) assays revealed that two far upstream enhancers (Figure 7C) showed reduced contacts with the *PDX1* promoter after *PLUTO* knockdown (Figure 7D). These findings therefore show that *PLUTO* regulates the transcription of *PDX1*, a key pancreatic β cell transcriptional regulator, and that this is associated with its ability to promote contacts between the *PDX1* promoter and its enhancer cluster (Figure 7E).

DISCUSSION

In the current study, we have tested the hypothesis that lncRNAs play a role in cell-specific gene regulation in pancreatic β cells, a cell type that is central in the pathogenesis of human diabetes. We have thus carried out, for the first time, a systematic analysis of the function of a set of human β cell-specific lncRNAs. Our experiments revealed several examples of β cell lncRNAs in which sequence-specific perturbation causes transcriptional and functional phenotypes. We have further shown that β cell-specific lncRNAs and TFs regulate a common transcriptional network. Finally, we have demonstrated that β cell-specific lncRNAs directly or indirectly participate in the regulation of human enhancer clusters, which are the major functional targets of islet-specific transcription factors and key *cis*-regulatory determinants of islet cell transcriptional programs (Pasquali et al., 2014). Importantly, these conclusions are supported by concordant results from co-expression network analysis and loss of function experiments. These studies should be interpreted in light of previous evidence indicating that a significant fraction of lncRNAs show lineage-specific expression (Cabili et al., 2011; Derrien et al., 2012; Goff et al., 2015; Guttman et al., 2011; Iyer et al., 2015; Morán et al., 2012; Pauli et al., 2012). Our study extends

CellPress

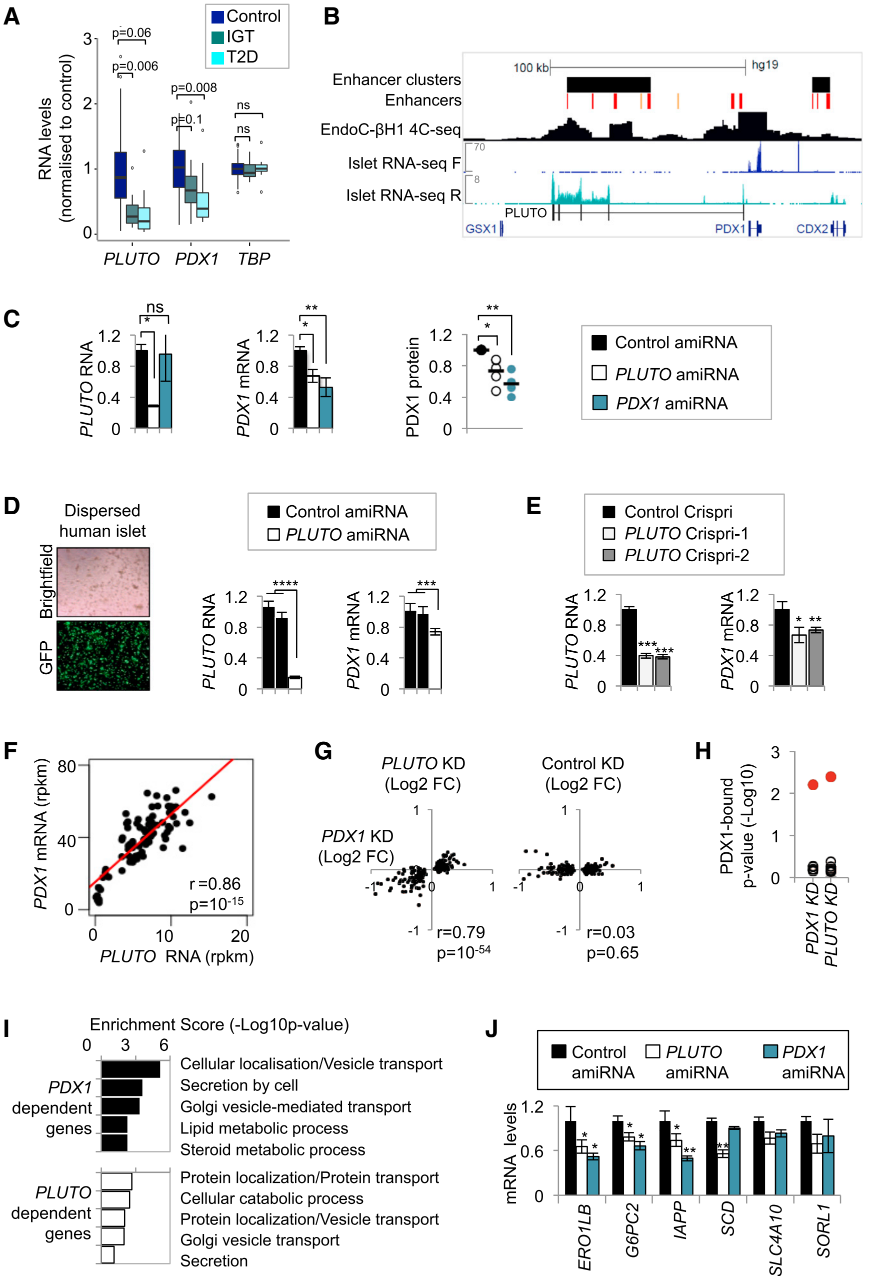

Figure 6. *PLUTO* Knockdown Decreases *PDX1* mRNA

(A) Downregulation of *PLUTO* (HI-LNC71) and *PDX1* in islets from donors with T2D or IGT. Differential expression analysis was performed on control (n = 50) versus T2D (n = 10) or IGT (n = 15) samples. Boxplots represent expression normalized to the mean of control samples. Adjusted p values are shown.

(B) Schematic of the human *PDX1* locus and its associated enhancer cluster. A 4C-seq analysis was designed to identify regions interacting with the *PDX1* promoter region in EndoC-βH1 cells. Red and orange vertical lines depict active and poised islet enhancers, respectively. F and R represent forward and reverse RNA-seq strands, respectively, and scales represent RPM. *PLUTO* (*HI-LNC71*) was generated from a de novo assembly of islet RNA-seq and differs from a transcript annotated in UCSC and RefSeq that originates from a *PDX1* intronic region.

(C) Downregulation of *PLUTO* or *PDX1* using amiRNAs resulted in reduced *PDX1* mRNA and protein levels. EndoC-βH1 cells were transduced with control (black), *PLUTO* (white), or *PDX1* (turquoise) amiRNA vectors 80 hr prior to harvest. RNA levels were assessed by qPCR, normalized to *TBP*, and expressed as fold over control amiRNA samples (n = 4). For protein quantification, PDX1 levels were first normalized to the average of TBP and H3 levels and then compared with the control amiRNA sample.

(D) Downregulation of *PLUTO* in human islet cells results in reduced *PDX1* mRNA levels. Islet cells were dispersed and transduced with amiRNA vectors (n = 3) as in (B).

(E) Downregulation of *PLUTO* in EndoC-βH3 cells using CRISPRi also decreases *PDX1* mRNA. EndoC-βH3 cells were nucleofected with CRISPRi vectors 80 hr prior to harvest. RNA levels were assessed by qPCR and normalized to *TBP* and then to a control CRISPRi sample (n = 3).

(F) *PDX1* and *PLUTO* RNA levels were highly correlated in 64 human islet samples.

(G) Knockdown of *PDX1* and *PLUTO* resulted in differential expression of similar genes. Fold change value (Log2) of top 250 dysregulated genes following the *PDX1* knockdown was plotted against the same genes following the *PLUTO* knockdown.

(H) GSEA showed that genes that were downregulated upon knockdown of *PDX1* and *PLUTO* were enriched in genes whose enhancers were bound by PDX1 (red) in islets but not in ten control gene sets (black) that were expressed at similar levels as PDX1-bound genes.

(I) Knockdown of *PDX1* and *PLUTO* resulted in differential expression of genes with similar biological process annotations.

(J) Examples of known *PDX1*-regulated genes that are also co-regulated by *PLUTO* in parallel knockdown experiments. mRNA levels were assessed as in (B). Error bars denote SEM; ***p < 10^{-3}, **p < 0.01, *p < 0.05 (Student's t test).

previous findings by demonstrating a functional role of lncRNAs in lineage-specific TF networks.

Our findings invite the question of what molecular mechanisms underlie the regulatory effects of β cell lncRNAs. LncRNAs have been proposed to control gene expression through diverse molecular mechanisms, including the formation of protein-specific interactions and scaffolds, RNA-DNA or RNA-RNA hybrids, the titration of miRNAs, and the modulation of 3D chromosomal structures (Rinn and Chang, 2012; Wang and Chang, 2011), whereas some transcripts currently defined as lncRNAs can theoretically encode for atypical small peptide sequences (Andrews and Rothnagel, 2014). Our knockdown and co-expression analyses have identified a subset of functional lncRNAs that appear to regulate a nearby gene, suggesting a lncRNA-based *cis*-regulatory mechanism, whereas others are likely to exert *trans*-regulatory effects. We focused on one

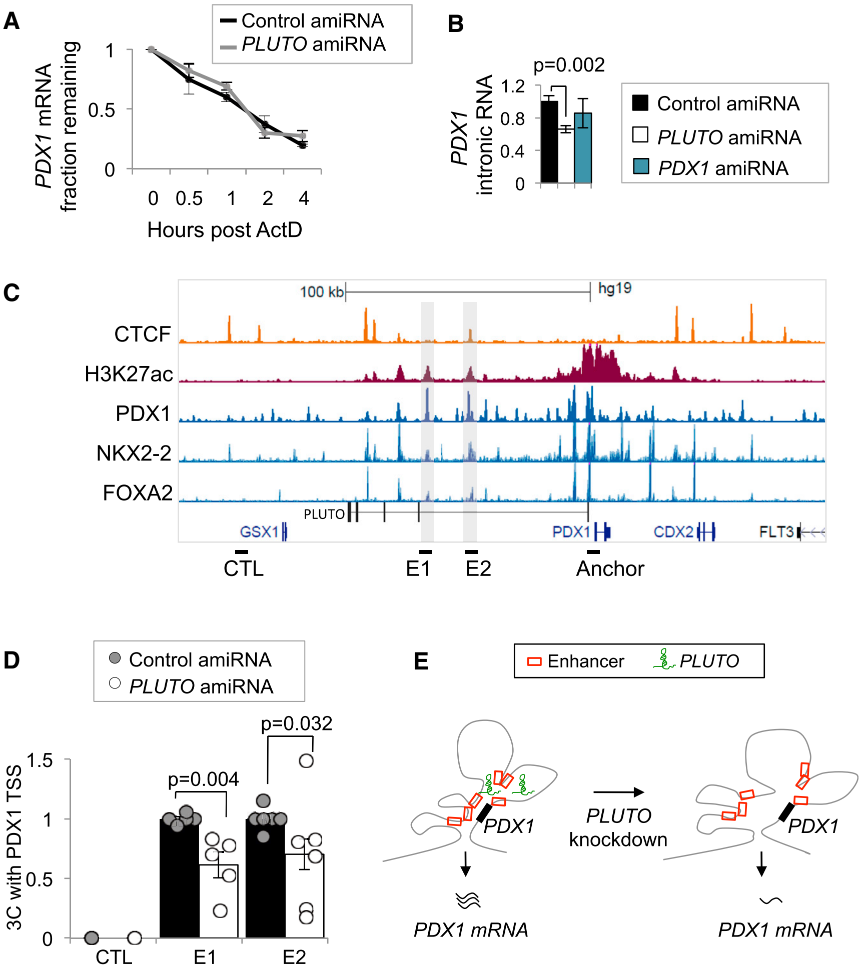

Figure 7. *PLUTO* Regulates *PDX1* Transcription and 3D Chromatin Structure

(A) The mRNA stability of *PDX1* was unaffected by *PLUTO* knockdown. *PDX1* mRNA was measured in control and *PLUTO* amiRNA knockdown in EndoC-βH1 cells after Actinomycin D (ActD) treatment (n = 3). mRNA levels are presented as a percentage of levels observed at time = 0.

(B) Knockdown of *PLUTO* was carried out as in Figure 6B, and this led to reduced *PDX1* transcription, as assessed by qPCR analysis of intronic *PDX1* RNA levels using hydrolysis probes. Values were normalized to *TBP* mRNA and expressed as fold over the control amiRNA sample (n = 4).

(C) Schematic of selected epigenomic features of the *PDX1* locus.

(D) *PLUTO* is required for 3D contacts between the *PDX1* promoter and distal enhancers. 3C analysis revealed that knockdown of *PLUTO* resulted in reduced contacts between the *PDX1* promoter (anchor) and two enhancers (E1 and E2). Interaction signals were normalized to a control region on the *PDX1* intron. CTL represents a negative control region that does not harbor interactions with the *PDX1* promoter. Error bars denote ± SEM, and p values are from a Student's t test.

(E) *PLUTO* knockdown resulted in impaired 3D contacts between the *PDX1* promoter and its adjacent enhancer cluster, causing reduced *PDX1* transcriptional activity.

functional nuclear-enriched β cell lncRNA, *PLUTO*, and found that its function in β cell networks is at least in part due to its ability to elicit an effect on the transcription of its adjacent gene, *PDX1*, which encodes a key β cell transcription factor. Importantly, this was observed for both the mouse and human orthologs, and similar effects were obtained through RNAi suppression or through CRISPR-induced transcriptional interference of *PLUTO*. Our studies further showed that *PLUTO* promotes 3D interactions between the *PDX1* promoter and its upstream enhancer cluster, which is contained within the body of the *PLUTO* gene. We thus propose that *PLUTO* regulates the 3D architecture of the enhancer cluster at the *PDX1* locus. This finding is reminiscent but distinct from earlier examples of non-coding RNA genes that modulate 3D chromosomal structure (Lai et al., 2013; Yao et al., 2010). Given that a significant number of lncRNAs are co-expressed with adjacent lineage-specific protein-coding genes, it is possible that the general regulatory paradigm described here is relevant to analogous lncRNA-protein coding gene pairs.

Taken together, our data implicate cell-specific lncRNAs in human β cell transcriptional programs. Given the importance of TFs in the pathophysiology of human diabetes and their role in β cell programming strategies, it now seems reasonable to explore whether β cell lncRNAs also play analogous roles (Bell and Polonsky, 2001; Flanagan et al., 2014; Zhou et al., 2008). The findings reported here therefore strengthen earlier suggestions that defects in β cell lncRNAs might contribute to the pathogenesis of human diabetes (Fadista et al., 2014; Morán et al., 2012) and warrant an assessment of whether they can be harnessed to promote β cell differentiation, function, or cellular mass.

EXPERIMENTAL PROCEDURES

Pancreatic Islets

Human islets used for RNA-seq and chromatin immunoprecipitation sequencing (ChIP-seq) were cultured with CMRL 1066 medium containing 10% fetal calf serum (FCS) before shipment, after which they were cultured for 3 days with RPMI 1640 medium containing 11 mM glucose and supplemented with 10% FCS.

Glucose-Stimulated Insulin Release

Glucose-stimulated insulin release was assayed in EndoC-βH1 or EndoC-βH3 cells as described previously (Benazra et al., 2015; Ravassard et al., 2011).

RNA Analysis

RNA was isolated with Tripure (Roche) and treated with DNase I (Sigma). qPCR was performed with SYBR green or Taqman probe detection (van Arensbergen et al., 2010). See Table S4 for oligonucleotide and probe sequences.

amiRNA and CRISPRi Experiments

Lentiviral vectors carrying amiRNAs targeting TFs, lncRNAs, and non-targeting control sequences were transduced into the EndoC-βH1 human β cell line as described previously (Castaing et al., 2005; Ravassard et al., 2011; Scharfmann et al., 2014).

Figure S1A illustrates the vector design. Oligonucleotide sequences are shown in Table S4. Non-transduced cells were assayed in parallel. Cells were harvested 80 hr post transduction for RNA extraction. For transduction of human islets, islets were first dispersed using trypsin-EDTA and gentle agitation. CRISPRi experiments were performed with two gRNAs designed to target *PLUTO* exon 1 or two unrelated intergenic control regions and transfected in EndoC-βH3 cells (Table S4).

Gene Expression Array Analysis

RNA was hybridized onto HTA2.0 Affymetrix arrays. RMA normalization was carried out using Expression Console (Affymetrix). Gene-based differential expression analysis was done using Transcriptome Analysis Console (TAC, Affymetrix). Enhancer cluster genes were defined by genes that were associated with clustered islet enhancers that show top 50 percentile binding by TFs (PDX1, FOXA2, NKX2-2, NKX6.1, and MAFB) as defined previously (Pasquali et al., 2014). Pancreatic islet gene sets used for enrichment analysis are shown in Table S5. A list of islet-enriched genes was generated as those with more than two SDs higher expression in human islets than the average expression in 16 human tissues (Table S5).

Differential Expression in IGT and T2D Islets

RNA-seq data have been described previously (Fadista et al., 2014). The samples were aligned to the hg19 genome using STAR aligner version 2.3.0 as described in the Supplemental Experimental Procedures, quantification was carried out with HTseq-Count 0.6.1, and differential expression analysis of lncRNA genes was done using DEseq2 1.10 (Table S3) using an adjusted p value threshold of 0.05.

3C

3C and 4C-seq was carried out as described previously (Pasquali et al., 2014; Tena et al., 2011) For real-time PCR quantification, readings were normalized to a control region within the *PDX1* intron. Normalized values are expressed as a fraction of non-targeting amiRNA control sample. See Table S4 for oligonucleotide sequences.

Annotation of Islet lncRNAs

LncRNAs were annotated through de novo assembly of ∼5 billion stranded paired-end RNA-seq reads from 41 human islet samples, filtered for expression in FACS-purified β cell cells, lack of enrichment in the pancreatic exocrine fraction to exclude acinar contaminants, and the presence of H3K4me3 enrichment in the vicinity of the 5′ end. A more detailed description of the annotation process is provided in the Supplemental Information. Annotations are available in Table S3 and can be accessed on a UCSC Genome Browser (GRCh37/hg19) session by selecting "track hubs" and "Human Islet lncRNAs." Alternatively, the track hub can be directly visualized in the UCSC Genome Browser using the following link: http://genome.ucsc.edu/cgi-bin/hgTracks?db=hg19&hubUrl=http://www.imperial.ac.uk/medicine/beta-cell-genome-regulation-laboratory/data/HILNCs/HILNCs.txt&hgS_loadUrlName=http://www.imperial.ac.uk/medicine/beta-cell-genome-regulation-laboratory/data/.

Network Analysis

The WGCNA(v2) tool was used to build a co-transcriptional network based on mRNAs from 64 human islet RNA-seq samples.

ACCESSION NUMBERS

The accession number for the microarray data reported in this paper is GEO: GSE83619.

SUPPLEMENTAL INFORMATION

Supplemental Information includes Supplemental Experimental Procedures, seven figures, and seven tables and can be found with this article online at http://dx.doi.org/10.1016/j.cmet.2016.11.016.

AUTHOR CONTRIBUTIONS

J.F. and I.A. conceived the idea, designed the experiments, and wrote the manuscript. J.F. supervised and I.A. coordinated the project. I.A., Z.T., H.W., J.Y., C.A., E.S., A.S., L. Pasquali, and D.M.Y.R. contributed to data analysis. I.A., A.B., M.B., C.S.C., R.G.F., J.G.H., and N.C. performed the experiments. D.M.Y.R., I.M., and N.N. annotated lncRNAs. L. Piemonti, T.B., C.B., J.K.C., and F.P. provided samples. I.A., J.F., Z.T., A.B., D.M.Y.R, L.G., C.B., J.K.C., F.P., P.R., A.S., L.G., C.A., and E.S. discussed the results. All authors read and approved the manuscript. Z.T., A.B., D.M.Y.R., C.S.C., and N.N. contributed equally.

ACKNOWLEDGMENTS

The research was supported by the National Institute for Health Research (NIHR) Imperial Biomedical Research Centre. Work was funded by grants from the Wellcome Trust (WT101033 to J.F.), NIH-BCBC (2U01 DK072473-06 to J.F. and P.R.), Ministerio de Economía y Competitividad (BFU2014-54284-R to J.F.), and Horizon 2020 (667191 to J.F.). Work in IDIBAPS was supported by the CERCA Programme, Generalitat de Catalunya. J.Y. was supported through a Berg and Unity Biotechnology fellowship. The authors are grateful to Helena Raurell Vila for experimental help and Romain Derelle and Loris Mularoni for advice regarding bioinformatics analysis. P.R. is a shareholder and consultant for Endocells/Unicercell Biosolutions. Z.T. receives financial support from Berg Pharma and Unity Biotechnology as a consultant.

Received: May 5, 2016
Revised: October 1, 2016
Accepted: November 29, 2016
Published: December 29, 2016

REFERENCES

Ahlgren, U., Jonsson, J., Jonsson, L., Simu, K., and Edlund, H. (1998). beta-cell-specific inactivation of the mouse Ipf1/Pdx1 gene results in loss of the beta-cell phenotype and maturity onset diabetes. Genes Dev. *12*, 1763–1768.

Anderson, K.R., Singer, R.A., Balderes, D.A., Hernandez-Lagunas, L., Johnson, C.W., Artinger, K.B., and Sussel, L. (2011). The L6 domain tetraspanin Tm4sf4 regulates endocrine pancreas differentiation and directed cell migration. Development *138*, 3213–3224.

Andrews, S.J., and Rothnagel, J.A. (2014). Emerging evidence for functional peptides encoded by short open reading frames. Nat. Rev. Genet. *15*, 193–204.

Bell, G.I., and Polonsky, K.S. (2001). Diabetes mellitus and genetically programmed defects in beta-cell function. Nature *414*, 788–791.

Benazra, M., Lecomte, M.J., Colace, C., Müller, A., Machado, C., Pechberty, S., Bricout-Neveu, E., Grenier-Godard, M., Solimena, M., Scharfmann, R., et al. (2015). A human beta cell line with drug inducible excision of immortalizing transgenes. Mol. Metab. *4*, 916–925.

Benner, C., van der Meulen, T., Cacéres, E., Tigyi, K., Donaldson, C.J., and Huising, M.O. (2014). The transcriptional landscape of mouse beta cells compared to human beta cells reveals notable species differences in long non-coding RNA and protein-coding gene expression. BMC Genomics *15*, 620.

Cabili, M.N., Trapnell, C., Goff, L., Koziol, M., Tazon-Vega, B., Regev, A., and Rinn, J.L. (2011). Integrative annotation of human large intergenic noncoding RNAs reveals global properties and specific subclasses. Genes Dev. *25*, 1915–1927.

Carninci, P., Kasukawa, T., Katayama, S., Gough, J., Frith, M.C., Maeda, N., Oyama, R., Ravasi, T., Lenhard, B., Wells, C., et al.; FANTOM Consortium; RIKEN Genome Exploration Research Group and Genome Science Group (Genome Network Project Core Group) (2005). The transcriptional landscape of the mammalian genome. Science *309*, 1559–1563.

Carrieri, C., Cimatti, L., Biagioli, M., Beugnet, A., Zucchelli, S., Fedele, S., Pesce, E., Ferrer, I., Collavin, L., Santoro, C., et al. (2012). Long non-coding

antisense RNA controls Uchl1 translation through an embedded SINEB2 repeat. Nature *491*, 454–457.

Castaing, M., Guerci, A., Mallet, J., Czernichow, P., Ravassard, P., and Scharfmann, R. (2005). Efficient restricted gene expression in beta cells by lentivirus-mediated gene transfer into pancreatic stem/progenitor cells. Diabetologia *48*, 709–719.

Chen, L.L., and Carmichael, G.G. (2009). Altered nuclear retention of mRNAs containing inverted repeats in human embryonic stem cells: functional role of a nuclear noncoding RNA. Mol. Cell *35*, 467–478.

Derrien, T., Johnson, R., Bussotti, G., Tanzer, A., Djebali, S., Tilgner, H., Guernec, G., Martin, D., Merkel, A., Knowles, D.G., et al. (2012). The GENCODE v7 catalog of human long noncoding RNAs: analysis of their gene structure, evolution, and expression. Genome Res. *22*, 1775–1789.

Derry, J.M., Zhong, H., Molony, C., MacNeil, D., Guhathakurta, D., Zhang, B., Mudgett, J., Small, K., El Fertak, L., Guimond, A., et al. (2010). Identification of genes and networks driving cardiovascular and metabolic phenotypes in a mouse F2 intercross. PLoS ONE *5*, e14319.

Fadista, J., Vikman, P., Laakso, E.O., Mollet, I.G., Esguerra, J.L., Taneera, J., Storm, P., Osmark, P., Ladenvall, C., Prasad, R.B., et al. (2014). Global genomic and transcriptomic analysis of human pancreatic islets reveals novel genes influencing glucose metabolism. Proc. Natl. Acad. Sci. USA *111*, 13924–13929.

Flanagan, S.E., De Franco, E., Lango Allen, H., Zerah, M., Abdul-Rasoul, M.M., Edge, J.A., Stewart, H., Alamiri, E., Hussain, K., Wallis, S., et al. (2014). Analysis of transcription factors key for mouse pancreatic development establishes NKX2-2 and MNX1 mutations as causes of neonatal diabetes in man. Cell Metab. *19*, 146–154.

Goff, L.A., Groff, A.F., Sauvageau, M., Trayes-Gibson, Z., Sanchez-Gomez, D.B., Morse, M., Martin, R.D., Elcavage, L.E., Liapis, S.C., Gonzalez-Celeiro, M., et al. (2015). Spatiotemporal expression and transcriptional perturbations by long noncoding RNAs in the mouse brain. Proc. Natl. Acad. Sci. USA *112*, 6855–6862.

Gong, C., and Maquat, L.E. (2011). lncRNAs transactivate STAU1-mediated mRNA decay by duplexing with 3′ UTRs via Alu elements. Nature *470*, 284–288.

Guttman, M., Amit, I., Garber, M., French, C., Lin, M.F., Feldser, D., Huarte, M., Zuk, O., Carey, B.W., Cassady, J.P., et al. (2009). Chromatin signature reveals over a thousand highly conserved large non-coding RNAs in mammals. Nature *458*, 223–227.

Guttman, M., Donaghey, J., Carey, B.W., Garber, M., Grenier, J.K., Munson, G., Young, G., Lucas, A.B., Ach, R., Bruhn, L., et al. (2011). lincRNAs act in the circuitry controlling pluripotency and differentiation. Nature *477*, 295–300.

Huarte, M., Guttman, M., Feldser, D., Garber, M., Koziol, M.J., Kenzelmann-Broz, D., Khalil, A.M., Zuk, O., Amit, I., Rabani, M., et al. (2010). A large intergenic noncoding RNA induced by p53 mediates global gene repression in the p53 response. Cell *142*, 409–419.

Hung, T., Wang, Y., Lin, M.F., Koegel, A.K., Kotake, Y., Grant, G.D., Horlings, H.M., Shah, N., Umbricht, C., Wang, P., et al. (2011). Extensive and coordinated transcription of noncoding RNAs within cell-cycle promoters. Nat. Genet. *43*, 621–629.

Iyer, M.K., Niknafs, Y.S., Malik, R., Singhal, U., Sahu, A., Hosono, Y., Barrette, T.R., Prensner, J.R., Evans, J.R., Zhao, S., et al. (2015). The landscape of long noncoding RNAs in the human transcriptome. Nat. Genet. *47*, 199–208.

Jonsson, J., Carlsson, L., Edlund, T., and Edlund, H. (1994). Insulin-promoter-factor 1 is required for pancreas development in mice. Nature *371*, 606–609.

Kim, S.K., Lund, J., Kiraly, M., Duke, K., Jiang, M., Stuart, J.M., Eizinger, A., Wylie, B.N., and Davidson, G.S. (2001). A gene expression map for Caenorhabditis elegans. Science *293*, 2087–2092.

Klattenhoff, C.A., Scheuermann, J.C., Surface, L.E., Bradley, R.K., Fields, P.A., Steinhauser, M.L., Ding, H., Butty, V.L., Torrey, L., Haas, S., et al. (2013). Braveheart, a long noncoding RNA required for cardiovascular lineage commitment. Cell *152*, 570–583.

Kretz, M., Siprashvili, Z., Chu, C., Webster, D.E., Zehnder, A., Qu, K., Lee, C.S., Flockhart, R.J., Groff, A.F., Chow, J., et al. (2013). Control of somatic tissue differentiation by the long non-coding RNA TINCR. Nature *493*, 231–235.

Ku, G.M., Kim, H., Vaughn, I.W., Hangauer, M.J., Myung Oh, C., German, M.S., and McManus, M.T. (2012). Research resource: RNA-Seq reveals unique features of the pancreatic β-cell transcriptome. Mol. Endocrinol. *26*, 1783–1792.

Lai, F., Orom, U.A., Cesaroni, M., Beringer, M., Taatjes, D.J., Blobel, G.A., and Shiekhattar, R. (2013). Activating RNAs associate with Mediator to enhance chromatin architecture and transcription. Nature *494*, 497–501.

Li, C., Chen, P., Palladino, A., Narayan, S., Russell, L.K., Sayed, S., Xiong, G., Chen, J., Stokes, D., Butt, Y.M., et al. (2010). Mechanism of hyperinsulinism in short-chain 3-hydroxyacyl-CoA dehydrogenase deficiency involves activation of glutamate dehydrogenase. J. Biol. Chem. *285*, 31806–31818.

Louagie, E., Taylor, N.A., Flamez, D., Roebroek, A.J., Bright, N.A., Meulemans, S., Quintens, R., Herrera, P.L., Schuit, F., Van de Ven, W.J., and Creemers, J.W. (2008). Role of furin in granular acidification in the endocrine pancreas: identification of the V-ATPase subunit Ac45 as a candidate substrate. Proc. Natl. Acad. Sci. USA *105*, 12319–12324.

Luco, R.F., and Misteli, T. (2011). More than a splicing code: integrating the role of RNA, chromatin and non-coding RNA in alternative splicing regulation. Curr. Opin. Genet. Dev. *21*, 366–372.

Morán, I., Akerman, I., van de Bunt, M., Xie, R., Benazra, M., Nammo, T., Arnes, L., Nakić, N., García-Hurtado, J., Rodríguez-Seguí, S., et al. (2012). Human β cell transcriptome analysis uncovers lncRNAs that are tissue-specific, dynamically regulated, and abnormally expressed in type 2 diabetes. Cell Metab. *16*, 435–448.

Offield, M.F., Jetton, T.L., Labosky, P.A., Ray, M., Stein, R.W., Magnuson, M.A., Hogan, B.L., and Wright, C.V. (1996). PDX-1 is required for pancreatic outgrowth and differentiation of the rostral duodenum. Development *122*, 983–995.

Okamoto, K., Iwasaki, N., Doi, K., Noiri, E., Iwamoto, Y., Uchigata, Y., Fujita, T., and Tokunaga, K. (2012). Inhibition of glucose-stimulated insulin secretion by KCNJ15, a newly identified susceptibility gene for type 2 diabetes. Diabetes *61*, 1734–1741.

Okazaki, Y., Furuno, M., Kasukawa, T., Adachi, J., Bono, H., Kondo, S., Nikaido, I., Osato, N., Saito, R., Suzuki, H., et al.; FANTOM Consortium; RIKEN Genome Exploration Research Group Phase I & II Team (2002). Analysis of the mouse transcriptome based on functional annotation of 60,770 full-length cDNAs. Nature *420*, 563–573.

Pandey, G., Zhang, B., Chang, A.N., Myers, C.L., Zhu, J., Kumar, V., and Schadt, E.E. (2010). An integrative multi-network and multi-classifier approach to predict genetic interactions. PLoS Comput. Biol. *6*, 6.

Pang, K.C., Frith, M.C., and Mattick, J.S. (2006). Rapid evolution of noncoding RNAs: lack of conservation does not mean lack of function. Trends Genet. *22*, 1–5.

Pasquali, L., Gaulton, K.J., Rodríguez-Seguí, S.A., Mularoni, L., Miguel-Escalada, I., Akerman, I., Tena, J.J., Morán, I., Gómez-Marín, C., van de Bunt, M., et al. (2014). Pancreatic islet enhancer clusters enriched in type 2 diabetes risk-associated variants. Nat. Genet. *46*, 136–143.

Pauli, A., Valen, E., Lin, M.F., Garber, M., Vastenhouw, N.L., Levin, J.Z., Fan, L., Sandelin, A., Rinn, J.L., Regev, A., and Schier, A.F. (2012). Systematic identification of long noncoding RNAs expressed during zebrafish embryogenesis. Genome Res. *22*, 577–591.

Penny, G.D., Kay, G.F., Sheardown, S.A., Rastan, S., and Brockdorff, N. (1996). Requirement for Xist in X chromosome inactivation. Nature *379*, 131–137.

Pott, S., and Lieb, J.D. (2015). What are super-enhancers? Nat. Genet. *47*, 8–12.

Qiu, Y., Guo, M., Huang, S., and Stein, R. (2002). Insulin gene transcription is mediated by interactions between the p300 coactivator and PDX-1, BETA2, and E47. Mol. Cell. Biol. *22*, 412–420.

Ravassard, P., Hazhouz, Y., Pechberty, S., Bricout-Neveu, E., Armanet, M., Czernichow, P., and Scharfmann, R. (2011). A genetically engineered human

pancreatic β cell line exhibiting glucose-inducible insulin secretion. J. Clin. Invest. *121*, 3589–3597.

Rinn, J.L., and Chang, H.Y. (2012). Genome regulation by long noncoding RNAs. Annu. Rev. Biochem. *81*, 145–166.

Scharfmann, R., Pechberty, S., Hazhouz, Y., von Bülow, M., Bricout-Neveu, E., Grenier-Godard, M., Guez, F., Rachdi, L., Lohmann, M., Czernichow, P., and Ravassard, P. (2014). Development of a conditionally immortalized human pancreatic β cell line. J. Clin. Invest. *124*, 2087–2098.

Schmitt, A.M., and Chang, H.Y. (2013). Gene regulation: Long RNAs wire up cancer growth. Nature *500*, 536–537.

Segal, E., Shapira, M., Regev, A., Pe'er, D., Botstein, D., Koller, D., and Friedman, N. (2003). Module networks: identifying regulatory modules and their condition-specific regulators from gene expression data. Nat. Genet. *34*, 166–176.

Sleutels, F., Zwart, R., and Barlow, D.P. (2002). The non-coding Air RNA is required for silencing autosomal imprinted genes. Nature *415*, 810–813.

Smith, S.B., Qu, H.Q., Taleb, N., Kishimoto, N.Y., Scheel, D.W., Lu, Y., Patch, A.M., Grabs, R., Wang, J., Lynn, F.C., et al. (2010). Rfx6 directs islet formation and insulin production in mice and humans. Nature *463*, 775–780.

Stoffers, D.A., Zinkin, N.T., Stanojevic, V., Clarke, W.L., and Habener, J.F. (1997). Pancreatic agenesis attributable to a single nucleotide deletion in the human IPF1 gene coding sequence. Nat. Genet. *15*, 106–110.

Stuart, J.M., Segal, E., Koller, D., and Kim, S.K. (2003). A gene-coexpression network for global discovery of conserved genetic modules. Science *302*, 249–255.

Su, W.L., Kleinhanz, R.R., and Schadt, E.E. (2011). Characterizing the role of miRNAs within gene regulatory networks using integrative genomics techniques. Mol. Syst. Biol. *7*, 490.

Tena, J.J., Alonso, M.E., de la Calle-Mustienes, E., Splinter, E., de Laat, W., Manzanares, M., and Gómez-Skarmeta, J.L. (2011). An evolutionarily conserved three-dimensional structure in the vertebrate Irx clusters facilitates enhancer sharing and coregulation. Nat. Commun. *2*, 310.

Tian, G., Sågetorp, J., Xu, Y., Shuai, H., Degerman, E., and Tengholm, A. (2012). Role of phosphodiesterases in the shaping of sub-plasma-membrane cAMP oscillations and pulsatile insulin secretion. J. Cell Sci. *125*, 5084–5095.

Ulitsky, I., Shkumatava, A., Jan, C.H., Sive, H., and Bartel, D.P. (2011). Conserved function of lincRNAs in vertebrate embryonic development despite rapid sequence evolution. Cell *147*, 1537–1550.

van Arensbergen, J., García-Hurtado, J., Moran, I., Maestro, M.A., Xu, X., Van de Casteele, M., Skoudy, A.L., Palassini, M., Heimberg, H., and Ferrer, J. (2010). Derepression of Polycomb targets during pancreatic organogenesis allows insulin-producing beta-cells to adopt a neural gene activity program. Genome Res. *20*, 722–732.

Varadi, A., and Rutter, G.A. (2002). Dynamic imaging of endoplasmic reticulum Ca2+ concentration in insulin-secreting MIN6 Cells using recombinant targeted cameleons: roles of sarco(endo)plasmic reticulum Ca2+-ATPase (SERCA)-2 and ryanodine receptors. Diabetes *51* (*Suppl 1*), S190–S201.

Wagner, T.F., Loch, S., Lambert, S., Straub, I., Mannebach, S., Mathar, I., Düfer, M., Lis, A., Flockerzi, V., Philipp, S.E., and Oberwinkler, J. (2008). Transient receptor potential M3 channels are ionotropic steroid receptors in pancreatic beta cells. Nat. Cell Biol. *10*, 1421–1430.

Wang, K.C., and Chang, H.Y. (2011). Molecular mechanisms of long noncoding RNAs. Mol. Cell *43*, 904–914.

Willingham, A.T., Orth, A.P., Batalov, S., Peters, E.C., Wen, B.G., Aza-Blanc, P., Hogenesch, J.B., and Schultz, P.G. (2005). A strategy for probing the function of noncoding RNAs finds a repressor of NFAT. Science *309*, 1570–1573.

Wilson, M.E., Scheel, D., and German, M.S. (2003). Gene expression cascades in pancreatic development. Mech. Dev. *120*, 65–80.

Yang, Y.H., Manning Fox, J.E., Zhang, K.L., MacDonald, P.E., and Johnson, J.D. (2013). Intraislet SLIT-ROBO signaling is required for beta-cell survival and potentiates insulin secretion. Proc. Natl. Acad. Sci. USA *110*, 16480–16485.

Yao, H., Brick, K., Evrard, Y., Xiao, T., Camerini-Otero, R.D., and Felsenfeld, G. (2010). Mediation of CTCF transcriptional insulation by DEAD-box RNA-binding protein p68 and steroid receptor RNA activator SRA. Genes Dev. *24*, 2543–2555.

Zhou, Q., Brown, J., Kanarek, A., Rajagopal, J., and Melton, D.A. (2008). In vivo reprogramming of adult pancreatic exocrine cells to beta-cells. Nature *455*, 627–632.

Zito, E., Chin, K.T., Blais, J., Harding, H.P., and Ron, D. (2010). ERO1-beta, a pancreas-specific disulfide oxidase, promotes insulin biogenesis and glucose homeostasis. J. Cell Biol. *188*, 821–832.

Cell Metabolism, Volume *25*

Supplemental Information

Human Pancreatic β Cell lncRNAs Control Cell-Specific Regulatory Networks

Ildem Akerman, Zhidong Tu, Anthony Beucher, Delphine M.Y. Rolando, Claire Sauty-Colace, Marion Benazra, Nikolina Nakic, Jialiang Yang, Huan Wang, Lorenzo Pasquali, Ignasi Moran, Javier Garcia-Hurtado, Natalia Castro, Roser Gonzalez-Franco, Andrew F. Stewart, Caroline Bonner, Lorenzo Piemonti, Thierry Berney, Leif Groop, Julie Kerr-Conte, Francois Pattou, Carmen Argmann, Eric Schadt, Philippe Ravassard, and Jorge Ferrer

Figure S1. Loss of function of beta cell lncRNAs, related to Figure 1 (part 1 of 6)

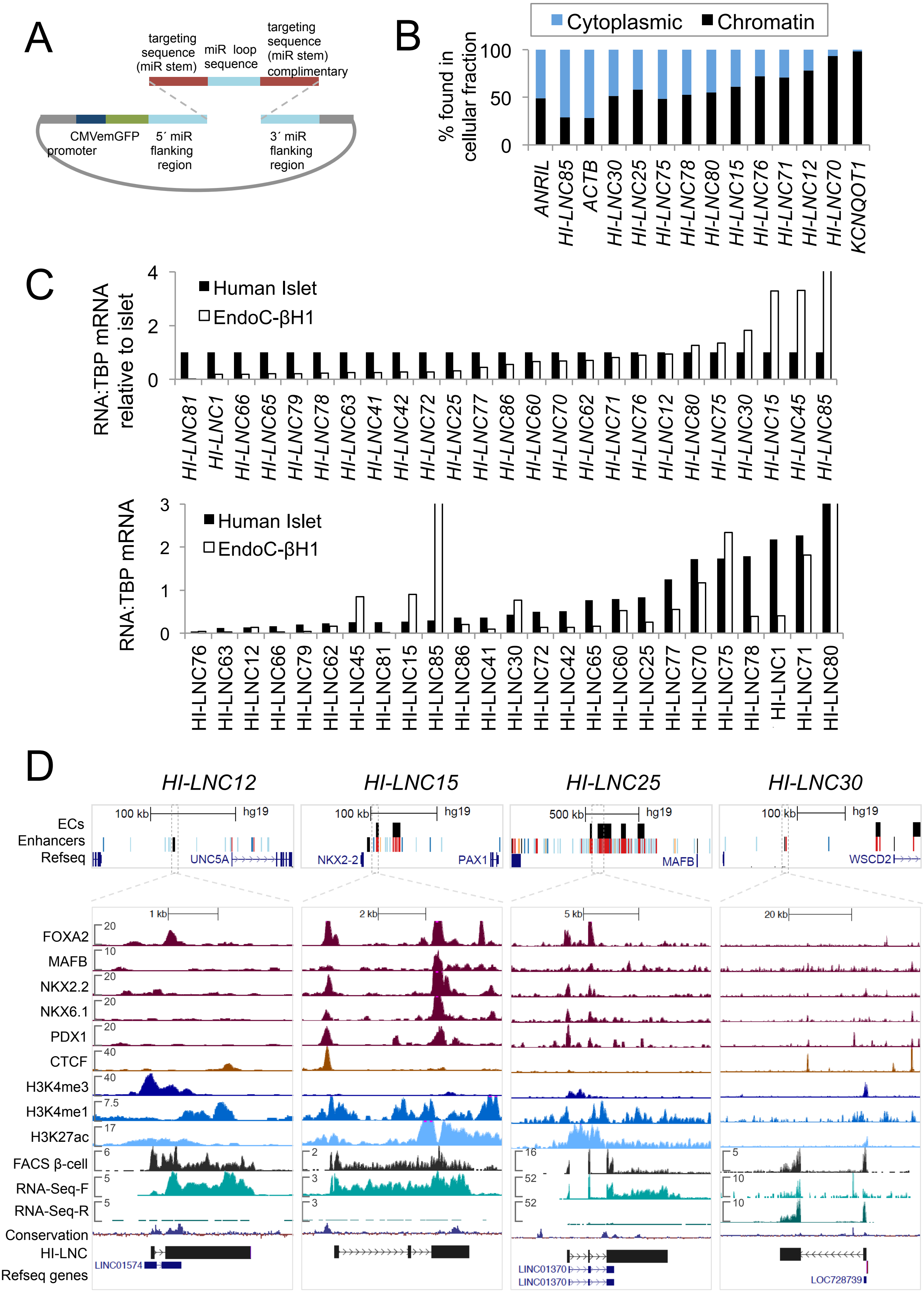

Figure S1. Loss of function of beta cell lncRNAs, related to Figure 1 (part 2 of 6)

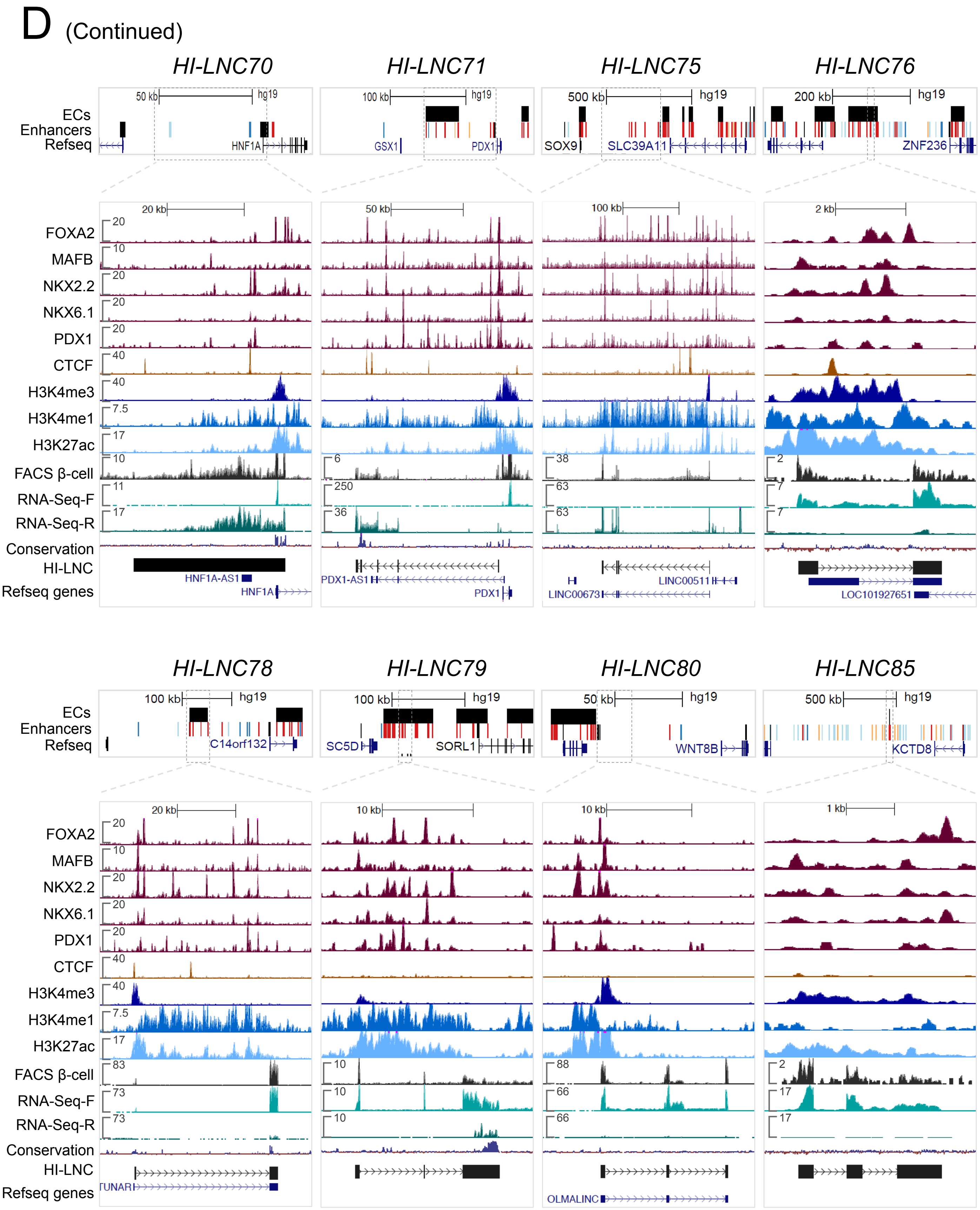

Figure S1. Loss of function of beta cell lncRNAs, related to Figure 1 (part 3 of 6)

E

		Replicate1		Replicate2	
		amiRNA-1	ami-RNA-2	amiRNA-1	ami-RNA-2
TFs	GLIS3	0.31	0.33	0.34	0.32
	HNF1a	0.50	0.47	0.50	0.52
	MAFB	0.53	0.41	0.51	0.44
	NKX2.2	0.50	0.43	0.12	0.33
	PDX1	0.34	0.44	0.35	0.50
HI-LNCs with 2 amiRNAs per target	HI-LNC12	0.44	0.48	0.51	0.54
	HI-LNC15	0.13	0.39	0.12	0.40
	HI-LNC30	0.27	0.24	0.30	0.47
	HI-LNC75	0.45	0.45	0.48	0.44
	HI-LNC76	0.46	0.35	0.43	0.55
	HI-LNC78	0.38	0.30	0.46	0.31
	HI-LNC80	0.34	0.40	0.35	0.46
		Replicate1	Replicate2	Replicate3	
HI-LNCs with one amiRNA per target	HI-LNC25	0.42	0.33	0.39	
	HI-LNC70	0.48	0.48	na	
	HI-LNC71	0.14	0.25	0.14	
	HI-LNC79	0.18	0.17	0.16	
	HI-LNC85	0.21	0.17	0.25	

F

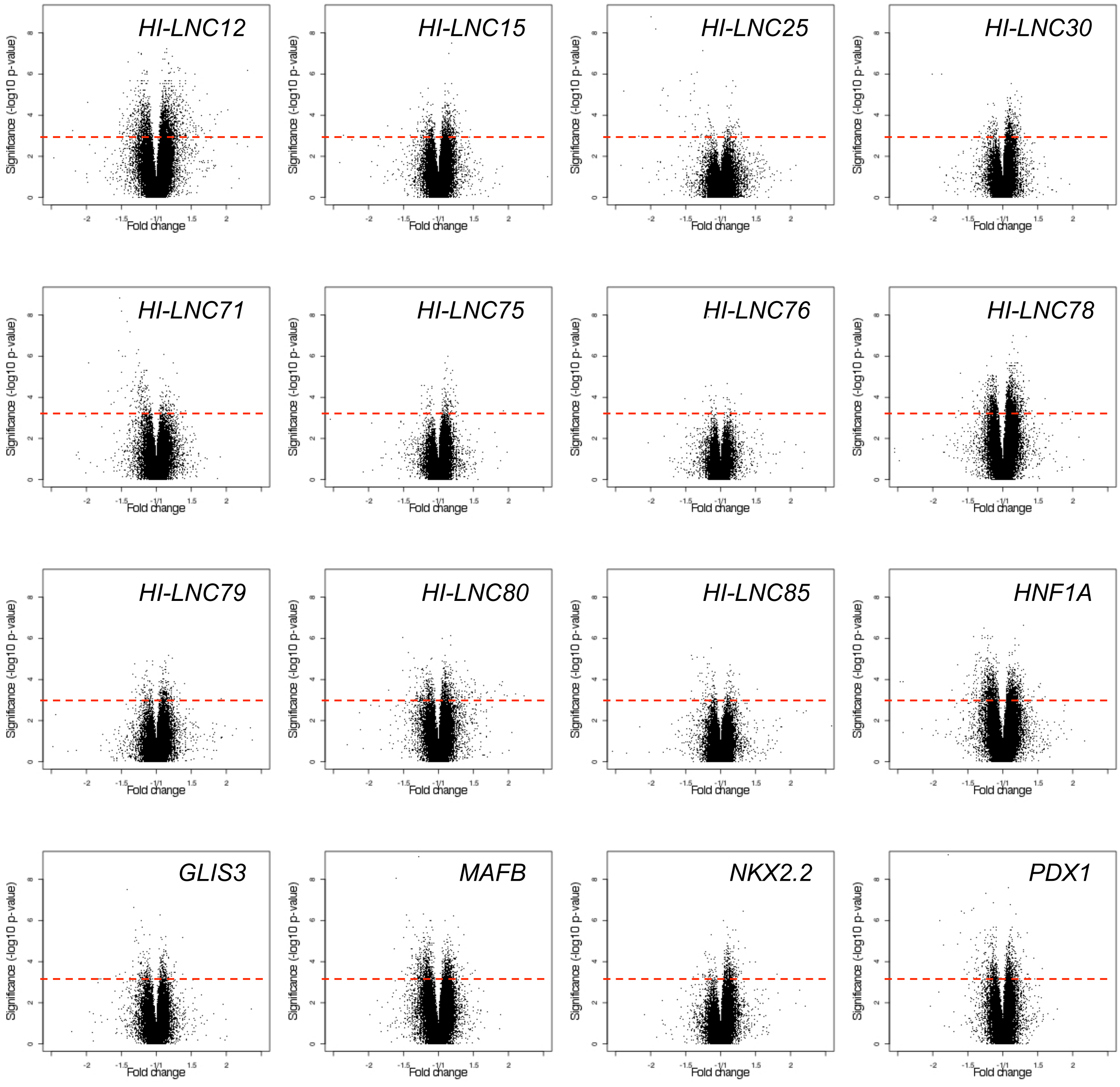

Figure S1. Loss of function of beta cell lncRNAs, related to Figure 1 (part 4 of 6)

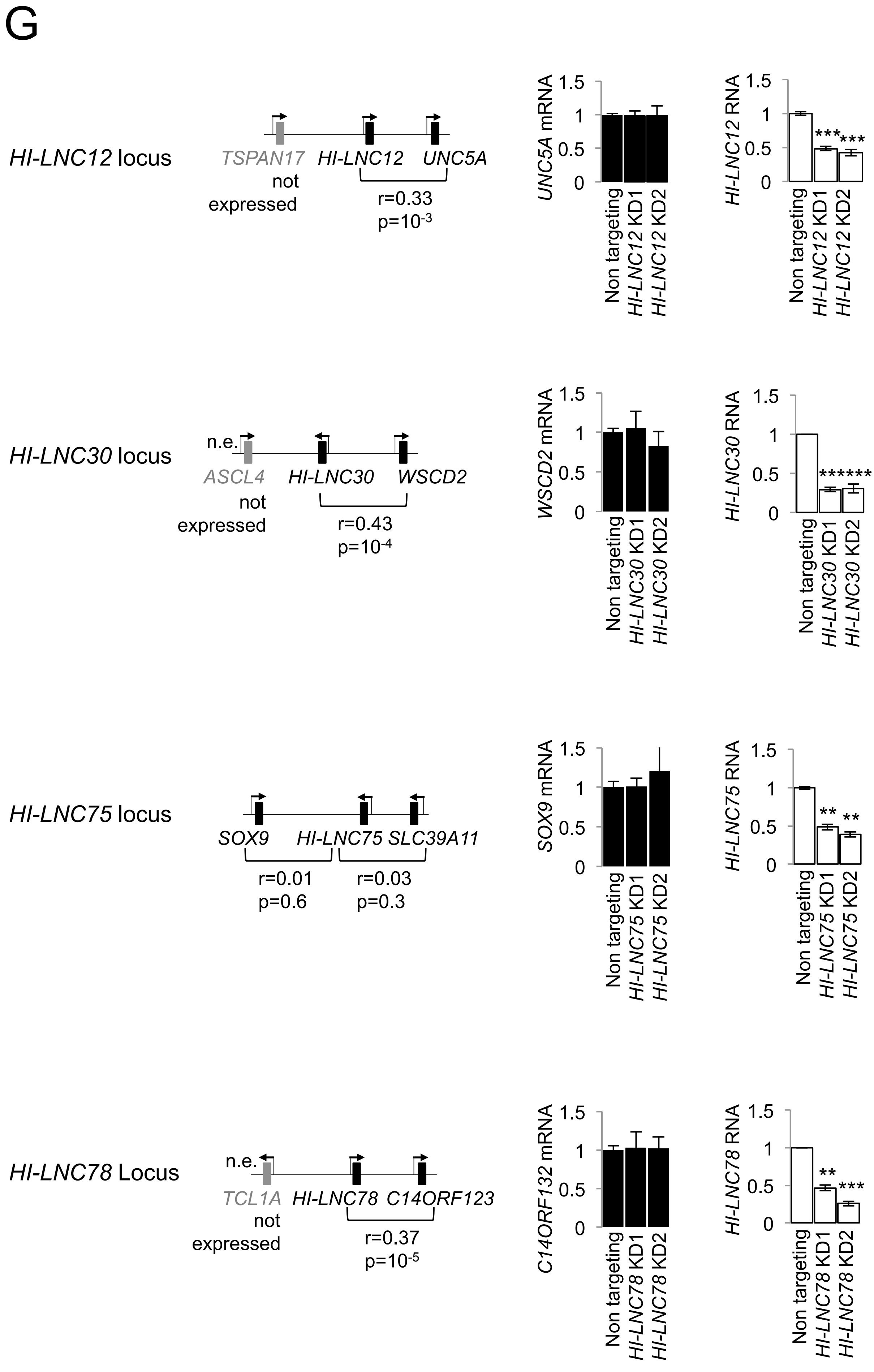

Figure S1. Loss of function of beta cell lncRNAs, related to Figure 1 (part 5 of 5)

G(Continued)

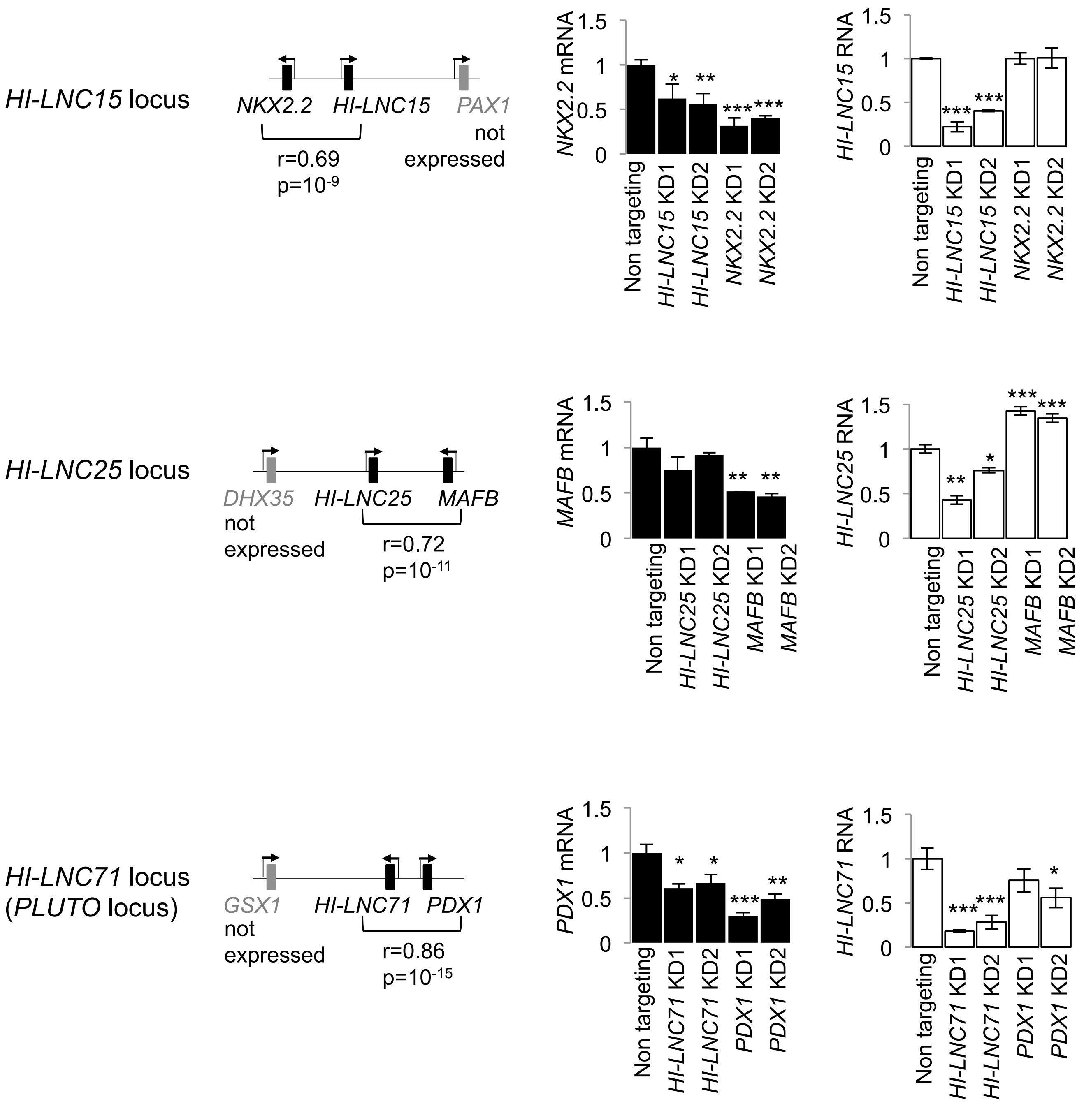

Figure S1. Loss of function of beta cell lncRNAs, related to Figure 1 (part 6 of 6)

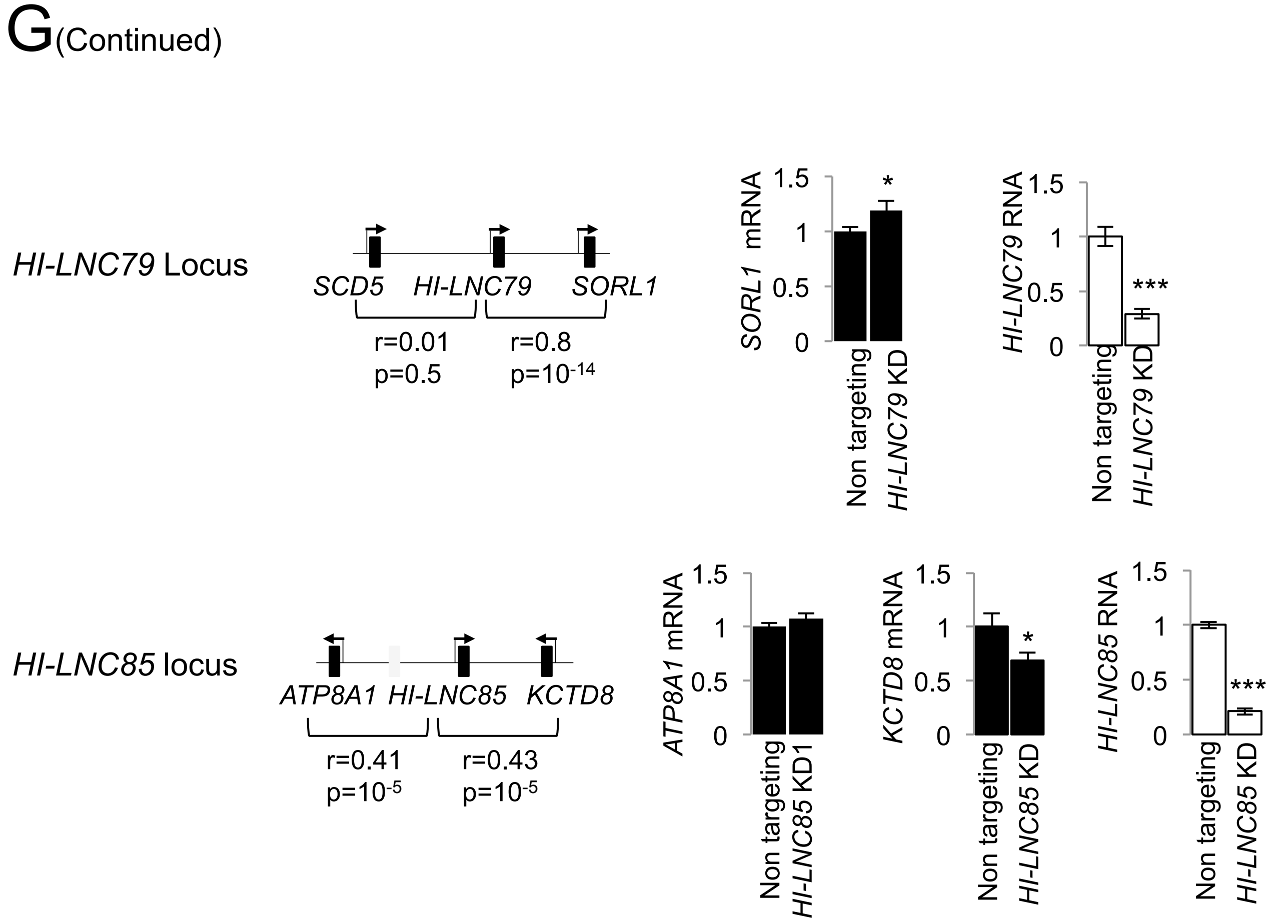

Figure S1. Loss of function of beta cell lncRNAs (A) A schematic of the vector used for generating amiRNAs. **(B)** Subcellular fractionation of islet lncRNAs. in EndoC-βH3 cells. **(C)** Relative expression of 25 shortlisted lncRNAs in human islets and in EndoC-βH1 cells. RNA levels were analyzed by qPCR, normalized to *TBP* mRNA (lower panel) and then to the levels in human islets (upper panel). **(D)** Genome browser images of the 12 human beta cell lncRNA genes selected for functional analysis. RNA-seq F:forward strand, R:reverse strand, axis represent RPM. HI-LNC track shows prevalent gene structures in human islet RNA. The top inset shows islet enhancer clusters (ECs), islet enhancers in red lines, and RefSeq annotations of nearby genes. **(E)** Knockdown efficiency for each amiRNA. The fraction of RNA remaining is shown. **(F)** Volcano plots of differential gene expression. Red lines indicate $p=10^{-3}$. **(G)** Regulatory interactions between islet lncRNAs and neighboring genes. Knowckdown experiments show that *HI-LNC15*, *HI-LNC79*, *HI-LNC85,* and *HI-LNC71(PLUTO)* regulate a neighboring gene (right panels), and furthermore display a high expression correlation with the neighboring gene in a panel of human islets (left panels). Other tested lncRNAs (*HI-LNC12, HI-LNC25, HI-LNC30, HI-LNC75, HI-LNC78*) do not affect adjacent genes, and display comparatively lower correlation of gene expression with the neighboring genes in islet samples. The left panel depicts Pearson correlation and associated p values for RNA levels in 64 human islet samples. n.e. indicates that the gene is not expressed in islets. The right panel depicts RNA levels normalized to *TBP* mRNAsequences used for knockdown of and then to control amiRNA samples. KD1 and KD2 represent different amiRNAs for indicated transcripts. Note that *HI-LNC25* and *MAFB* are highly co-expressed in human islets despite that *HI-LNC25* does not significantly regulate *MAFB* expression, although *MAFB* regulates *HI-LNC25*. Error bars denote ±SEM. ***p<0.001, **p<0.01, *p<0.05, Student´s t test, n=3 independent experiments.

Figure S2. Knockdown of lncRNAs causes impaired insulin secretion (related to Figure 2)

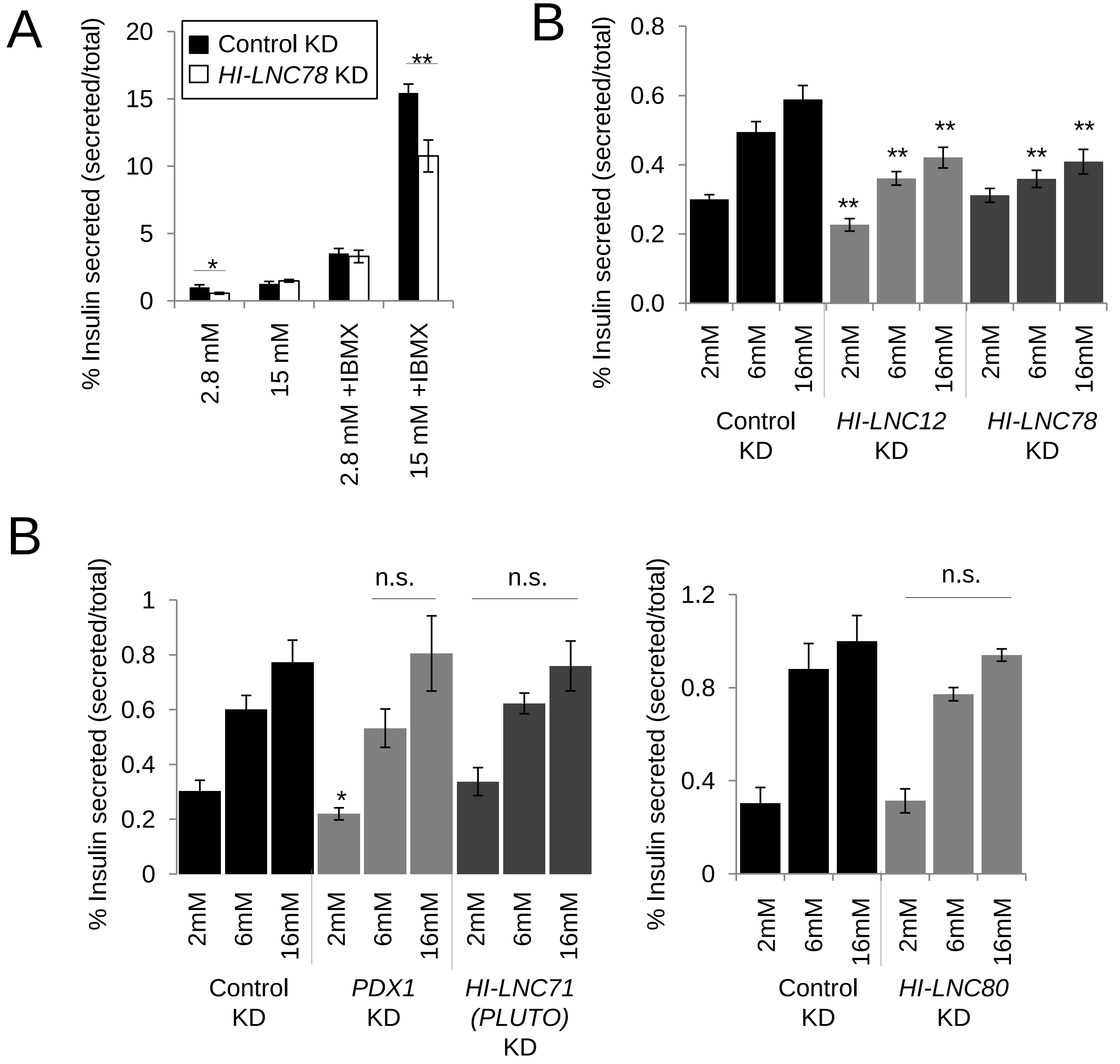

Figure S2. Knockdown of lncRNAs causes impaired insulin secretion. (A) Glucose stimulated insulin secretion was tested on T-antigen excised EndoC-βH3 cells after transduction with amiRNAs targeting *HI-LNC78* or controls. Secreted insulin content was normalized to total insulin content and expressed as fold change over control amiRNA treatment at 2.8 mM glucose. Each bar represents an average of two independent amiRNA vectors and 12 independent wells, from two independent experiments. Error bars represent ±SEM, ** p<0.01, *p<0.05 (Student's t-test). (B) Glucose-stimulated insulin secretion was measured in EndoC-βH3 cells 80 hours after nucleofection with locked nucleic acid (LNA) oligonucleotides to provide independent confirmation for amiRNA-based inhibition studies. Secreted insulin (ng/ml) was normalized to total insulin content and expressed as a percentage of total insulin content.. Each bar represents an average of two independent LNAs from four independent experiments. Error bars represent ±SEM. *p<0.05. ** p<0.01, n.s.=not significant, Student's t test, compared with control knock-downs in the same glucose concentration.

Figure S3. Knockdown of human islet TFs and lncRNAs leads causes shared transcriptional phenotypes, related to Figure 3

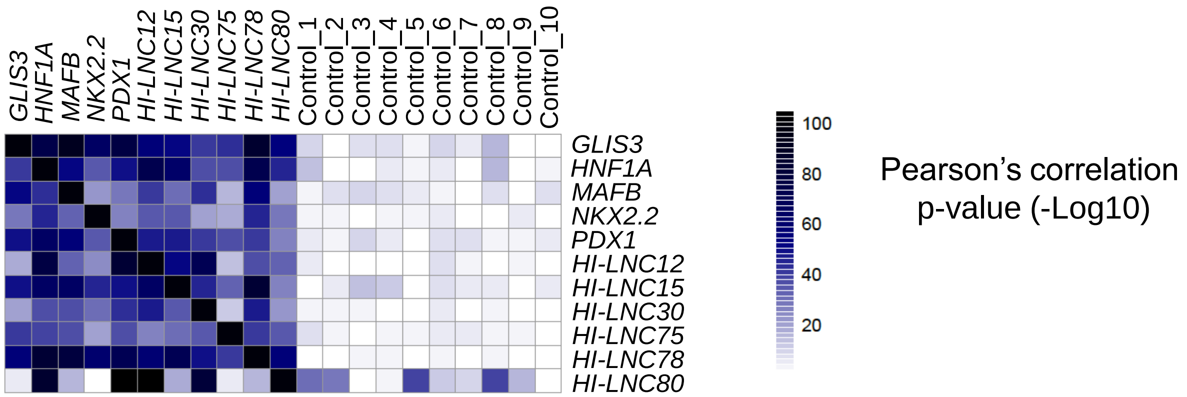

Figure S3. Knockdown of human islet TFs and lncRNAs leads causes shared transcriptional phenotypes. Heatmap of p values for Pearson´s correlation analysis of changes in gene expression for all pairwise comparisons of knockdowns that showed transcriptional phenotypes. The fold change of top 250 targets of each knockdown (vertical orientation) was correlated to the fold change of the same genes in other knockdowns and control experiments (horizontal orientation).

Figure S4. Islet specific TFs and lncRNAs regulate cell-specific programs, related to Figure 4

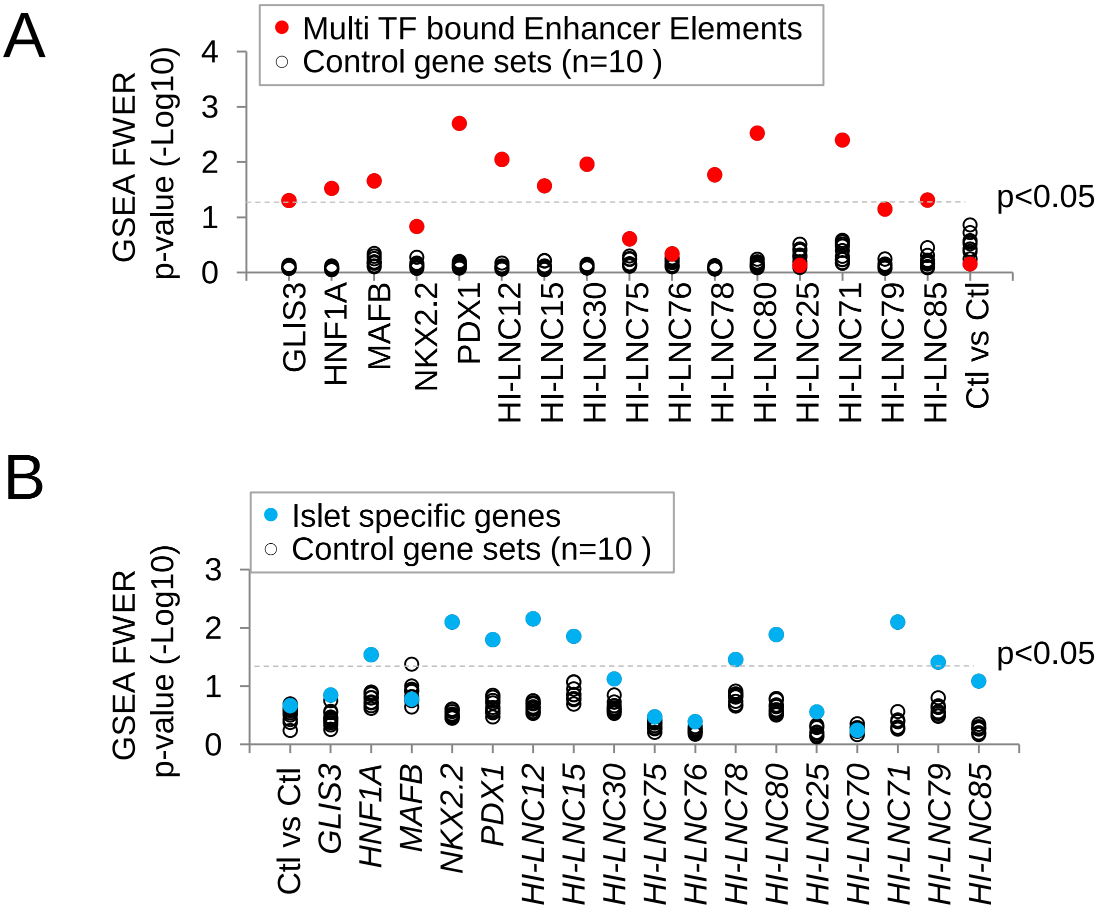

Figure S4. Islet-specific TFs and lncRNAs regulate cell-specific cis-regulatory programs. (A) TF and lncRNA-dependent genes are associated with enhancers that show high occupancy by islet-specific TFs. Gene Set Enrichment Analysis (GSEA) showed that a set of 1137 genes that are associated with enhancers bound by multiple islet TFs (red dots, **Table SX**) were enriched among genes that were downregulated upon knockdown of islet TFs and lncRNAs, whereas this was not observed among 10 size-matched control gene sets that are expressed at similar levels as the enhancer-associated genes (black dots). Islet enhancers bound by four or more islet TFs have been shown to be associated with core lineage-specific regulatory genes (Pasquali et al., 2014). (B) TF and lncRNA-dependent genes are often islet specific. GSEA analysis was performed with 700 genes that show highest expression compared to 16 other tissues (blue dots, **Table SX**) or 10 control size-matched gene sets that are expressed at similar levels (black dots). Y-axes represent GSEA FWER p-values (-Log10).

Figure S5. Co-expression analysis of human islet lncRNAs, related to Figure 5 (part 1 of 2)

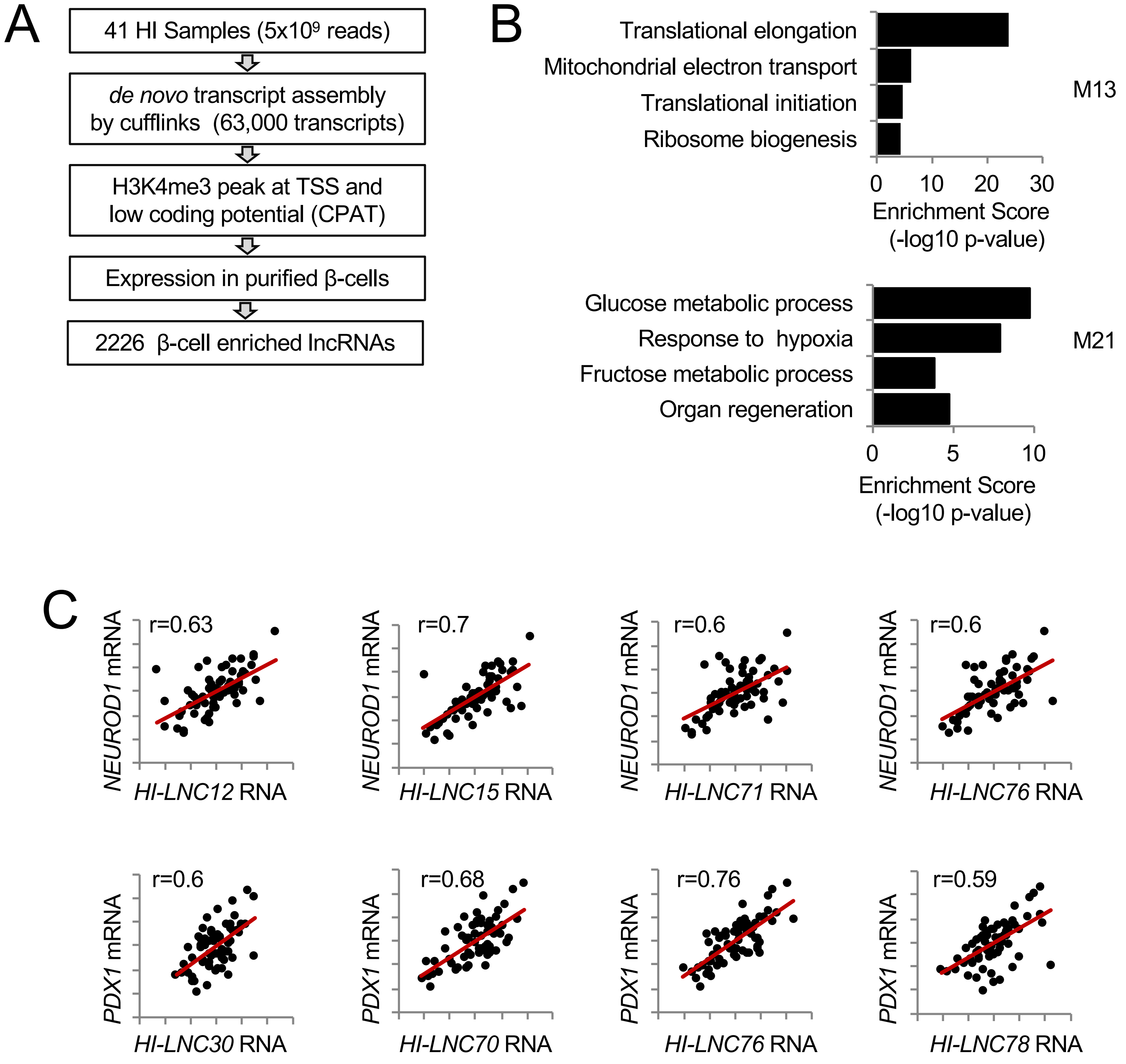

D

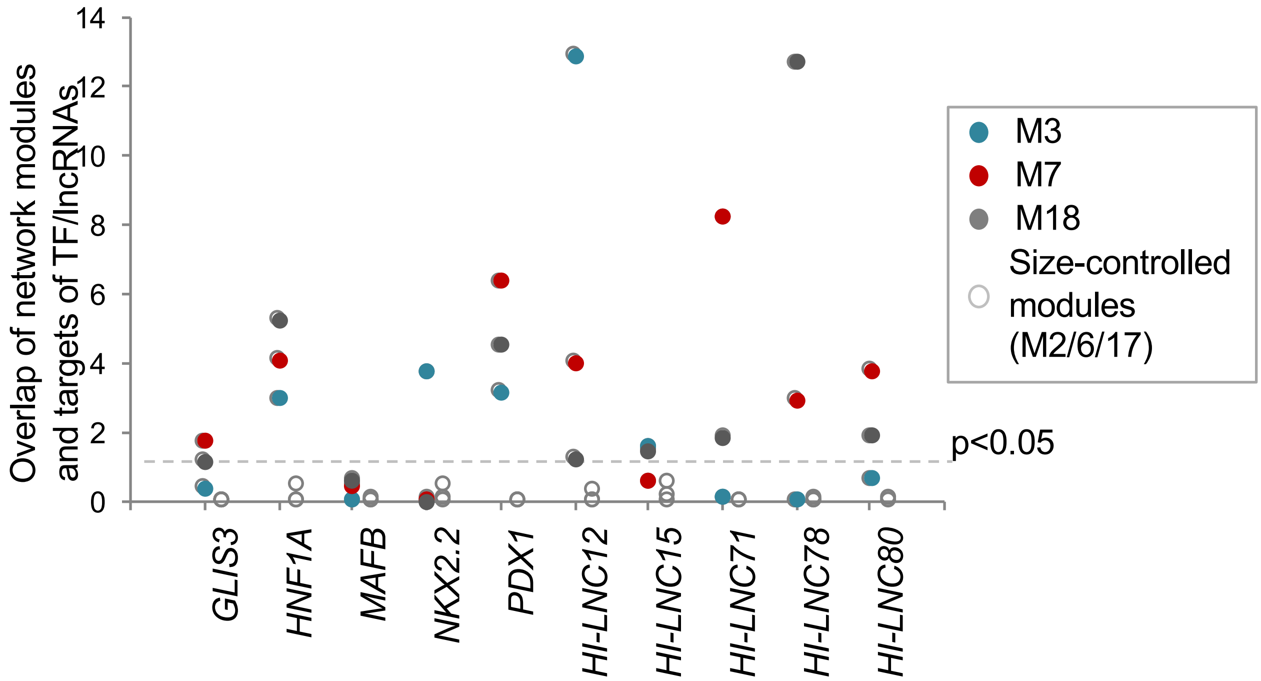

Figure S5. Co-expression analysis of human islet lncRNAs. (A). Schematic of *de novo* lncRNA annotation pipeline. To expand the previously defined list of islet lncRNAs and enhance their annotation accuracy (Moran et al, 2012), RNA from 41 human islet samples was sequenced directionally, generating nearly 5 billion reads. Cufflinks was then used to assemble *de novo* transcripts, which led to ~63,000 transcripts that were not known coding genes. These transcripts were further filtered for (i) low coding potential based on CPAT, (ii) K4me3 enrichment at a putative promoter region. (iii) expression in FACS purified β-cell samples as well as FACS purified acinar/β-cell ratio of < 3. Multiple splice variants of the same gene were collapsed into a single gene, resulting in 2226 islet lncRNA genes (**Table S3**). (**B**) Module M13 is enriched in translation and ribosomal related gene ontology annotations while M21 is enriched in terms related to glucose metabolic process (DAVID analysis, biological process). (**C**) Examples of β-cell lncRNAs that show a high correlation of expression with islet TFs *PDX1* and *NEUROD1* across 64 human islet samples. The axes are gene expression values normalized across all samples. (**D**) TFs and lncRNAs often regulate genes from lncRNA-enriched co-expression modules. Y-axis represents p value (-Log10) of hypergeometric test of overlap between the gene sets that show significant downregulation upon knockdown of indicated TF or lncRNAs (at p<10-3), and the cell-specific co-expression modules enriched in lncRNAs (M3, M7, M18). As controls, we examined the 3 modules (M2, M6, M17) that had the closest number of genes as lncRNA-enriched modules.

Figure S6. LncRNA *PLUTO* in mouse and human islets (related to Figure 6, page 1 of 3)

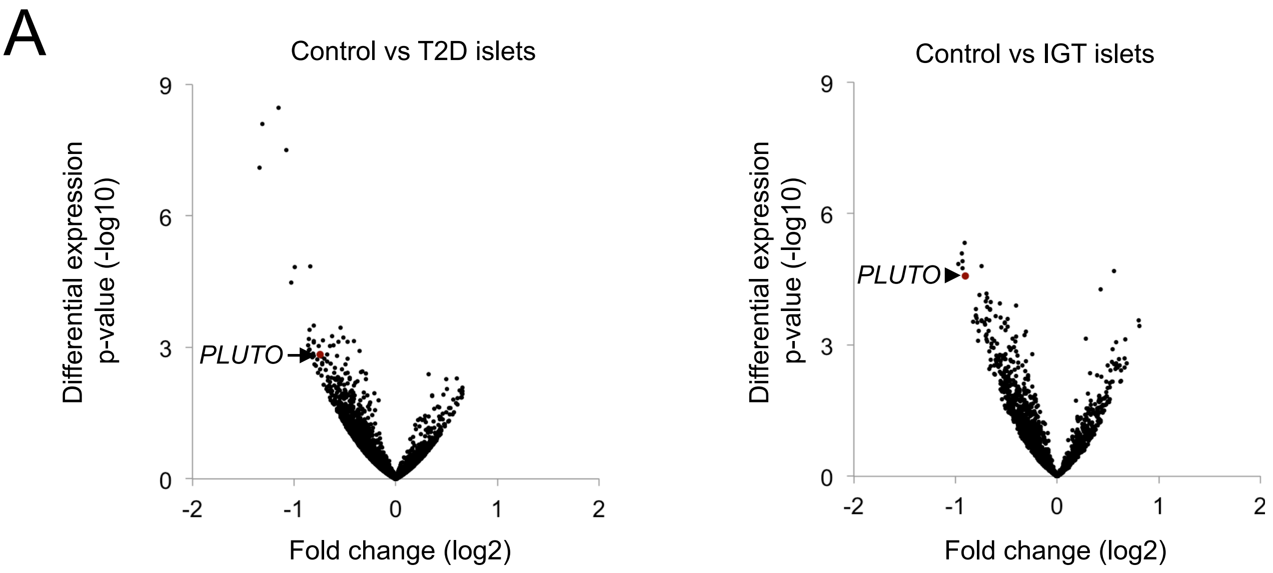

Figure S6. LncRNA *PLUTO* in mouse and human islets (related to Figure 6, page 2 of 3)

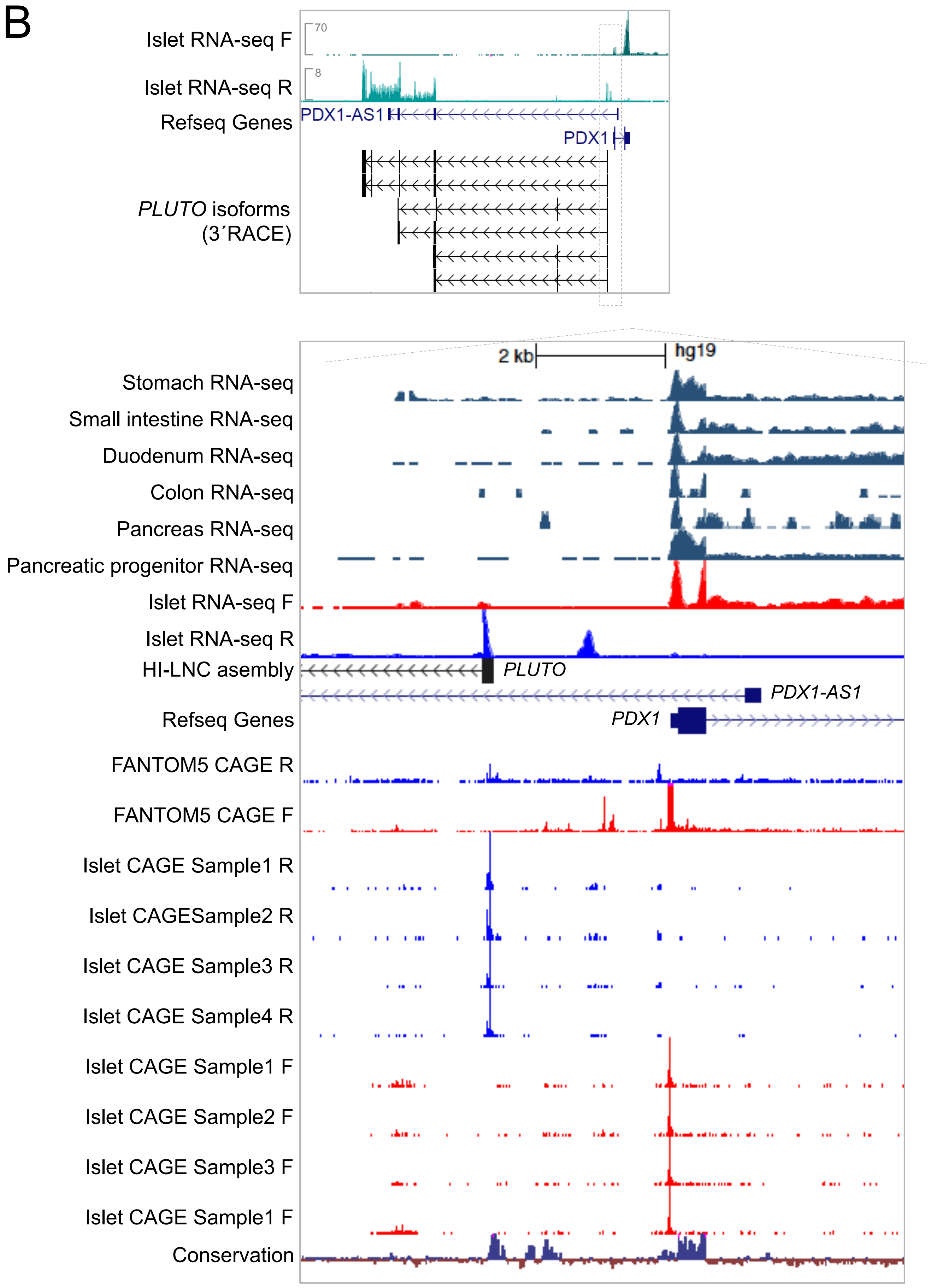

Figure S6. LncRNA *PLUTO* in mouse and human islets (related to Figure 6, page 3 of 3)

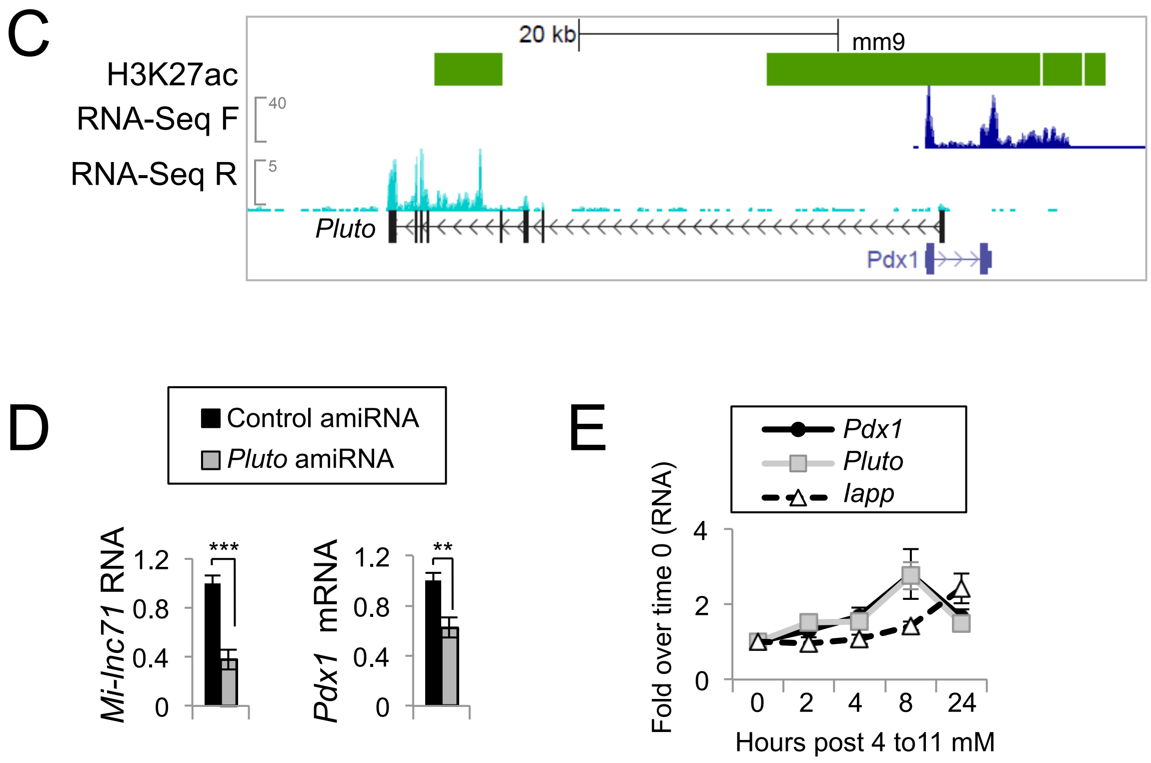

Figure S6. LncRNA *PLUTO* in mouse and human islets
(A) Differential expression analysis of human beta cell lncRNAs from control (n=50) versus T2D (n=10) or IGT (n=15) islet RNA-seq samples. Volcano plots of nominal p-value (-log10) vs Log2 fold change of expression (T2D or IGT over control) is shown. (B) Human *PLUTO* is a multi-isoform transcript. The schematic represents the isoforms cloned from 3´RACE assay. Detailed lower panel shows RNA-seq from Human Proteome Atlas (HPA) tissues, as well as pancreatic progenitor and human islet. Islet RNA-seq track represents merged reads from 56 different islet samples totaling 7.1 billion aligned, stranded reads (scales represent RPM). Shown in blue (reverse) and red (forward) are total CAGE tags from FANTOM5 consortium. CAGE tags from four independent islet samples are also shown. Conservation track shows vertebrate conservation (PhyloP). (C) Schematic of the mouse *Pdx1/Pluto* locus, showing H3K27Ac-enriched regions in mouse islets, and RNA-seq data for the forward and reverse strands. Shown in black is a cloned isoform of *Pluto*. (D) In a mouse beta cell line (MIN6), downregulation of *Pluto* using amiRNA results in reduced *Pdx1* mRNA. RNA values are normalized to *TBP* and then to control amiRNA. Error bars represent ±SEM, $**p<10^{-3}$, $*p<0.05$, Student´s t-test. (E) In mouse islets, *Pdx1* and *Pluto* RNAs are co-regulated in response to a shift in glucose levels. Mouse islets were harvested and incubated in media with 4 mM glucose for 2 days. Islets were then shifted to a higher glucose concentration (11 mM) for indicated periods of time during which both *Pdx1* and *Pluto* RNA is upregulated with similar kinetics. Shown as control, *Iapp* mRNA levels are induced with different dynamics (n=3).

Figure S7. Effects of *PLUTO* on chromatin modification landscape of *PDX1* enhancers (related to Figure 7)

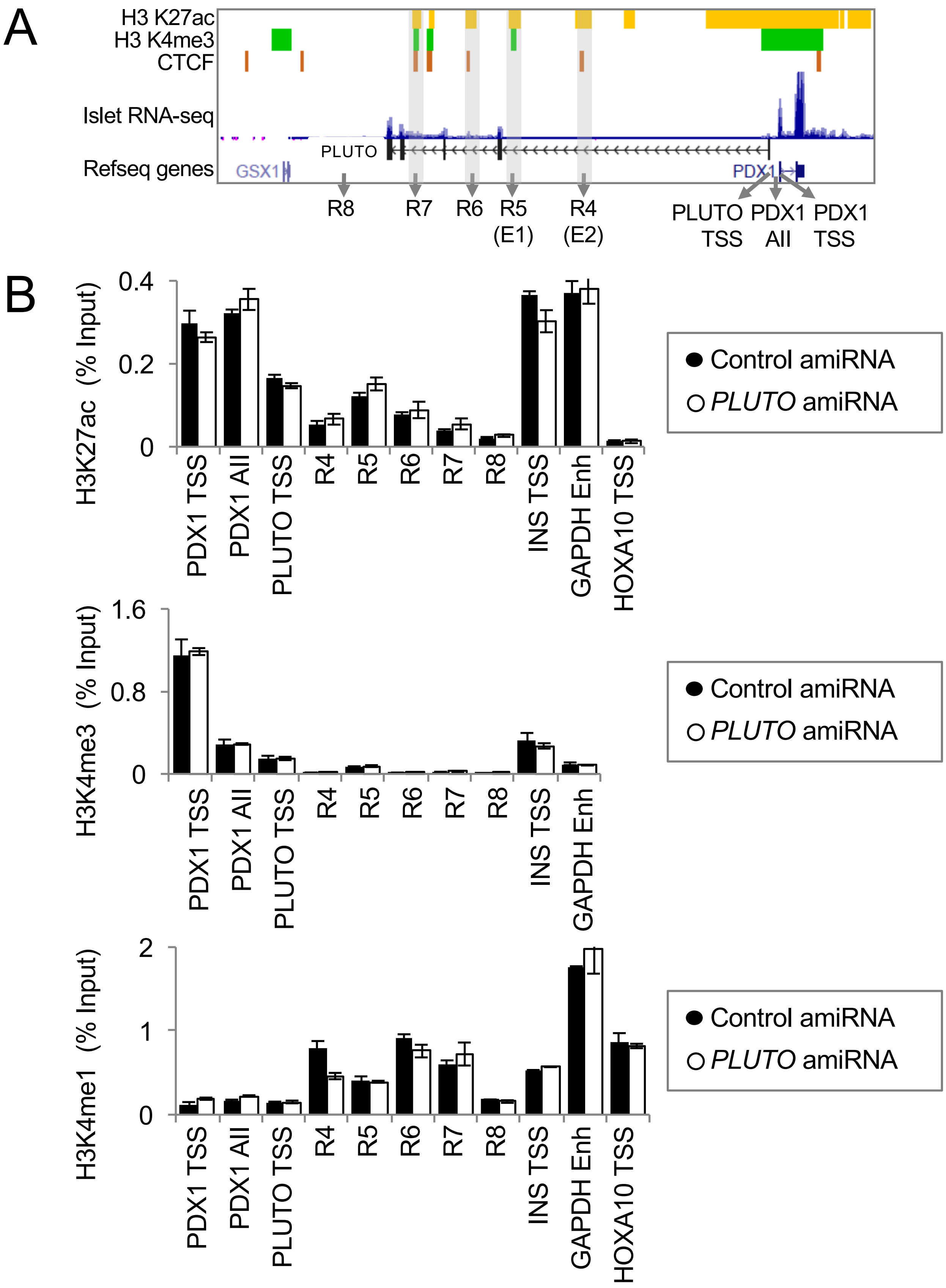

Figure S7. Effects of *PLUTO* on chromatin modification landscape of *PDX1* enhancers
(A) Schematic of *PDX1*/*PLUTO* locus displaying the position of the primer sets used for this study relative to the indicated human islet chromatin and transcript enrichments. (B) Graphs represent chromatin immunoprecipitation experiments with antibodies against H3K27 acetylation (n=5), H4K4 tri-methylation (n=3) and H3K4 mono-methylation (n=1) in EndoC-βH1 cells 80 hours post-transduction with lentiviral vectors carrying control or *PLUTO* amiRNA sequences. Values are expressed as percentage of input DNA, error bars denote ±SEM.

Supplemental Tables

Table S1. Features of human lncRNAs selected for functional analysis, related to Figures 1, S1, 6, S6.
Table S1a. Features of human and mouse beta cell lncRNAs analyzed with amiRNA knockdowns.
Table S1b. Isoforms of PLUTO (HILNC71) sequenced from 3'RACE.

Table S2. Co-expression network analysis, related to Figures 5, S5.
Table S2.1 Network modules resulting from weighted gene co-expression analysis (WCGNA) of 64 human islet RNA-seq samples
Table S2.2 Assignment of islet expressed genes to network modules resulting from weighted gene co-expression analysis (WCGNA) of 64 human islet RNA-seq samples.
Table S2.3 Assignment of lncRNAs to network modules resulting from weighted gene co-expression analysis

Table S3. List of human beta-cell lncRNAs, related to Figures S5.
Table S3a. List of de-novo annotated human beta-cell lncRNAs (bed12 format, hg19)
Table S3b. Complementary list of previously annotated lncRNAs expressed in beta cells (bed12 format, hg19)

Table S4. Sequences of amiRNAs and oligonucleotides, related to experimental procedures.
Table S4a. Targeting sequences for miR-RNAi based knockdown.
Table S4b. Primer sequences for semi-quantitative real time reverse transcription PCR used in this study.
Table S4c. Taqman probe sequences for quantitative real time reverse transcription PCR reactions used in Figures 6 and 7.
Table S4d. Primer Sequences for 3C experiments with PDX1 TSS as viewpoint.
Table S4e. Primer Sequences for chromatin immunoprecipitation experiments used in Figure S7.
Table S4f. gRNA sequences for CRISPRi used in Figure 6.
Table S4g. LNA GapmeR sequences from Exiqon used in Figure S2.

Table S5. Gene sets used for integrative analysis, related to experimental procedures.

Table S6. Human Islet LncRNAs that are highly co-expressed with a neighbouring coding gene, related to Figure S1.

Table S7. Differential expression analysis of human islet lncRNAs in islets from donors with T2D and IGT, related to Figures 6 and S6.
Table S7a Differential expression analysis of human islet lncRNAs in control vs T2D islets (DE-seq).
Table S7b Differential expression analysis of human islet LncRNAs in control vs impaired glucose tolerance (IGT) islets (DE-seq).

Supplemental experimental procedures

Human islets
Human islet experiments were performed following ethically approved protocols from Hospital Clinic de Barcelona, Geneva University Hospitals, University of Lille and Milano San Raffaele Hospital. Islets were isolated from multiorgan donors after informed consent from family members. Detailed information on a panel of human islets used for RNA-seq are provided elsewhere (Moran et al., 2012)(Moran et al, in preparation). Briefly, pancreatic islets were isolated and purified using established isolation procedures (Bucher et al., 2005; Kerr-Conte et al., 2010), and cultured prior to analysis as described (Moran et al., 2012). Islet purity was assessed by (a) dithizone staining using an aliquot of islets immediately prior to harvest, and (b) qPCR analysis of duct, acinar, and β-cell mar ers (*SOX9*, *CPA1*, *MIST1*, *PAX6*, and *INS*). Only samples showing >80% dithizone purity and marginal exocrine contaminant mRNAs were further processed.

Mouse islets
Mouse experiments were conducted following procedures approved by the Ethical Committee of Animal Experimentation of Hospital Clinic de Barcelona. Mouse islets were isolated from 10-14 week-old C57BL/6J mice (Parrizas et al., 2001). After isolation islets were incubated in 4 mM glucose RPMI 1640 medium, supplemented with 10% fetal calf serum, 100 U/ml penicillin, and 100 U/ml streptomycin for 48 hours. At 2, 4, 8 and 24 hours prior to 72 hours, the media was supplemented with glucose to achieve a final concentration of 11 mM. All islets were then harvested for RNA extraction at 72 hours. The experiment was repeated 3 times.

RNA-Seq
RNA-seq was performed with TruSeq Stranded Total RNA libraries (Illumina) generated from human pancreatic islet samples and used to generate paired-end 100 nucleotide reads.

Reverse transcription PCR analysis
RNA was extracted using Tripure reagent (Roche), RNA quality was ascertained with a 2100 Agilent Bioanalyzer before microarray analysis and RNA-sequencing (RIN>8). Prior to reverse transcription, RNA was treated with DNAseI (Sigma) for 20 minutes at room temperature. cDNA synthesis was carried out using Superscript III (Invitrogen) and real-time PCR was performed using SYBR Green detection with the ABI 7300 Real Time PCR system (van Arensbergen et al., 2010). For Figure 7B, mRNA and intronic RNA levels were measured using TaqMan assays. *TBP* or *Tbp* mRNAs were used to normalize the expression of all samples. *HPRT, ACTB* or *Hprt* mRNAs were also used as a second normalization control for most experiments, obtaining similar results (data not shown). Primers are provided in **Table S4**. For qPCR experiments bars represent average mean expression (±SEM) and p-values denote Student´s t-test calculated with GraphPad Prism software.

Cell culture
MIN6 cells were maintained in DMEM (Lonza) supplemented with 15% fetal calf serum, 100 U/ml penicillin, 100 U/ml streptomycin and 46 nM 2-Mercaptoethanol at 37°C in a humidified chamber with 5% CO_2**.** EndoC-βH1 and EndoC-βH3 cells were maintained as previously described (Ravassard et al., 2011; Scharfmann et al., 2014). EndoC-βH3 were induced to excise the T-antigen over a course of three weeks with six evenly spaced 4-Hydroxy tamoxifen (4-OHT, 1μM final) treatments. This procedure stops the cell cycle after 10-12 days. Cells were harvested between day 21 and 24. For mRNA stability experiments EndoC-βH1 cells were treated with Actinomycin D at 5 μg/ml.

Nuclear fractionation
Nuclear fractionation was done as previously described in the presence of RNAse inhibitors (Dart et al., 2004). RNA was then extracted from the fractionated and whole cell lysates using TriPure reagent.

Gene knockdown experiments using amiRNAs
Five non-targeting and four amiRNAs targeting each of 5 TF and 25 lncRNAs were designed using BLOCK-IT software (Invitrogen) and cloned into pTRIP-CMV gateway vectors as described (Moran et al., 2012) (see **Table S4** for amiRNA hairpin sequences). These were then used to produce lentiviruses, which were transduced into the EndoC-βH1/3 cells as described (Castaing et al., 2005; Scharfmann et al., 2014). The cells were harvested at 80 hours, and RNA levels were assessed using qPCR. Only amiRNAs that produced consistent inhibition of target RNAs to at least 50% in at least two consecutive experiments were considered further. Cells transduced with five different non-targeting control

amiRNAs and non-transduced cells were analyzed in parallel. Each amiRNA was transduced in duplicate in independent plates. For knockdown experiments in human islets, islets were dissociated with trypsin (0.05%), washed with PBS and transduced with lentivirus (as described for EndoC-βH1 cells), and plated onto poly-lysine coated wells with DMEM supplemented with 10% fetal calf serum, 100 U/ml penicillin and 100 U/ml streptomycin. The efficiency of transduction was judged to be >95% based on GFP expression. We found that expression of *PDX1*, *PAX6, IAPP, TXNIP* and *DDIT3* (three beta cell and two cellular stress markers) were similar in intact and dispersed islets after 80 hrs of culture.

Locked nucleic acid (LNA) and CRISPR interference (CRISPRi) experiments

Two independent LNA Gapmers targeting *HI-LNC12*, *HI-LNC80*, *HI-LNC78, HI-LNC71 (PLUTO) and PDX1* were obtained from Exiqon (sequences in **Table S4**). For CRISPRi experiments, the pSpCas9n(BB)-2A-GFP vector (a gift from Feng Zhang, Addgene plasmid # 48140) was modified into pSp-dCas9-2A-GFP by introducing the H840A mutation in Cas9 to obtain the catalytically inactive Cas9 (dCas9). Two separate CRISPRi guide RNAs were designed to target *PLUTO* exon 1, and cloned into pSp-dCas9-2A-GFP (sequences in **Table S4**) (Qi et al 2013, Gilbert et al 2014). Control CRISPRi guide RNAs were designed targeting intergenic regions. LNA gapmers (50 pmol) or CRISPRi vectors (6 μg) were nucleofected (program D-023) into 1-2 million EndoC-βH3 cells using Amaxa Cell Line Nucleofector Kit V (Lonza) and plated 80 hours prior to harvest.

Glucose stimulated insulin secretion (GSIS)

GSIS for **Figure 3** was carried out as described (Benazra et al., 2015). Secreted and total insulin was first normalized to cell number and then expressed as a fraction of levels at 2 mM control cells. For GSIS experiments in **Figure S3** (using LNA gapmers), tamoxifen treated EndoC-βH3 cells were subjected to nucleofection and plated 80 hours prior to GSIS. KREB´s buffer (115 mM NaCl, 24 mM $NaHCO_3$, 5 mM KCl, 1 mM $MgCl_2$, 1 mM $CaCl_2$,10 mM HEPES pH 7.5, 0.2 % BSA) was prepared freshly and equilibrated for 90 minutes in a humidified chamber with 5% CO_2. Cells were washed five times in warm PBS buffer and incubated with KREB´s buffer with 0.5 mM glucose for two hours. Cells were then incubated for 30 minutes with KREB´s buffer containing 0.5 mM, 6 mM or 16 mM glucose and 500 μM IBMX (Sigma). Supernatant containing secreted insulin was harvested, centrifuged (700g for 10 minutes at 4^0C) and quantified by ELISA (Mercodia, 10-1113-01). Attached cells were harvested with lysis buffer (50 mM Tris-HCl, pH8, 100 mM EDTA, 100 mM NaCl, 1% SDS, Roche protease inhibitor cocktail) and insulin was quantified by ELISA. Secreted insulin was normalized to total insulin content and expressed as fold enrichment over 0.5 mM control values. Bars represent an average of four independent experiments, and two independent control or targeting LNA mediated knockdown samples. P-values denote Student´s t-test calculated with GraphPad Prism software.

Immunoblotting

Equal amounts of protein from cell lysates were run on 10% bis-polyacrylimide gels and transferred to a PVDF membrane (0.45 μM). After blocking with 5% milk proteins in TBS - 0.02% Tween20 for one hour, the membrane was incubated overnight at 4^0 C with anti-PDX1 (Abcam, ab47383, 1:10,000) in the same buffer. Primary antibodies were detected by HRP-conjugated secondary antibodies (DAKO). Bands were detected and quantified using LAS4000 and ImageQuant software. Membrane was then stripped for reprobing with TBP (Santa Cruz, sc-273, 1:1000) followed by anti-Histone3 (Abcam, ab1791, 1:1000). PDX1 levels were then normalized by an average of TBP and H3.

Chromatin conformation capture

3C experiments were performed essentially as described (Pasquali et al., 2014; Tena et al., 2011). In brief, ~3×10^6 cells formaldehyde fixed EndoC-βH1 cells were transduced with control or targeting amiRNAs and lysed with lysis buffer (10 mM Tris-HCl pH 8, 10 mM NaCl, 0.3% IGEPAL-CA-630 (Sigma), 0.3% TritonX-100 (Sigma), 1X protease inhibitor (Complete, Roche)). Nuclei were digested with DpnII (NEB) and ligated with T4 DNA ligase (NEB). Primers were designed to amplify indicated ligated regions (**Table S4**), and were quantified by real-time PCR. Values were normalized to an intronic region of *PDX1* gene that does not contain a DpnII cut site, although similar results were obtained with or without normalization. The results were compiled from six independent experiments. Results were analyzed with Student's t-test for significance. 4C-Seq on ENDOC-βH1 cells (**Figure 7C**) was performed and analysed as previously described (Pasquali et al., 2014).

Chromatin immunoprecipitation

ChIP was performed with 1 million EndoC-βH1 cells/sample as described using the same antibodies and protocol as previously described (Pasquali et al., 2014).

***De novo* transcript assembly for islet lncRNAs**

We aligned ~5 billion stranded paired-end RNA-seq reads from 41 human islet samples to the human genome (hg19) as described (Moran et al., 2012). The reads falling into the top 143 most expressed genes were excluded. Remaining fragments were assembled into transcripts using cufflinks (Parameters: --min-isoform-fraction 0.01 --pre-mrna-fraction 0.10 --max-bundle-frags 5000000). Unannotated transcripts were extracted and retained if the following criteria were satisfied (i) presence of more than one exon in the transcript, (ii) presence of H3K4me3 enrichment (defined as described in Moran et al., 2012) in a region that is consistent with the transcriptional promoter (+1kb to -0.5 kb from the transcript 5' end), (iii) RNA Coding Potential Assessment Tool (CPAT) score < 0.364 (following recommendation for human genome sequences by (Wang et al., 2013)). For lncRNA genes with multiple isoforms, the isoform with the longest exon number was kept, or the isoform with the longest exon length in the case of multiple isoforms with the same number of exons. For 18 cufflinks-generated transcripts that did not fulfill the three above-mentioned criteria for lncRNAs, but there was a previously annotated non-coding Ensembl transcript of the same gene that fulfilled those same criteria, the latter lncRNA gene annotation was retained. We also included lncRNA genes defined by (Moran et al., 2012) that were not selected with the abovementioned annotation. Any lncRNA overlapping a coding exon on the same strand was removed from the list. Finally, the lncRNAs were filtered for expression of >0.05 FPKM in FACS purified beta cells and an acinar to beta cell expression ratio < 3, using FACS purified beta and acinar RNA-Seq data processed as described in (Moran et al., 2012). This resulted in a total number of 2226 de novo annotated lncRNA genes (**Table S3**).

We further generated a second list of 2112 previously annotated lncRNA genes, which contained Ensembl transcripts annotated by Havana as "lincRNA" or "antisense" and contained H3K4me3 enrichment in the 5' end, were expressed >0.05 FPKM in FACS purified beta cells, showed an acinar to beta cell expression ratio < 3, and had <10% overlap with any transcript from the list of 2226 de novo annotated lncRNAs (**Table S3**). This list included previously annotated lncRNAs that were not contained in the list of 2226 de novo annotated lncRNAs because they had higher CPAT scores than our thresholds, were monoexonic, overlapped a protein-coding gene, or were represented by transcripts with only minimal overlap. LncRNAs annotated in this study are available to download as a track hub on a UCSC genome browser (GRCh37/hg19) session by selecting "track hubs", and selecting "Human Islet lncRNAs". Alternatively the track hub can be directly visualized in the UCSC Genome Browser.

Differential expression analysis of IGT and T2D islets

Data was downloaded from (Fadista et al., 2014). The samples were aligned using STAR aligner version 2.3.0 with hg19 genome in which common SNPs (Global Minor Allele Frequency>1%) from the dbSNP database 142 were masked (Dobin et al., 2013; Sherry et al., 2001). A maximum mismatch of 10 nucleotides was used, and non-uniquely aligned reads were removed. Quantification of the raw read count was done using HTseq-Count version 0.6.1 with python 2.6.6 and Pysam version 0.8.3 (Anders et al., 2015). Differential expression analysis was done using DEseq2 version 1.10 on lncRNA genes (Table S3), using an adjusted p-value threshold of 0.05, and after removing one T2D outlier sample based on PCA (Love et al., 2014).

Co-expression network analysis

Sequence reads were aligned to the human reference genome hg38 with TopHat v.2.0.12, using Bowtie2(v.2.2.3) GENCODE(v21) and a customized lncRNA annotation were used as reference for mapping. Cufflinks (v2.2.1) was used to obtain FPKM values for gene and lncRNA expression(Harrow et al., 2012; Trapnell et al., 2012). Co-expression networks were built on mRNA expressions from 64 human islet samples using WGCNA(v2) package in R (Zhang and Horvath, 2005). Specifically, we first restricted the mRNAs into protein coding genes from HUGO Gene Nomenclature Committee (HGNC, downloaded on 5-19-2015), we then removed mRNAs that were not expressed in more than 50% samples. We inverse quantile normalized mRNA expressions, i.e., for each mRNA, we mapped its expression levels across samples into a vector generated from standard normal distribution $N(0,1)$ based on the rank of expressions across 64 samples). Finally, we corrected the batch effect using a linear regression model. The processed data were applied into WGCNA with power 6 to construct co-expression modules. The power of 6 was chosen using the scale-free topology criterion(Zhang and Horvath, 2005).

lncRNAs (**Table S3**) were filtered, normalized and batch effect corrected the same way as mRNAs. Only 2455 lncRNAs expressed in at least 50% of islet samples were used in this analysis. We assigned lncRNAs to the mRNA-derived co-expression network modules by calculating the correlation between lncRNA expression and module-gene (i.e., the first principal component of each module expression), and assigned the lncRNA to the module with the highest correlation with FDR less than 0.05.

Enrichment of lncRNAs in modules was calculated as observed over expected number of lncRNAs per module. Enrichment p-value was calculated for modules larger than 110 genes using the hypergeometric test in R.

Microarray analysis
RNA was hybridized onto HTA2.0 Affymetrix arrays. RMA normalisation was carried out using Expression Console software (Affymetrix). Gene based differential expression analysis was done using Transcriptome Analysis Console (TAC v1.0.24, Affymetrix), using RefSeq genes and a threshold ANOVA $p < 10^{-3}$ for calling differential gene expression. Raw data (.cel files) as well as analyzed microarray files (.chp) can be found at Gene Expression Omnibus (GEO, accession: GSE83619).

Cluster analysis
Genes that were dysregulated with ANOVA $p < 10^{-3}$ in at least one knockdown were included in the analysis. Genes were clustered based on Log2 of fold change of expression in control versus knockdown samples using Cluster3(v1.52) software with Euclidean Distance and Average Linkage analysis (de Hoon et al., 2004). Similar results were obtained using Centroid linkage (data not shown). Clustering was visualized using Treeview (v1.1.6) software.

Correlation analysis
For the heatmaps shown in **Figure 3A**, we selected all genes that are significantly deregulated ($p<10^{-3}$) in each pairwise comparison, and calculated Pearson´s r values based on correlation of fold change of expression for each gene upon knockdown of both lncRNA or TF. To assess correlation of transcriptional changes in pairwise comparisons of knockdowns (**Figure 3C**), we analyzed the top 100 targets from each knockdown and calculated Pearson's correlation r values for fold change (Log2) of expression. **Figure S3** displays p-values for Pearson´s correlation for the top 250 dysregulated genes in each TF or lncRNA knockdown in the vertical column of the heatmap. Top 250 dysregulated genes are defined as differentially expressed genes with the lowest 250 ANOVA p-values. All calculations were implemented using R software.

Gene set enrichment analysis (GSEA)
We used a previously defined subset of 694 enhancer cluster genes, which are active in human pancreatic islets and are linked to islet enhancer clusters that show high occupancy by islet TFs (PDX1, FOXA2, NKX2-2, NKX6.1, MAFB) (Pasquali et al., 2014). We further defined a set of 1139 genes that contained islet enhancers bound by at least four islet TFs within 25 Kb of the transcriptional start site, regardless of whether they show enhancer clustering, or defined genes that contained PDX1-bound enhancers in the same space, essentially as described (Pasquali et al., 2014)(**Table S5**). Genes associated with human islet enhancer clusters have been previously shown to include a major fraction of genes important for pancreatic islet differentiation or function (Pasquali et al., 2014). Using a previously islet specificity score (Moran et al., 2012) we have also generated a list of 700 genes that are expressed at least two standard deviations higher in human islets than the average expression value in a panel of 16 human tissues (**Table S5**).

Gene Set Enrichment Analysis (GSEA v2.1.0)(Subramanian et al., 2005) was used to calculate enrichments of gene sets amongst deregulated genes. For the analysis of each gene set, ten additional gene sets of the same size were generated based on bin-normalized expression levels in EndoC-βH1 cells. One target and 10 control gene sets were then run against RMA normalized expression data from knockdown and control samples on GSEA (SignalvsNoise, weighted). For the significance of gene set enrichment we display FWER p-values for normalized enrichment score, which provides a conservative estimation of the probability of a false discovery.

Enrichment analysis of functional annotations
For gene ontology analysis, DAVID (Huang da et al., 2009) was used with all Refseq genes as background. Functional clusters were derived from "Biological Process" category and were displayed with their respective Enrichment score, which provides a p-value for each functional cluster (negative Log10).

Supplemental references

Anders, S., Pyl, P.T., and Huber, W. (2015). HTSeq--a Python framework to work with high-throughput sequencing data. Bioinformatics *31*, 166-169.

Benazra, M., Lecomte, M.J., Colace, C., Muller, A., Machado, C., Pechberty, S., Bricout-Neveu, E., Grenier-Godard, M., Solimena, M., Scharfmann, R., et al. (2015). A human beta cell line with drug inducible excision of immortalizing transgenes. Molecular metabolism *4*, 916-925.

Bucher, P., Mathe, Z., Morel, P., Bosco, D., Andres, A., Kurfuest, M., Friedrich, O., Raemsch-Guenther, N., Buhler, L.H., and Berney, T. (2005). Assessment of a novel two-component enzyme preparation for human islet isolation and transplantation. Transplantation *79*, 91-97.

Castaing, M., Guerci, A., Mallet, J., Czernichow, P., Ravassard, P., and Scharfmann, R. (2005). Efficient restricted gene expression in beta cells by lentivirus-mediated gene transfer into pancreatic stem/progenitor cells. Diabetologia *48*, 709-719.

Dart, D.A., Adams, K.E., Akerman, I., and Lakin, N.D. (2004). Recruitment of the cell cycle checkpoint kinase ATR to chromatin during S-phase. The Journal of biological chemistry *279*, 16433-16440.

de Hoon, M.J., Imoto, S., Nolan, J., and Miyano, S. (2004). Open source clustering software. Bioinformatics *20*, 1453-1454.

Dobin, A., Davis, C.A., Schlesinger, F., Drenkow, J., Zaleski, C., Jha, S., Batut, P., Chaisson, M., and Gingeras, T.R. (2013). STAR: ultrafast universal RNA-seq aligner. Bioinformatics *29*, 15-21.

Fadista, J., Vikman, P., Laakso, E.O., Mollet, I.G., Esguerra, J.L., Taneera, J., Storm, P., Osmark, P., Ladenvall, C., Prasad, R.B., et al. (2014). Global genomic and transcriptomic analysis of human pancreatic islets reveals novel genes influencing glucose metabolism. Proceedings of the National Academy of Sciences of the United States of America *111*, 13924-13929.

Harrow, J., Frankish, A., Gonzalez, J.M., Tapanari, E., Diekhans, M., Kokocinski, F., Aken, B.L., Barrell, D., Zadissa, A., Searle, S., et al. (2012). GENCODE: the reference human genome annotation for The ENCODE Project. Genome research *22*, 1760-1774.

Huang da, W., Sherman, B.T., and Lempicki, R.A. (2009). Bioinformatics enrichment tools: paths toward the comprehensive functional analysis of large gene lists. Nucleic acids research *37*, 1-13.

Kerr-Conte, J., Vandewalle, B., Moerman, E., Lukowiak, B., Gmyr, V., Arnalsteen, L., Caiazzo, R., Sterkers, A., Hubert, T., Vantyghem, M.C., et al. (2010). Upgrading pretransplant human islet culture technology requires human serum combined with media renewal. Transplantation *89*, 1154-1160.

Love, M.I., Huber, W., and Anders, S. (2014). Moderated estimation of fold change and dispersion for RNA-seq data with DESeq2. Genome biology *15*, 550.

Moran, I., Akerman, I., van de Bunt, M., Xie, R., Benazra, M., Nammo, T., Arnes, L., Nakic, N., Garcia-Hurtado, J., Rodriguez-Segui, S., et al. (2012). Human beta cell transcriptome analysis uncovers lncRNAs that are tissue-specific, dynamically regulated, and abnormally expressed in type 2 diabetes. Cell metabolism *16*, 435-448.

Parrizas, M., Maestro, M.A., Boj, S.F., Paniagua, A., Casamitjana, R., Gomis, R., Rivera, F., and Ferrer, J. (2001). Hepatic nuclear factor 1-alpha directs nucleosomal hyperacetylation to its tissue-specific transcriptional targets. Molecular and cellular biology *21*, 3234-3243.

Pasquali, L., Gaulton, K.J., Rodriguez-Segui, S.A., Mularoni, L., Miguel-Escalada, I., Akerman, I., Tena, J.J., Moran, I., Gomez-Marin, C., van de Bunt, M., et al. (2014). Pancreatic islet enhancer clusters enriched in type 2 diabetes risk-associated variants. Nature genetics *46*, 136-143.

Ravassard, P., Hazhouz, Y., Pechberty, S., Bricout-Neveu, E., Armanet, M., Czernichow, P., and Scharfmann, R. (2011). A genetically engineered human pancreatic beta cell line exhibiting glucose-inducible insulin secretion. The Journal of clinical investigation *121*, 3589-3597.

Scharfmann, R., Pechberty, S., Hazhouz, Y., von Bulow, M., Bricout-Neveu, E., Grenier-Godard, M., Guez, F., Rachdi, L., Lohmann, M., Czernichow, P., et al. (2014). Development of a conditionally immortalized human pancreatic beta cell line. The Journal of clinical investigation *124*, 2087-2098.

Sherry, S.T., Ward, M.H., Kholodov, M., Baker, J., Phan, L., Smigielski, E.M., and Sirotkin, K. (2001). dbSNP: the NCBI database of genetic variation. Nucleic acids research *29*, 308-311.

Subramanian, A., Tamayo, P., Mootha, V.K., Mukherjee, S., Ebert, B.L., Gillette, M.A., Paulovich, A., Pomeroy, S.L., Golub, T.R., Lander, E.S., et al. (2005). Gene set enrichment analysis: a knowledge-based approach for interpreting genome-wide expression profiles. Proceedings of the National Academy of Sciences of the United States of America *102*, 15545-15550.

Tena, J.J., Alonso, M.E., de la Calle-Mustienes, E., Splinter, E., de Laat, W., Manzanares, M., and Gomez-Skarmeta, J.L. (2011). An evolutionarily conserved three-dimensional structure in the vertebrate Irx clusters facilitates enhancer sharing and coregulation. Nature communications *2*, 310.

Trapnell, C., Roberts, A., Goff, L., Pertea, G., Kim, D., Kelley, D.R., Pimentel, H., Salzberg, S.L., Rinn, J.L., and Pachter, L. (2012). Differential gene and transcript expression analysis of RNA-seq experiments with TopHat and Cufflinks. Nature protocols *7*, 562-578.

van Arensbergen, J., Garcia-Hurtado, J., Moran, I., Maestro, M.A., Xu, X., Van de Casteele, M., Skoudy, A.L., Palassini, M., Heimberg, H., and Ferrer, J. (2010). Derepression of Polycomb targets during pancreatic organogenesis allows insulin-producing beta-cells to adopt a neural gene activity program. Genome research *20*, 722-732.

Wang, L., Park, H.J., Dasari, S., Wang, S., Kocher, J.P., and Li, W. (2013). CPAT: Coding-Potential Assessment Tool using an alignment-free logistic regression model. Nucleic acids research *41*, e74.

Zhang, B., and Horvath, S. (2005). A general framework for weighted gene co-expression network analysis. Statistical applications in genetics and molecular biology *4*, Article17.
